# Supplementary material for: Modulation of working memory duration by synaptic and astrocytic mechanisms
Source: PLoS Comput Biol. 2022 Oct 3;18(10):e1010543. doi: 10.1371/journal.pcbi.1010543 (PMC9560596; doi:10.1371/journal.pcbi.1010543)
Supplement: S1 Text — We provide additional computations, numerical simulations and descriptions of the network models and their parametrizations. (PDF) [file pcbi.1010543.s001.pdf]

S1 Text

Spiking network: model and parameters

STP spiking model. The STP spiking network (used in Fig 4G-I) consists of three populations of leaky integrate-and-fire (LIF) neurons: one small excitatory population that is selective to the WM cue ($E_{\text{selective}}$), one larger excitatory population that is non-selective to the WM cue ($E_{\text{unselective}}$), and one global inhibitory population I (Fig 1C). For better consistency and comparability with previous work, the number of neurons in each population, including the ratio of selective to unselective excitatory neurons, was chosen as in the studies by Mongillo et al. [1] and Mi et al. [2]. Note that the inhibitory population is needed to balance the activity of the excitatory population – the interplay of excitatory and inhibitory activity assures that the overall network activity stays in a balanced regime and neither goes to zero nor explodes. All neurons in the network are randomly and sparsely connected, and all synaptic connections have fixed synaptic weights J, i.e. there is no long-term synaptic plasticity in the network. However, the synaptic efficacy $g(t) = Jx(t)y(t)$ of the excitatory-to-excitatory (E-E) connections (marked in red in Fig 1C) underlies short-term synaptic plasticity (STP) dynamics as described in Eqs. 1 and 2. All neurons in the network receive an excitatory external baseline input current with constant, population-specific mean and Gaussian white noise. We model the presentation of a WM item to the network as a temporary increase ($T_{\text{cue}} = 100ms$) of the mean external input into the excitatory selective population. After the presentation of the WM cue, the mean excitatory input into the excitatory selective population is reduced back to its baseline value. The network response after the offset of the WM cue (i.e. during the 'delay period') is what we refer to as WM representation. Note that we do not explicitly simulate the learning and long-term plasticity through which a selective sub-population is formed but assume that this has taken place before the onset of our WM simulations. The reason for this setup is that in this study, exclusively look at the effects of astrocytic modulation on short-term synaptic plasticity. All network parameters except the STP parameters U, τ_F and τ_D were chosen as in the original model by Mongillo et al. [1] and the follow-up work by Mi et al. [2]. STP parameters were varied as part of our study and are listed in Table B in S1 Text along with all other, fixed network parameters. Table A in S1 Text provides a more detailed description and discussion of the model in the format of the reproducibility framework by Nordlie et al. [3].

STP+LPA spiking network. The STP+LPA spiking network (used in Figs 5D-F and 6G-I) has the same architecture and uses the same fixed network parameters and WM protocol as the STP spiking network. The only difference between the two network models is that the E-E synapses in the STP+LPA spiking network underlie STP with astrocytic modulation as determined by our tripartite synapse model with LPA signaling (Eqs. 1, 2, 4, 5), while the E-E synapses in the STP spiking model only have STP dynamics (Eqs. 1, 2) without astrocytic modulation. Note that our current PRG spiking network does not model astrocyte-to-astrocyte interactions but assumes that an astrocyte is synapse-specific, i.e. regulates the activity of a single synapse. Vice versa, each synapse receives individual presynaptic activity and interacts with an independent astrocyte or astrocytic compartment. Communication between astrocytes or astrocytic compartments surrounding different synapses is currently not considered in the model. Because we assume all synapses of a sub-population to be statistically identical (except for the connection weights, which can differ) we can define an average synaptic and astrocytic state.

In Table A in S1 Text, we describe the spiking neuronal network model according to the model reproducibility framework proposed by Nordlie et al. [3]. The parameter values of the model are specified in Table B in S1 Text. We implemented the model in C++ (code available at `https://gitlab.rlp.net/braincodepublished/neuronal-network-simulations-June-2021`).

Firing rate network: model and parameters

STP rate model. The STP rate model (used in Figs 3 and 4A-F) consists of a single excitatory population that corresponds to the excitatory selective population in the STP spiking model (Fig 1D). This excitatory population is connected to itself through a recurrent synapse with STP, where the STP dynamics are defined analogously to STP in the spiking network (Eqs. 1, 2) as

$$\tau_F \frac{dy}{dt} = U - y + U(1-y)E + \xi_y(t) \tag{6}$$

$$\tau_D \frac{dx}{dt} = 1 - x - yxE + \xi_x(t) \tag{7}$$

with parameters U (rate of presynaptic calcium binding), τ_F (time constant of calcium unbinding) and τ_D (time constant of neurotransmitter replenishment) as in the STP spiking model. The activity of the excitatory rate population is described by the population activity E with dynamics

$$\tau \frac{dE}{dt}(t) = -E + g(JyxE + I_{\text{base}}) + \xi_E, \tag{8}$$

where τ is the time constant of the population activity dynamics I_{base} is the external baseline input current to the population. The nonlinear gain function g is given by $g(z) = \alpha \log(1 + \exp(z/\alpha))$. Note that in contrast to the standard STP rate model without noise, we add Gaussian white-noise perturbations $\xi_{x,y,E}(t)$ with mean zero and autocorrelation $\langle \xi_{x,y,E}(t)\xi_{x,y,E}(t')\rangle = \sigma^2_{x,y,E}\delta(t-t')$ to one or all of the synaptic and network variables. We analyze the system under different sources of noise in the main text in Fig 3. As in the STP spiking network, a WM stimulus is presented by temporarily increasing the mean of the external baseline input for the duration of the stimulus. However, while the external input reflects the combined inputs from inhibitory and excitatory presynaptic populations, the cue presentation increases only the excitatory (not the inhibitory) part of the input. All rate model parameters are given in Table D in S1 Text; a detailed model description can be found in Table C in S1 Text.

STP+LPA rate network. Analogous to the spiking network case, the STP+LPA rate model (used in Figs 5A-C and 6A-F) only differs from the STP rate model in the synaptic dynamics of the recurrent self-connections. While the recurrent self-connections in the STP rate model are governed by the STP without astrocytic modulation (Eqs. 6, 7, 8), they underlie astrocytically modulated STP in the STP+LPA rate model. Analogous to the STP+LPA spiking network, the astrocytic modulation of the STP calcium binding rate U in the STP+LPA rate model synapses is given by

$$U = U_b + \Delta U \ell \tag{9}$$

$$\tau_L \frac{d\ell}{dt} = -\ell + M(1-\ell)yxE \tag{10}$$

with parameters U_b (baseline rate of presynaptic calcium binding), ΔU (maximal increase of U with LPA binding), M (probability of LPA binding) and τ_L (time constant of LPA unbinding).

Analogously to the spiking network model, Table C in S1 Text depicts the firing rate network used in Figs 3 and 4A-F. The rate model parameters are given in Table D in S1 Text.

Separation of timescales for the rate model. For Fig 6D-F, we perform a scale-separation of the slow vs. fast variables. In particular, we assume that $\tau, \tau_d \ll \tau_F, \tau_L$. This allows us to parametrize E and x with y (see Fig J in S1 Text). Without noise, the slow variables evolve as

$$\tau_F \frac{dy}{dt} = U - y + U(1-y)\langle E\rangle \tag{11a}$$

$$U = U_b + \Delta U \ell \tag{11b}$$

$$\tau_L \frac{d\ell}{dt} = -\ell + M(1-\ell)y\langle Ex\rangle, \tag{11c}$$

where $\langle E\rangle$ and $\langle Ex\rangle$ are averages of E and Ex over time for a constant value y. These average values are obtained by solving the firing-rate equations for E and x (see Fig J in S1 Text). In Fig K in S1 Text, we compare the solution to the full equation with the approximation of separated time scales, showing a good agreement for the oscillatory and the silent regime.

Biological evidence for slow LPA timescales

In the Models and Methods section of the main text, we cite Berridge et al. [4] as experimental evidence for the long timescales of LPA-mediated effects at the presynapse. Here, we discuss in detail how their findings support our assumption of slow LPA-mediated STP modulation.
Berridge et al. [4] discusses Ca2+ transients that decay over long timescales. This includes the Ca2+ transients that are triggered by LPA binding to the presynaptic LPA2 receptors. In particular, they write: *'The dynamics of Ins(1,4,5)P3 production can be very different depending on the receptor type being activated6. Bradykinin and neurokinin A receptors give large rapid Ca2+ transients, whereas lysophosphatidic acid (LPA), thrombin and histamine receptors give smaller responses that develop slowly but persist for much longer'* [4]. For the duration of these LPA2-R induced intracellular Ca2+ transients, the higher availability of Ca2+ in the presynaptic compartment naturally increases the probability of Ca2+ binding to the presynaptic active zones. Since the presynaptic release

of neurotransmitters is mediated via calcium binding to the presynaptic active sites, it is biologically plausible to assume that the LPA-induced increase of the presynaptic release probability persists on similar timescales as the LPA-induced intracellular Ca2+ transients that cause increased presynaptic Ca2+ binding to the active sites. In the model, this is represented as an ℓ-dependent increase of the Ca2+ binding probability U that (since ℓ and U are linearly related) persists on longer timescales τ_L.

Potential impact of PRG-associated pathologies on WM

In the context of functional and metabolic roles of astrocyte-modulated WM, it is also relevant to consider how pathologies of astrocytic signaling could negatively affect the function of WM. For example, genetic ablation of the presynaptic molecule CHL1 in mice [5] has been shown to cause reduced WM durations. CHL1 is involved in presynaptic vesicle recycling and, like PRG1 in the LPA signaling mechanisms, it directly affects synaptic transmission and short-term plasticity. For the LPA signaling mechanism that we consider in this study, the effects on WM are not yet known. However, it is known that in PRG1-KO mice, the dysfunction of the PRG1 protein prevents LPA-uptake from the cleft while the ATX-mediated synthesis of new LPA remains unaffected. As a result, the LPA concentration rises and allows a pathologically high amount of LPA to bind to presynaptic receptors. We can capture such scenarios in our model by applying different M values to model WT (low M), partial PRG-KO (intermediate M) and full PRG-KO (high M) condition. In the steady state, elevated M values lead to an increase of the effective baseline release probability U. As shown in Fig B in S1 Text, the synaptic model then reproduces qualitatively the decrease of the PPR of pathological (PRG1-KO) versus healthy (WT) conditions in depressing and facilitating synapses. In a spiking network, the increase of the effective baseline release probability U leads to increased spontaneous network activity (Fig C in S1 Text). This can be linked to the hyperexcitability measured in PRG1-KO mice, where it leads to seizures and impaired sensory processing [6].

Table A. Spiking network model.

**Model summary**	
**Populations**	Three: excitatory unselective ($E_{\text{unselective}}$), excitatory selective ($E_{\text{selective}}$), inhibitory (I)
**Connectivity**	Sparse, random connectivity
**Neuron model**	Leaky integrate-and-fire (LIF) neuron with fixed voltage threshold, reset potential and absolute refractory time
**Synapse model**	Two types in each network: non-plastic δ-synapse, short-term plastic synapse (either STP or STP+LPA)
**Input**	Independent white noise current input into each neuron, additional working memory cue input into neurons of $E_{\text{selective}}$
**Measurements**	Spiking activity in the excitatory selective population E_{seletive}, state variables of the plastic synapses connecting two excitatory selective neurons

**Populations**			
**Name**	**Elements**	**Size**	**Function**
$E_{\text{unselective}}$	LIF neurons	$N_{E,us}$	Large excitatory population that is unselective to the WM cue
$E_{\text{selective}}$	LIF neurons	$N_{E,s}$	Smaller excitatory population that is selective to the WM cue and can maintain a WM representation during the delay period
I	LIF neurons	N_I	Global inhibitory population

**Connectivity**	
**Randomness**	Random connectivity is generated by the following algorithm: For each potential connection between a target neuron $x_{i,\alpha}$ from population α and a source neuron $x_{j,\beta}$ from population β, we draw a random number from a uniform distribution between zero and one. If the number is below a threshold $p \in [0,1]$, we establish a connection between the source and the target, otherwise the source and target remain unconnected. The total number of source neurons for each target neuron in a network of N neurons therefore follows a binomial distribution with mean $p(N-1)$. We call p the connection probability of the network.
**Sparseness**	Neurons are sparsely connected in the following sense: The average number of incoming connections $C = p(N-1)$ for each neuron is significantly smaller than the total number of neurons N in the network, as $C/N = p \ll 1$. Due to the sparseness of the network connectivity, neurons receive inputs only from a relatively small number of neurons. The inputs into each neuron are therefore only very weakly correlated, such that we can neglect input correlations in sufficiently large networks.
**Self-/ Multi-connectivity**	Connections are only established between pairs of distinct neurons (no self-connectivity). Each pair of neurons can only linked by a single synaptic connection (no multiconnectivity).
**Weights**	Fixed weights $J^{(j,i)}_{\beta,\alpha}$ for each connection between a source neuron i in population α and a target neuron j in population β. Connection weights are homogeneous within each synapse population, i.e. the set of synapses with common source and target neuron populations. The only exception are synapses emerging from $E_{\text{unselective}}$ into $E_{\text{selective}}$: to reflect the variability of connection strength in very large synaptic populations like this one, each connection has probability γ_{pot} to be assigned with a potentiated weight J_{pot} instead of the baseline connection strength J_{base}.
**Delays**	All synapses connecting neurons within one population have non-zero synaptic delay times. For each such connection, a delay time is drawn uniformly at random from the interval $[D_{\text{min}}, D_{\text{max}}]$.

Neuron model	
**Type**	Leaky integrate-and-fire (LIF) neuron. Each LIF neuron is characterized by its membrane potential $V(t)$.
**Subthreshold dynamics**	The dynamics of the membrane potential V_i of an LIF neuron i in population α between two consecutive spike times t_k and t_{k+1} are given by:<br>$$\frac{dV_i}{dt}(t) = -\frac{V_i}{\tau}(t) + \sum_{j \neq i} I_{ij}(t) + I_{\mathrm{ext},i}(t) \qquad \text{if } t_k + t_{\mathrm{ref}} < t < t_{k+1}$$<br>$$V_i(t) = V_r \qquad \text{if } t_k < t < t_k + t_{\mathrm{ref}}.$$<br>Here, τ is the membrane time constant of the LIF neuron, V_r is its reset potential andt_{ref} is its refractory period. The neuron receives recurrent synaptic inputs $I_{ij}(t)$ through synapses from other neurons j to neuron i and an external white noise input $I_{\mathrm{ext},i}(t)$. We assume that the membrane resistance R is constant and can therefore be included in the representation of the currents.
**Spiking**	A neuron i emits a spike when its membrane potential $V_i(t)$ crosses the threshold potential V_θ. The time point of the k-th spike emission is saved as spike time t_k and the membrane potential is set to the reset potential V_r.

Synapse models	
**Name**	**Dynamics**
$\delta-$synapse	A δ-synapse from neuron j in population β to neuron i in population α transmits the synaptic current<br>$$I_{ij}(t) = J_{\alpha\beta}^{(ij)} \sum_k \delta(t - D_{ij} - t_k),$$<br>where $J_{\alpha\beta}^{(ij)}$ is the synaptic connection strength, D_{ij} is the synaptic delay and t_k is k-th spike time of the presynaptic neuron j.
STP synapse	See Eqs. 1, 2, 3.
STP+LPA synapse	See STP synapse, extended with Equations 4, 5.

Input	
**Baseline input**	Every neuron i from population α receives an excitatory baseline input with constant mean μ_α,<br>$$I_{ext,i} = \mu_\alpha + \xi_i(t),$$<br>where $\xi_i(t)$ is Gaussian white-noise with mean zero and autocorrelation $\langle \xi(t)\xi(t') \rangle = \sigma_\alpha^2 \delta(t - t')$.
**Cue stimulation**	Neurons of the excitatory selective population E_s receive a short, additional input current that represents a stimulation of the network with a working memory cue. After an initial period T_{init} of baseline stimulation that allows the network to settle into stable dynamics, the mean and variance of the white-noise input into E_s is increased by a factor A_{cue} for a duration of T_{cue}. Afterwards, the white-noise input into E_s returns to baseline stimulation for the remainder of the simulation.

Measurements	
**Type**	**Method**
Population firing rate	Sum of spike counts in each population per second
Duration of population spike regime	Time between end of the cue stimulation and the last population spike. We use an in-built Matlab function for spike detection.
Statistics of population activity duration	Statistics of the duration of population spike activity are computed for a set of S realizations of the same network. Sources of randomness that lead to differences across realizations are the random connectivity structure of the network, the initialization of synaptic and neuronal variables and temporal noise of the external input. The randomness in these sources is set via independent random seeds that are generated from a global random seed. The global random seed is chosen uniquely for each realization.

Table B. Standard parameters for the spiking network model, if not stated otherwise.

**General simulation parameters**	**Values**	
dt – simulation time step	0.1 ms	
recording bin size	10 ms	
S – number of simulated realizations	100	
**Populations parameters**	**Values**	
N – total number of neurons	10 000	
$N_{E,us}$ – number of excitatory unselective neurons	7 200	
$N_{E,s}$ – number of excitatory selective neurons	800	
N_I – number of inhibitory neurons	2 000	
**Connectivity parameters**	**Values**	
p – connection probability	0.2	
J_{IE} – synaptic efficacy $E \to I$	0.135 mV/spike	
J_{EI} – synaptic efficacy $I \to E$	0.25 mV/spike	
J_{II} – synaptic efficacy $E \to I$	0.2 mV/spike	
J_{base} – baseline level of $E \to E$ synaptic efficacy	0.05 mV/spike	
J_{pot} – potentiated level of $E \to E$ synaptic efficacy	0.45 mV/spike	
γ_{pot} – fraction of potentiated synapses	0.1	
$[D_{\mathrm{min}}, D_{\mathrm{max}}]$ – range of synaptic delays	[0.1 .. 1] ms	
**Neuron parameters**	**E**	**I**
V_θ – spike emission threshold	20 mV	20 mV
V_r – reset potential	16 mV	13 mV
τ – membrane time constant	15 ms	10 ms
τ_{ref} – absolute refractory period	2 ms	2ms
**Synaptic STP parameters**	**Values**	
U – probability of calcium-binding	0.2	
τ_F – time constant of calcium unbinding	1500 ms	
τ_D – time constant of transmitter refill	200 ms	
**Synaptic LPA parameters**	**Values**	
M – probability of LPA binding	0.6	
τ_L – time constant of LPA unbinding	30 s	
ΔU – change in U during LPA binding	0.1	
**Baseline input parameters**	**E**	**I**
$\mu\tau$ – mean external current	23.1 mV	21 mV
$\sigma\sqrt{\tau}$ – standard deviation of external current	1 mV	1 mV
**Working memory input parameters**	**Values**	
T_{init} – duration before cue stimulation	10 s	
T_{cue} – duration of cue stimulation	350 ms	
T_{total} – total duration of the simulation	95 s	
A_{cue} – contrast factor of selective cue	1.15	

Table C. Firing rate network model.

**Model summary**	
**Populations**	One: excitatory selective population $E_{\mathrm{selective}}$ (mean-field representation)
**Connectivity**	$E_{\mathrm{selective}}$ is connected to itself via an STP synapse with connection strength J
**Neuron model**	—-
**Synapse model**	Mean-field STP synapse (with or without noise)
**Input**	Constant (negative) input I_{base} into population E
**Measurements**	Population activity E of the network population $E_{\mathrm{selective}}$, state variables of the mean-field STP synapse

<table>
<tr><th colspan="4">Populations</th></tr>
<tr><th>Name</th><th>Type</th><th>Dynamics</th><th>Function</th></tr>
<tr><td>$E_{\text{selective}}$</td><td>Mean-field representation of an excitatory neural population, characterized by its population activity E</td><td>Population activity dynamics:
$$\tau \frac{dE}{dt}(t) = -E + g(JyxE + I_{\text{base}}) + \xi_E,$$
where τ is the time constant of the population activity dynamics and y and x are the synaptic STP parameters for presynaptic calcium binding and neurotransmitter availability respectively. $\xi_E(t)$ is a Gaussian white-noise perturbation with mean zero and autocorrelation $\langle \xi_E(t)\xi_E(t')\rangle = \sigma_E^2 \delta(t-t')$. The nonlinear gain function g is given by:
$$g(z) = \alpha \log(1 + \exp(z/\alpha)).$$</td><td>Excitatory population that is selective to the WM cue</td></tr>
</table>

<table>
<tr><th colspan="2">Synapse models</th></tr>
<tr><th>Name</th><th>Dynamics</th></tr>
<tr><td>STP synapse</td><td>Synaptic dynamics of the presynaptic calcium binding variable y and the neurotransmitter availability x:
$$\tau_F \frac{dy}{dt} = U - y + U(1-y)E + \xi_y(t)$$
$$\tau_D \frac{dx}{dt} = 1 - x - yxE + \xi_x(t),$$
with parameters U (rate of presynaptic calcium binding), τ_F (time constant of calcium unbinding) and τ_D (time constant of neurotransmitter replenishment) and where $\xi_{x,y}(t)$ are Gaussian white-noise perturbations with mean zero and autocorrelation $\langle \xi_{x,y}(t)\xi_{x,y}(t')\rangle = \sigma_{x,y}^2 \delta(t-t')$.</td></tr>
<tr><td>LPA signaling</td><td>Synaptic dynamics for LPA signaling, with bound LPA rate ℓ:
$$U = U_b + \Delta U \ell$$
$$\tau_L \frac{d\ell}{dt} = -\ell + M(1-\ell)yxE$$
with parameters U_b (baseline rate of presynaptic calcium binding), ΔU (maximal increase of U with LPA binding), M (probability of LPA binding) and τ_L (time constant of LPA unbinding).</td></tr>
</table>

<table>
<tr><th colspan="2">Input</th></tr>
<tr><td>Baseline input</td><td>Constant input E_{base}, takes negative values to reflect inputs from inhibitory populations not included in the model</td></tr>
<tr><td>Cue stimulation</td><td>After an initial period of baseline input stimulation T_{init}, the population E receives an elevated input current $E_{\text{cue}} = A_{\text{cue}} \times E_{\text{base}}$ for a short period T_{cue}, which represents a stimulation of the network with a working memory cue. Afterwards, the external input is reduced to baseline level E_{base} for the remainder of the simulation.</td></tr>
</table>

<table>
<tr><th colspan="2">Measurements</th></tr>
<tr><th>Type</th><th>Method</th></tr>
<tr><td>Population activity E</td><td></td></tr>
<tr><td>Duration of population spike regime</td><td>Analogous to duration measurement in the spiking network</td></tr>
<tr><td>Working memory regime</td><td>Classification of delay activity as one of four dynamical regimes:
• silent – no population spikes during the delay, i.e. no synchronized spike with amplitude larger than 60 percent of the cue response
• transient – at least one population spike during delay and time between last observed spike and the end of the simulation larger than the maximum interspike interval (ISI)
• persistent – regular population spiking until the end of the simulation
• self-evoked – population spikes already before the cue presentation or extended periods without population spikes during the delay</td></tr>
</table>

Table D. Standard parameters of the firing rate network, if not stated otherwise.

**General simulation parameters**	**Values**
dt – simulation time step	0.1 ms
**Population parameters**	**Values**
τ – time constant of population activity in population E	13 ms
J – synaptic efficacy of E-E synapse	10
α – exponent of nonlinear gain function g	1.5
σ_E – strength of white-noise perturbation on E	$0 \ldots 0.1$
**Synaptic parameters**	**Values**
U – probability of calcium-binding	0.2
τ_F – time constant of calcium unbinding	1500 ms
τ_D – time constant of transmitter refill	200 ms
σ_x – strength of white-noise perturbation on x	$0 \ldots 0.05$
σ_y – strength of white-noise perturbation on y	$0 \ldots 0.05$
**Synaptic LPA signaling parameters**	**Values**
M – rate of LPA binding	0.4
ΔU – change in U during LPA binding	0.4
U_b – time constant of transmitter refill	200 ms
τ_L – time constant of LPA unbinding	5 sec
**Baseline input parameters**	**E**
I_{base} – mean external current	-3
**Working memory input parameters**	**Values**
T_{init} – duration before cue stimulation	5 s
T_{cue} – duration of cue stimulation	500 ms
I_{cue} – external current during cue presentation	5

Table E. Parameter ranges for the synaptic STP model and experimental references on which they are based.

Reference	τ_D [ms]	τ_F [ms]	U
Tsodyks et al. (1998) [7] (Facilitation)	130	530	0.03
Tsodyks et al. (1998) [7] (Depression)	800	0	0.5
Wang et al. (2006) [8] (Facilitation-dominant, n=63)	194 ± 18	507 ± 37	0.28 ± 0.02
Wang et al. (2006) [8] (Depression-dominant, n=52)	671 ± 17	17 ± 5	0.25 ± 0.02
Wang et al. (2006) [8] (Pseudolinear, n=24)	329 ± 53	326 ± 66	0.29 ± 0.03
Saviane and Silver (2006) [9]	12 ($<$ 17)	12	0.5 ± 0.2
Mongillo et al. (2008) [1]	200	1500	0.3
Cortes et al. (2013) [10]	200	1500	0.3
Zenke et al. (2015) [11]	200	600	0.2
De Pittà and Brunel (2016) [12]	10 - 2000	500 - 2000	0.09 (Facilitation), 0.6 - 0.9 (Depression)
Mi et al. (2017) [2]	200 - 600	1000 - 3000	0.2

Supplementary Figures

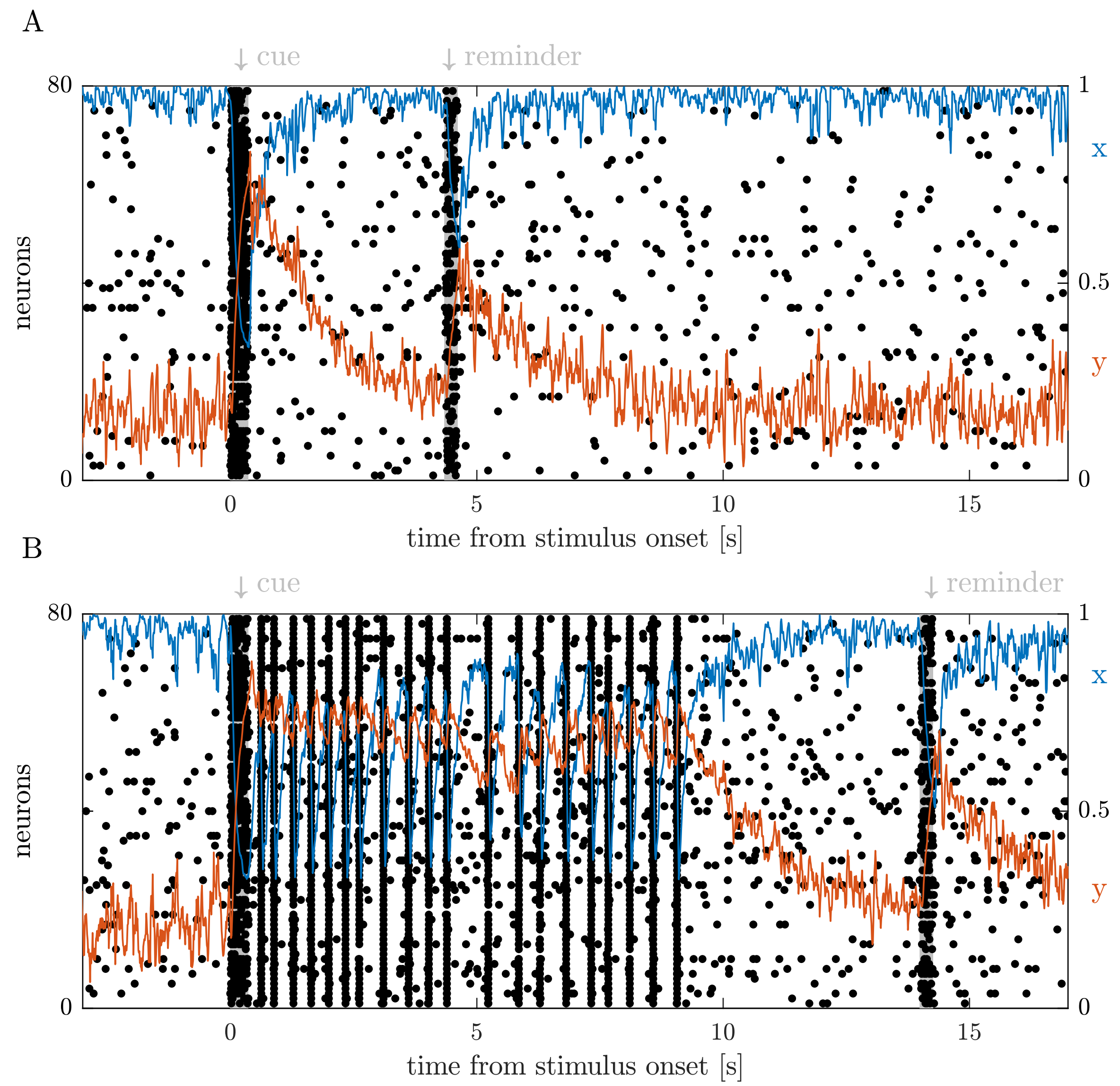

Fig A. Reminder stimulation. A weak reminder stimulus is presented to a spiking network in the silent regime (A) and after transient WM activity (B). In both cases, the reminder re-evokes the working memory in form of a single population spike. The raster plot shows 80 randomly selected neurons from the selective excitatory population. The synaptic variables for neurotransmitter availability (x) and calcium-binding (y) are depicted in blue and red, respectively. Parameters: (A) $\tau_L = 5$ s, $\mu_E \tau_E = 22.95$ mV, interval between end of cue and begin of reminder stimulation $T_{\text{reminder}} = 4$ s; (B) $\tau_L = 5$ s, $\mu_E \tau_E = 23.165$ mV,$T_{\text{reminder}} =$ 5 s.

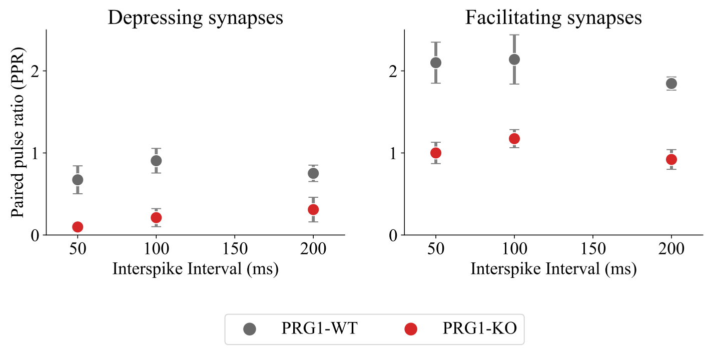

(A) Overview of paired-pulse ratio (PPR) results of electrophysiological recordings [13, 14] in mice in depressing [13] and facilitating [14] synapses under healthy (black) and pathological (red) PRG1-KO condition.

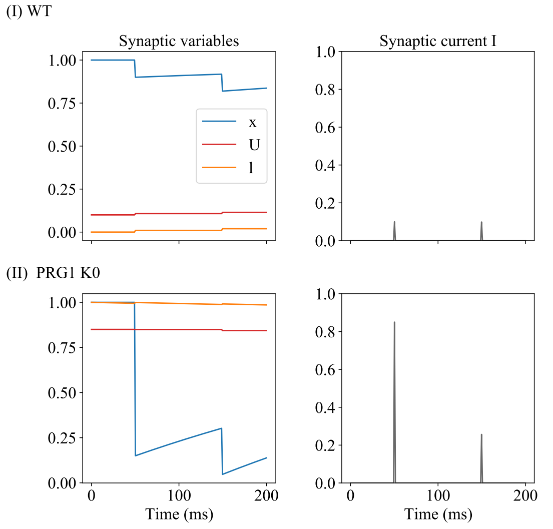

(B) STP+LPA synapse behavior for depressing synapses ($\tau_D = 500ms$, $\Delta U = 0.75$, Eqs. 1, 2, 3 and 4,5) with incoming spikes at $t = 50$ms and 150ms (ISI=100ms).

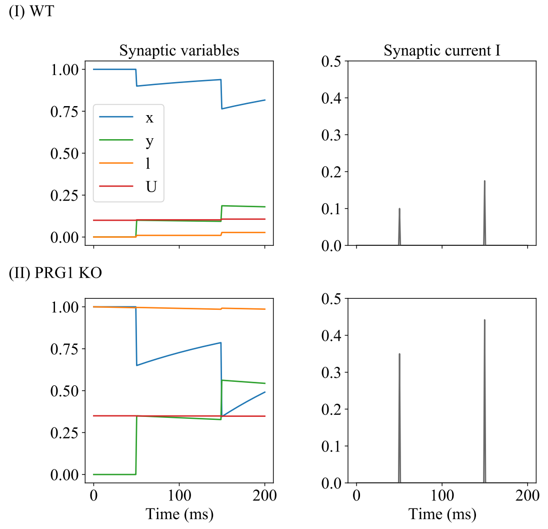

(C) STP+LPA synapse behavior for facilitating synapses ($\tau_D = 200ms$, $\tau_F = 1500ms$, $\Delta U = 0.25$, Eqs. 1, 2, 3 and 4, 5) with incoming spikes at $t = 50$ms and 150ms (ISI=100ms).

Fig B. Illustration of the effect of increased synaptic LPA on the Paired-Pulse-Ratio. Experimental recordings (A) and two stereotypical simulations for depressing and facilitating synapses (B, C). The LPA concentration in the synaptic cleft ℓ regulates the presynaptic release probability U, therefore leading to a larger release of neurotransmitters in the first spike, and a relative reduction in the second spike. PRG1-KO pathologically increases LPA signaling. Therefore here, $M = 0.1$ for WT and $M = 1$ for PRG1-KO, and the initial LPA concentration ℓ is 0 and 1, respectively. We use $\tau_L = 9sec$ and $U_b = 0.1$.

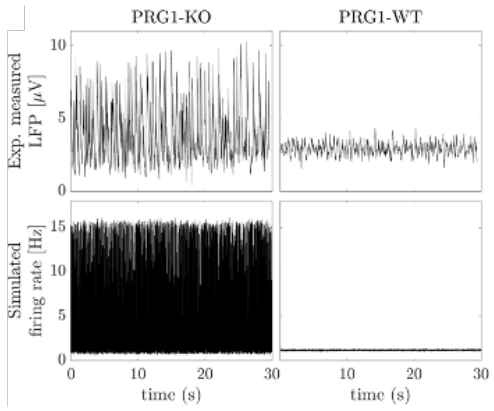

Fig C. Networks with LPA-signaling reproduce qualitative differences between PRG1-KO compared to WT mice. Spontaneous network activity under healthy conditions (experiments: WT mice, model: low values of the PRG1 parameter M) is much lower than under pathological conditions (experiments: PRG1-KO mice, model: high values of M).

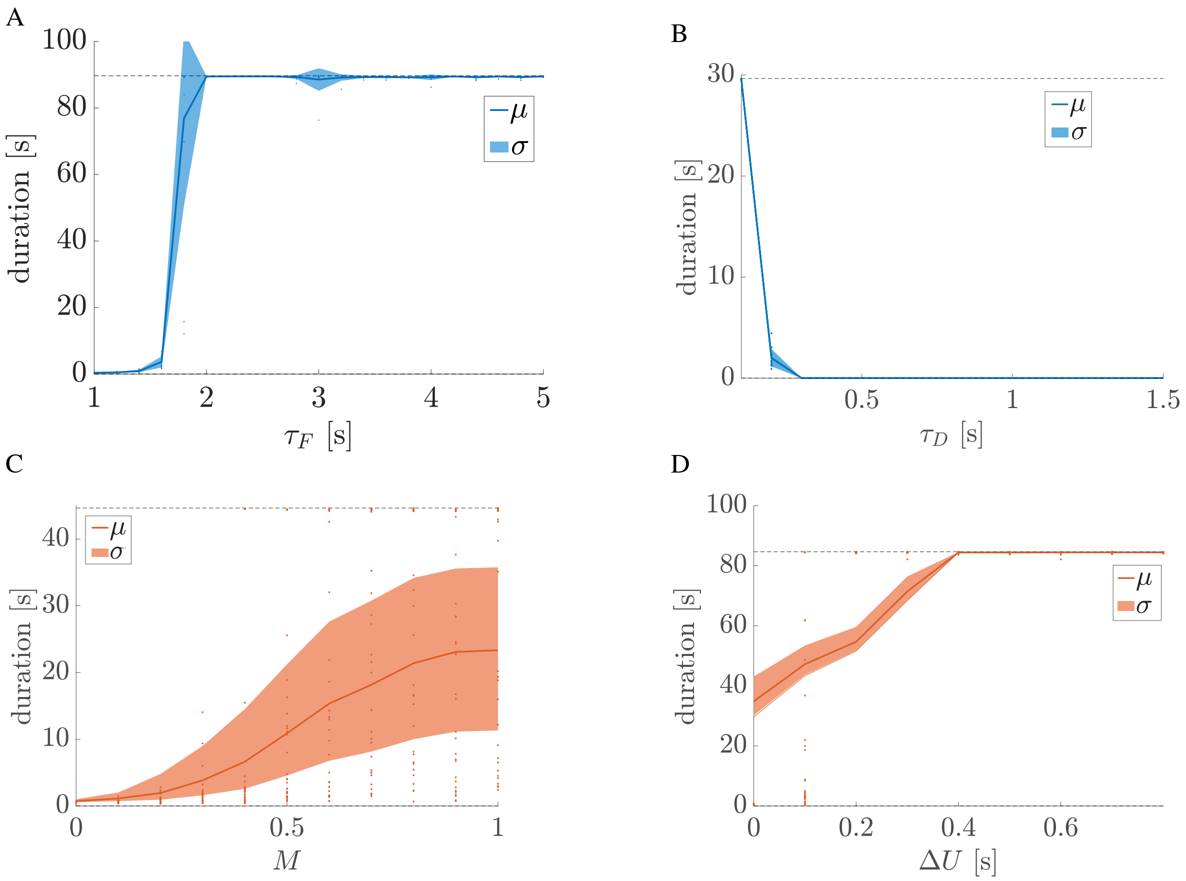

Fig D. Modulation of transient WM duration by synaptic parameters in a spiking network. Mean (thick blue line), median (thin blue line) and standard deviation (blue shading) of transient WM durations for varying values of (A) the time constant of calcium unbinding τ_F, (B) the time constant of neurotransmitter replenishment, (C) the LPA binding rate M, and (D) the maximal LPA-mediated change in presynaptic calcium-binding rate ΔU. Simulations are performed with (C, D) and without (A, B) LPA signaling. The statistics are computed across 15 (A, B) and 30 (C, D) simulations for each parameter value.

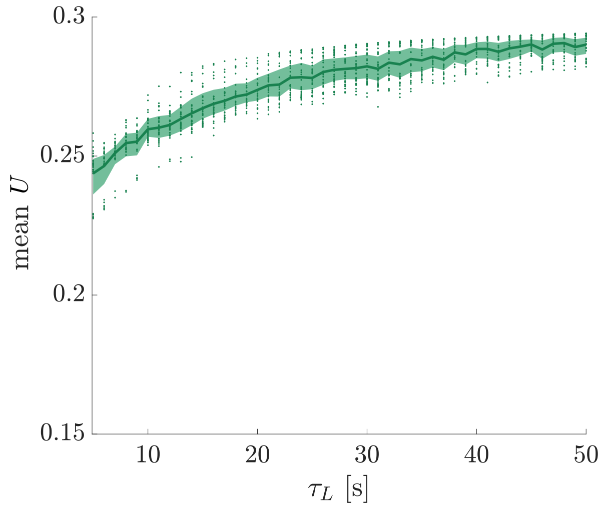

Fig E. Time constant of LPA unbinding τ_L modulates transient WM duration via the presynaptic calcium-binding rate U in a spiking network. The median (green line) of the average U value across the population spike activity increases with τ_L. The standard deviation (green shading) remains roughly constant. Each green dot represents the mean value of U during the population spike activity of a single simulation. The plot shows 30 samples per value of τ_L.

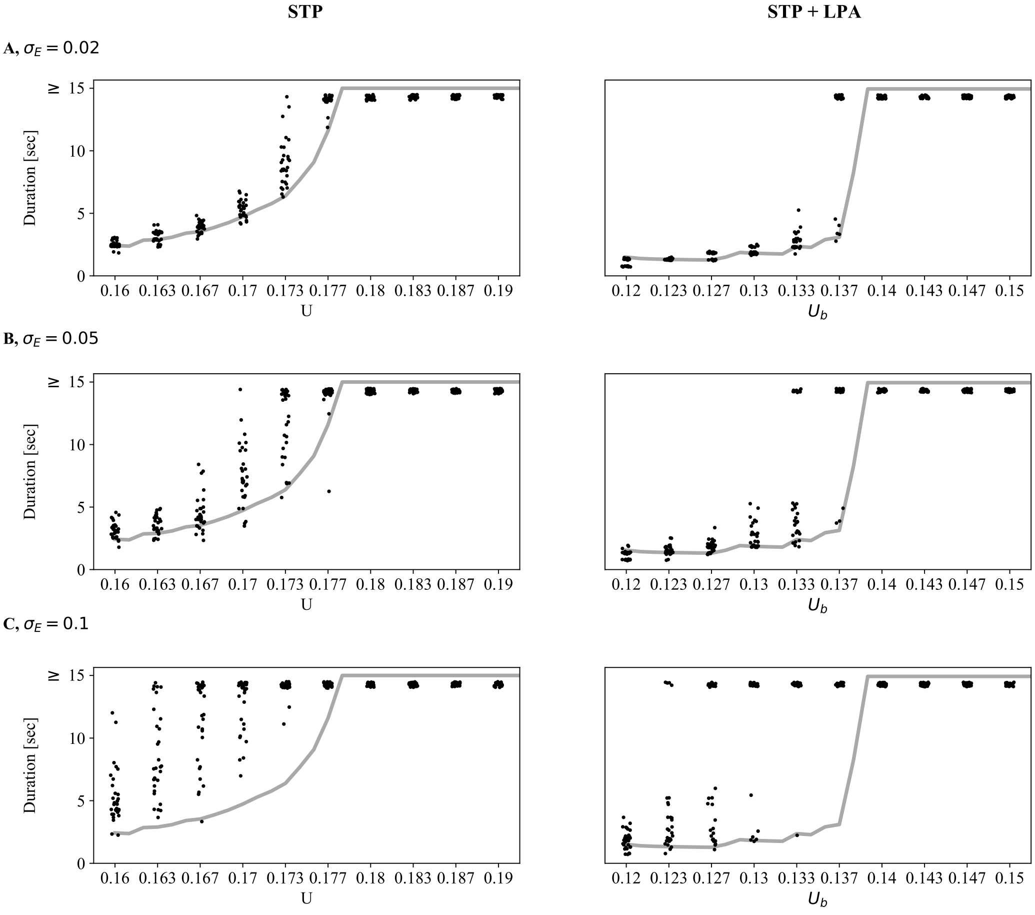

Fig F. Transition from the silent to the persistent regime as shown in Figs 4F (STP rate model) and 6F (STP+LPA rate model) of the main manuscript, for the respective STP or STP+LPA firing rate model with varying noise levels σ_E in the population activity. STP-only and STP+LPA synaptic signaling are shown in left and right column, respectively. Here, $\sigma_{x,y} = 0$. We show 30 simulation results for each parameter value U, U_b. Grey lines represent the case with zero noise.

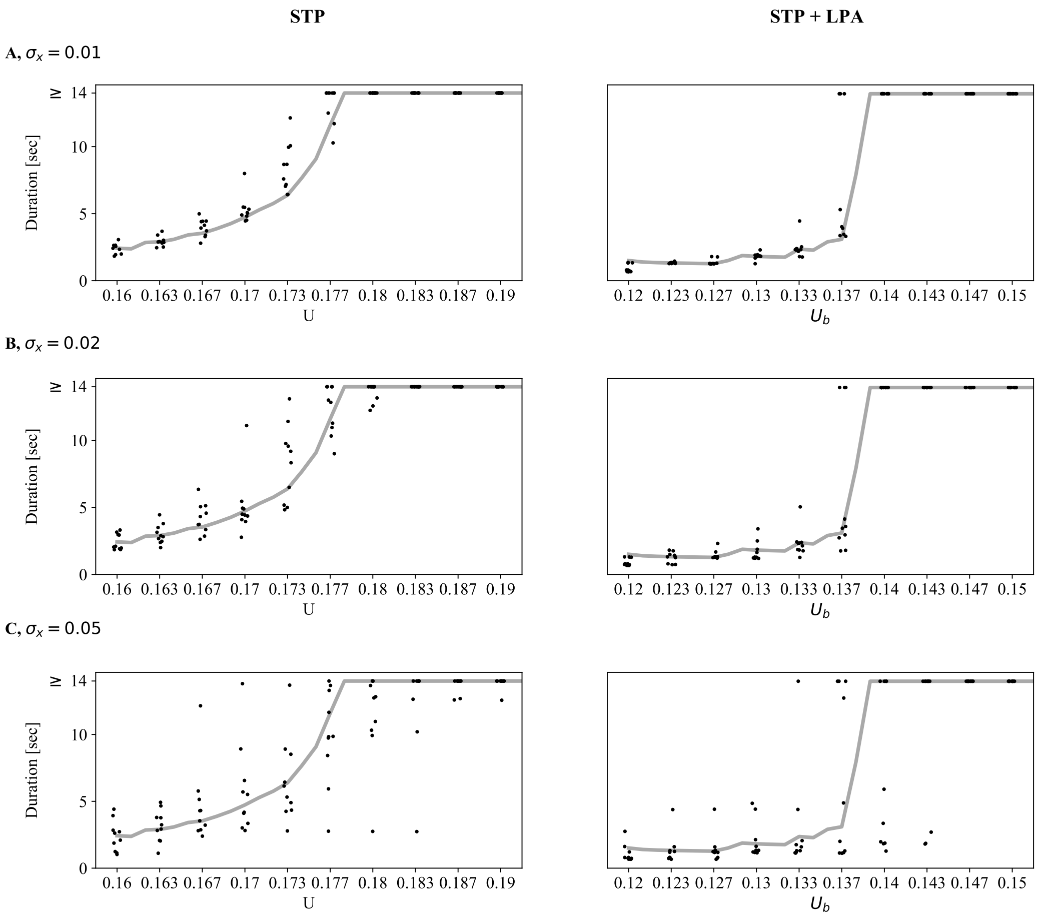

Fig G. As in Fig F in S1 Text, but varying σ_x, and setting $\sigma_{E,y} = 0$.

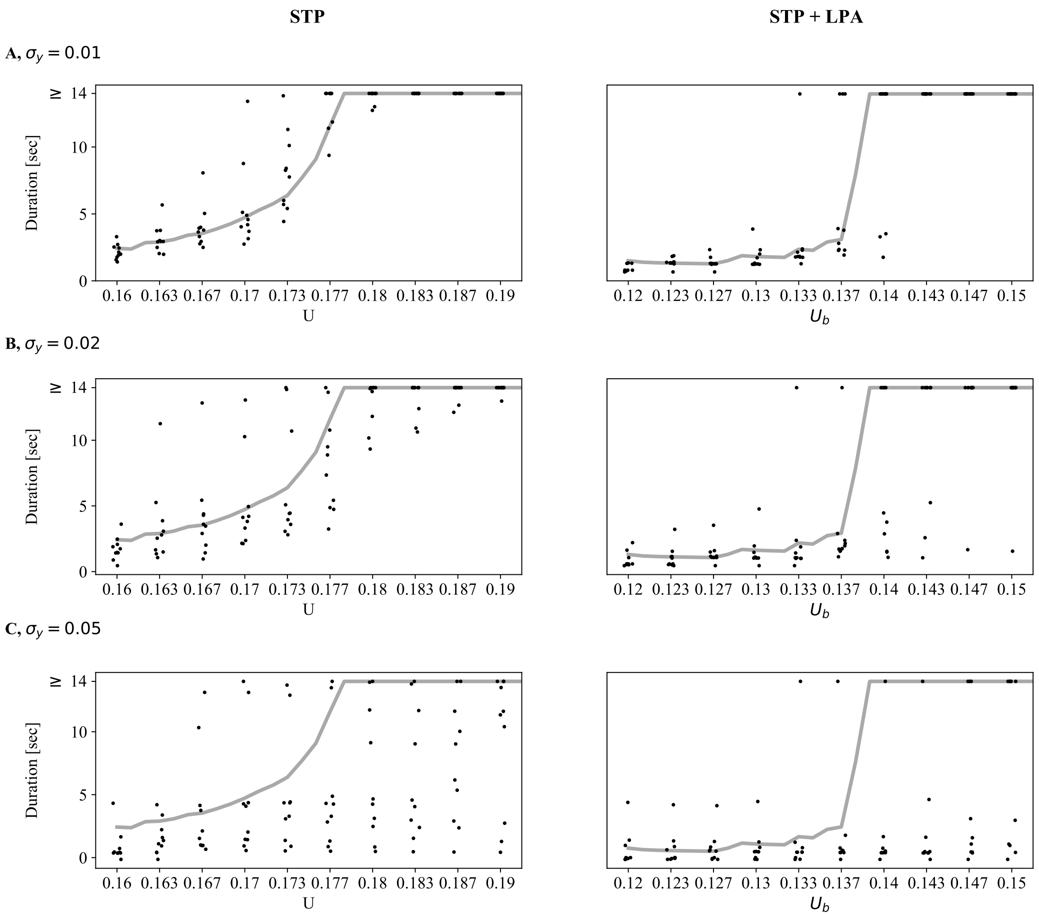

Fig H. As in Fig F in S1 Text, but varying σ_y, and setting $\sigma_{E,x} = 0$.

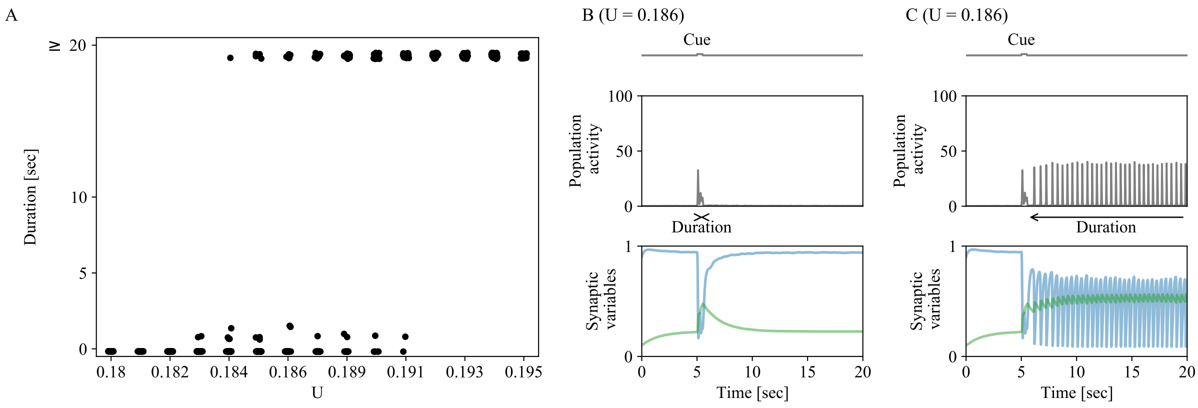

Fig I. Transition from the silent to the persistent regime for an STP rate model with weak cue stimulus ($I_{cue} = -1$). A: Duration of transient WM activity after cue stimulus for 30 simulations for each value of U. B,C: Visualization of population activity and synaptic state for two simulations at $U = 0.186$, (style as in Fig 4B and 4C). The network response is either silent (with maximal two population spikes) or persistent activity that is not interrupted within the simulation time.

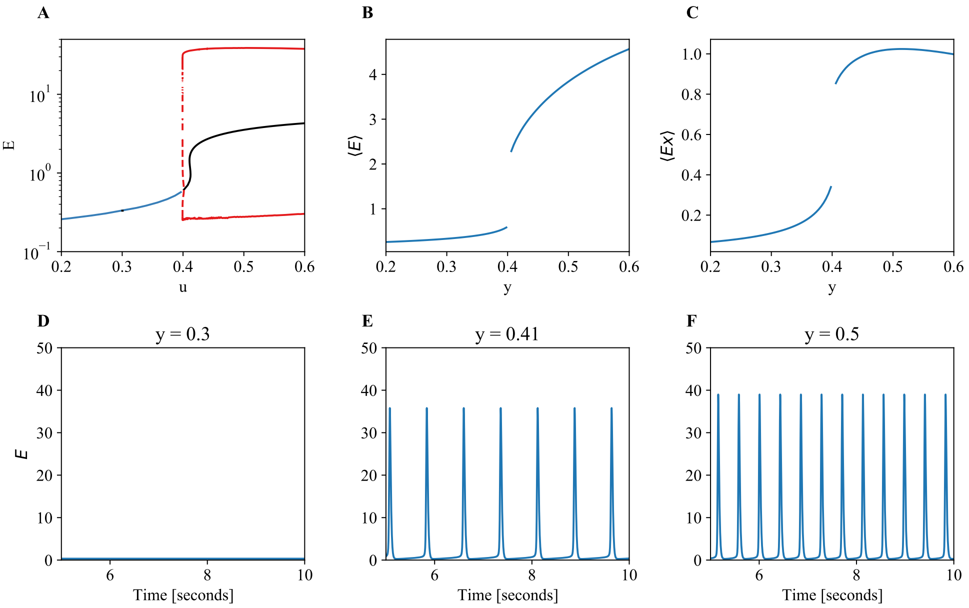

(A) **A**: Bifurcation diagram of the STP rate model with stable and unstable fixed points depicted in blue and black solid lines. Minimum and maximum value of stable limit cycles are depicted as red solid and unstable limit cycle as red dashed lines. **B, C**: Mean values for E and Ex. **D-E**: Examples for the population activity of simulations for stable fixed point and periodic oscillatory behavior for different values of y.

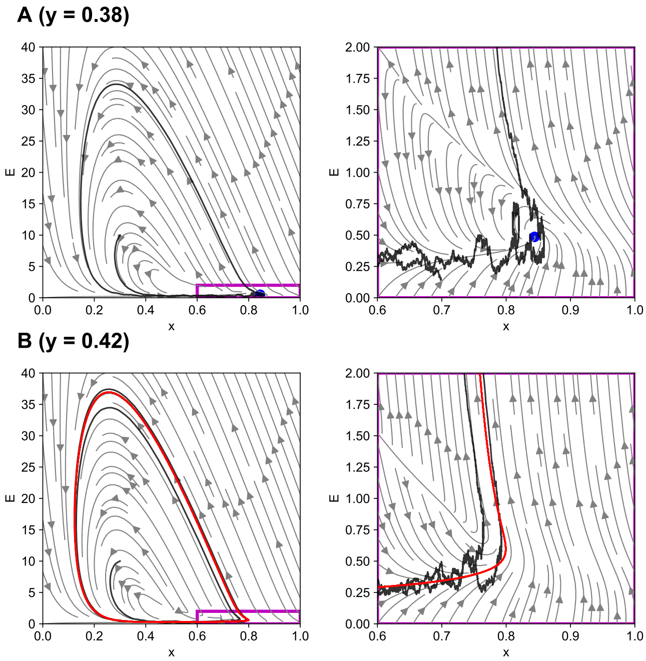

(B) Black trajectories depict simulations for the reduced system with noise ($\sigma_E = 0.05$). Grey arrows represent the direction field. The blue dot and red path represent the stable fixed point and the stable limit cycle, respectively. The panels on the right show the direction field and path within the rectangle highlighted in magenta in the left panels. (A) shows the system for $y = 0.38$, where there is only a stable fixed point. With noise, the system can exit the basin of attraction of the fixed point and is kicked back to perform a trajectory close to the "ghost" of the limit cycle [15]. (B) shows the system for $y = 0.42$, where a stable limit cycle exists. Noise only perturbs the trajectory slightly, mainly in the slow region of low E-values.

Fig J. Dynamics of the reduced STP firing rate model for fixed vesicle release probability y (remaining parameters: see Table D in S1 Text). In this scenario, the population activity E interacts with synaptic depression x only (see Ref. [16] for a similar analysis of the interaction of membrane potential and synaptic depression in the context of epileptic spiking).

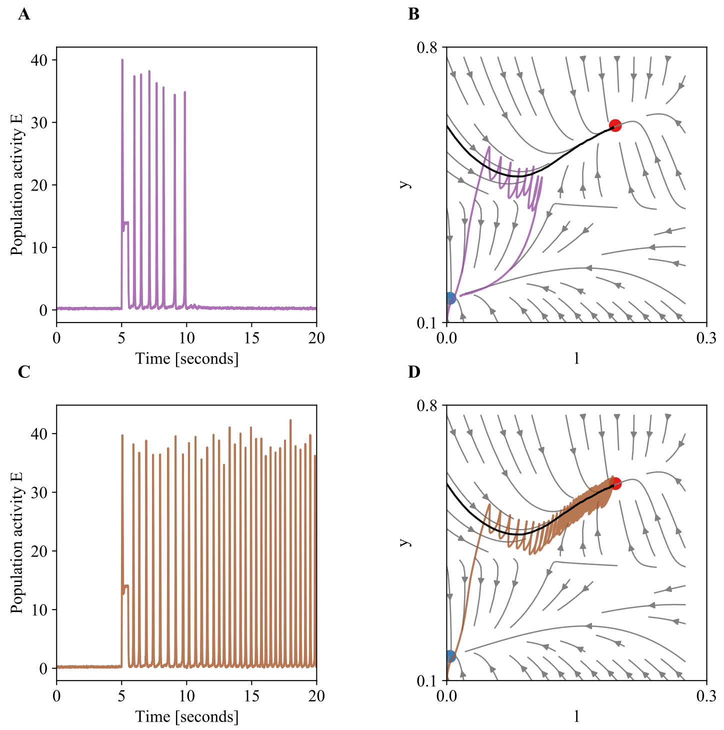

Fig K. Two realizations for the full STP+LPA firing rate equations with noise (see Tables C and D in S1 Text), depicted by colored line for the same parameters, as used in Fig 6H and 6I, respectively ($U = 0.133$). In **B,D** we project the simulation onto the direction field of Eq. 11, for which a separation of timescales is assumed. The blue and red dots represent the stable fixed point and the stable limit cycle, respectively.

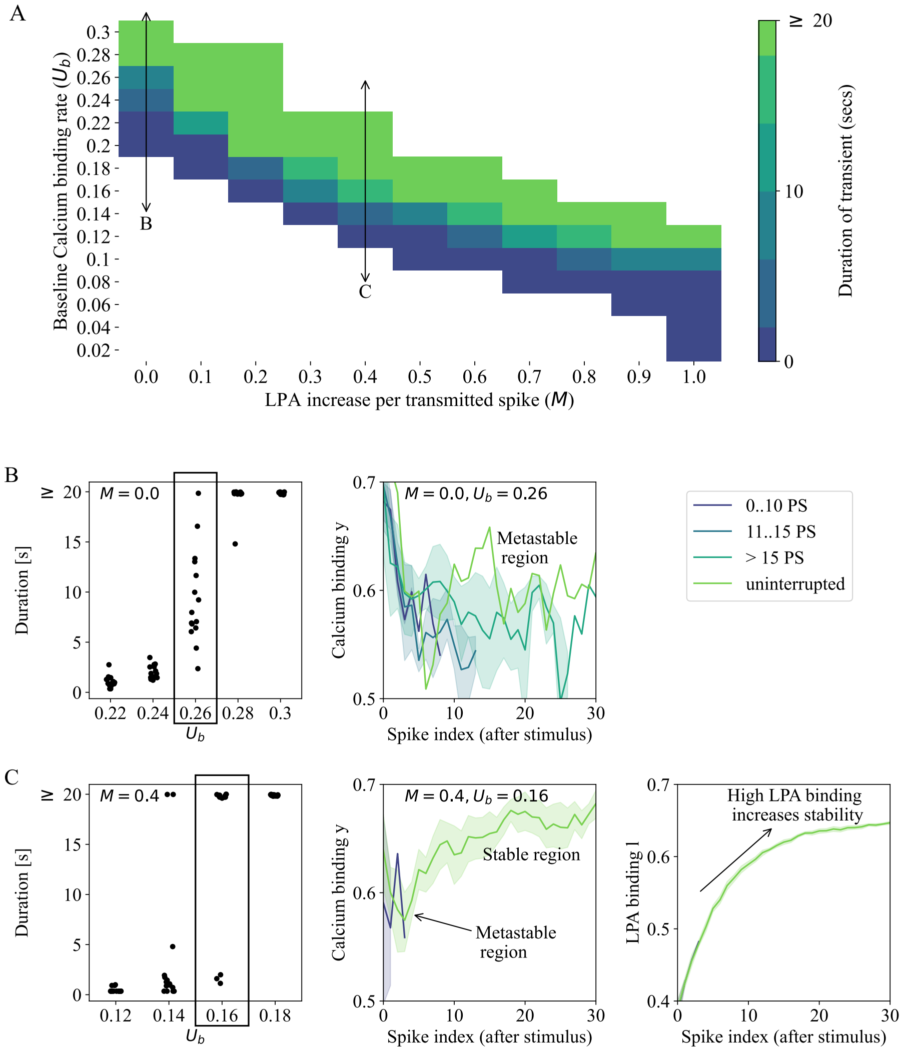

Fig L. Transient population activity is determined by synaptic dynamics in spiking networks. We compute 15 simulations for each pair of parameters (U_b, M). (A) Mean WM duration as a function of the strength of the LPA-signaling pathway M and the baseline of calcium-binding U_b. As M increases, the transition from silent (blue) to persistent (green) WM activity moves to lower values of U_b. The transition along U_b is sharp (within ≈ 0.04), whereas it is smoother along the M-axis (within $\approx 0.1 \ldots 0.3$). (B) Left: Distribution of durations for $M = 0.0$ (no LPA-signaling). Right: Average evolution of synaptic variable y after the cue stimulus, prior to each population spike, for the simulations at the transition ($U_b = 0.26$), highlighted by the black box in the left panel. The population spikes are ordered by index (see also Figs M-O in S1 Text) and the simulations are categorized by number of population spikes (PS) of their transient activities. y stabilizes after approx. 5 seconds and the system remains in a metastable regime until it is disrupted. (C) As in (B), but $M = 0.4$ (with LPA signaling) and with an additional panel showing the evolution of the LPA binding ℓ. At the transition ($U_b = 0.16$), the duration of WM activity is either short (< 5 sec) or uninterrupted within the simulation time. Immediately after stimulus presentation the system is in a metastable regime, and leads to termination in some cases. After 4-5 seconds, LPA binding ℓ increases, which increases calcium-binding y, therefore stabilizing the system. Here, $\Delta U = 0.25$, $\tau_L = 9$ sec.

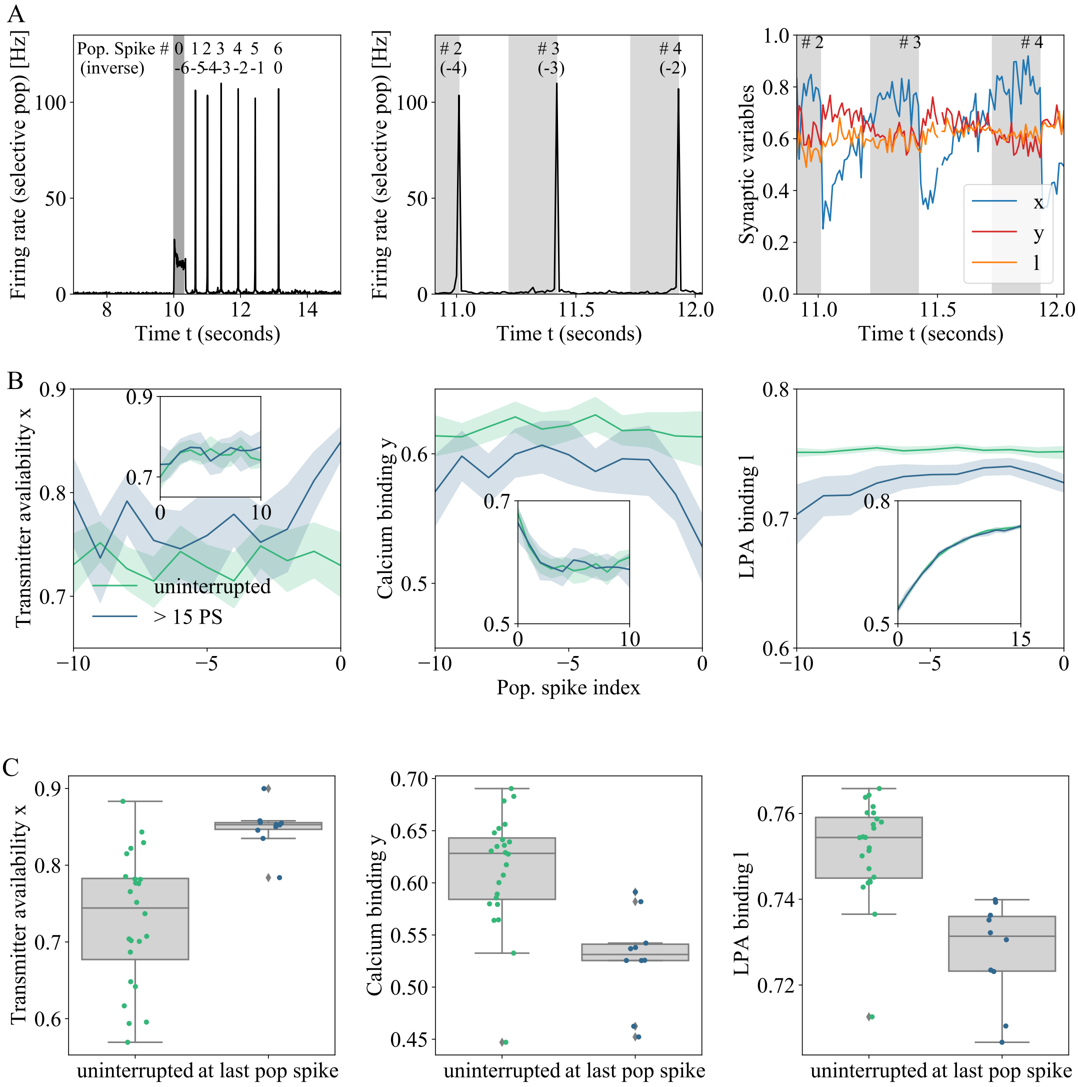

Fig M. Analysis of the termination behavior of WM activity for spiking network simulations. Here, the effect of LPA binding on the baseline increase of calcium-binding is weak ($\Delta U = 0.1$). A: Firing rate of the selective population for one realization with six population spikes after cue stimulus. Left: Full time course, dark grey bar indicates the stimulation with the WM cue. Center/Right: Population activity (center) and synaptic variables (right) in a section around three population spikes. Here, the light grey areas show the time interval of $200ms$ preceding each population spike. B: Synaptic variables averaged over a time interval of 200 ms preceding each population spike (PS), as the point of termination of the oscillatory population activity is approached. We compare two groups: Simulations which did not terminate ('uninterrupted') within 20 seconds, and simulations which did terminate after at least 15 population spikes. Solid lines represent the mean, shades the standard deviation for each group. The x-axis denotes the index of the population spike before termination (0 is the last population spike). Transmitter availability x tends to increase, and calcium-binding y tends to decrease up to two PS prior to termination. LPA binding is slightly lower for simulations that terminate. Insets show the evolution of the synaptic variables after presentation of the stimulus. C: Direct comparison of synaptic variables prior to the last pop spike in simulations which terminate, and simulations which are only interrupted due to finite computation time of 20 seconds.

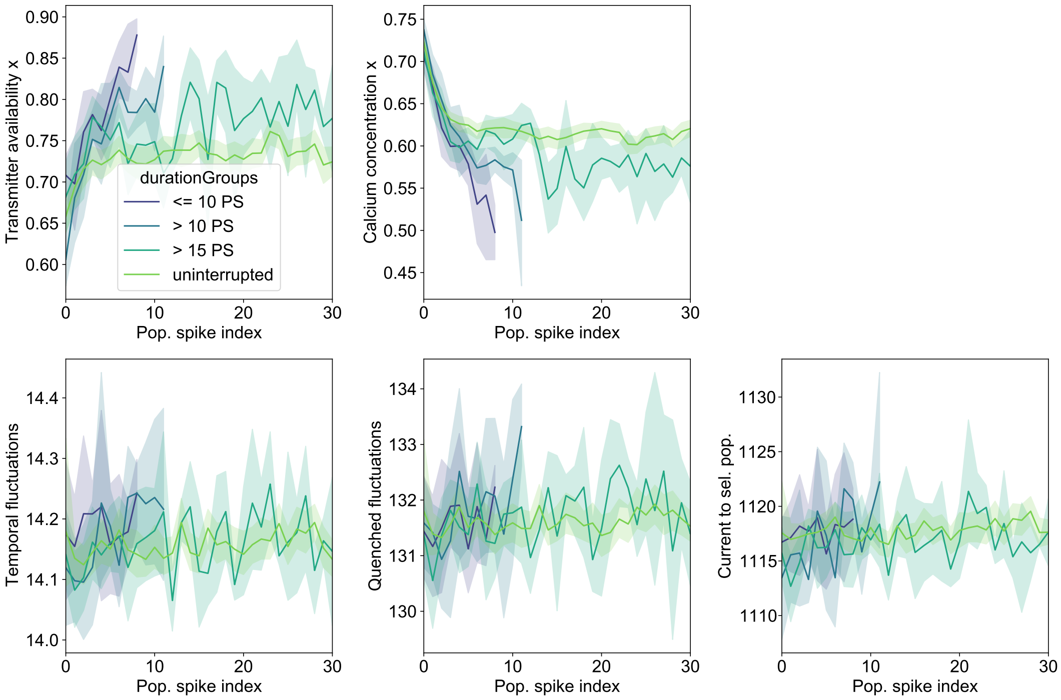

Fig N. Evolution of synaptic variables ordered by population spikes for a working memory trial without LPA signaling ($M = 0$, $U = 0.27$) with other parameters as in Fig M in S1 Text). The plots show the behaviour after stimulus cue, where 0 represents the first population spike of the simulation. Here, we show results for 75 trials, for 5 different realizations of network connectivity (25 trials per configuration, distribution of durations are shown in Fig P in S1 Text). Values are averages of the 200ms prior to each population spike (solid line: mean, surrounding shade: standard deviation). Figures show presynaptic transmitter availability x, Calcium concentration y in the first row. The second row shows values for the currents to selective excitatory population: Their temporal fluctuations $[\mathrm{mV}/\sqrt{\mathrm{sec}}]$, quenched fluctuations $[\mathrm{mV}/\mathrm{sec}]$ as well as their mean values $[\mathrm{mV}]$.

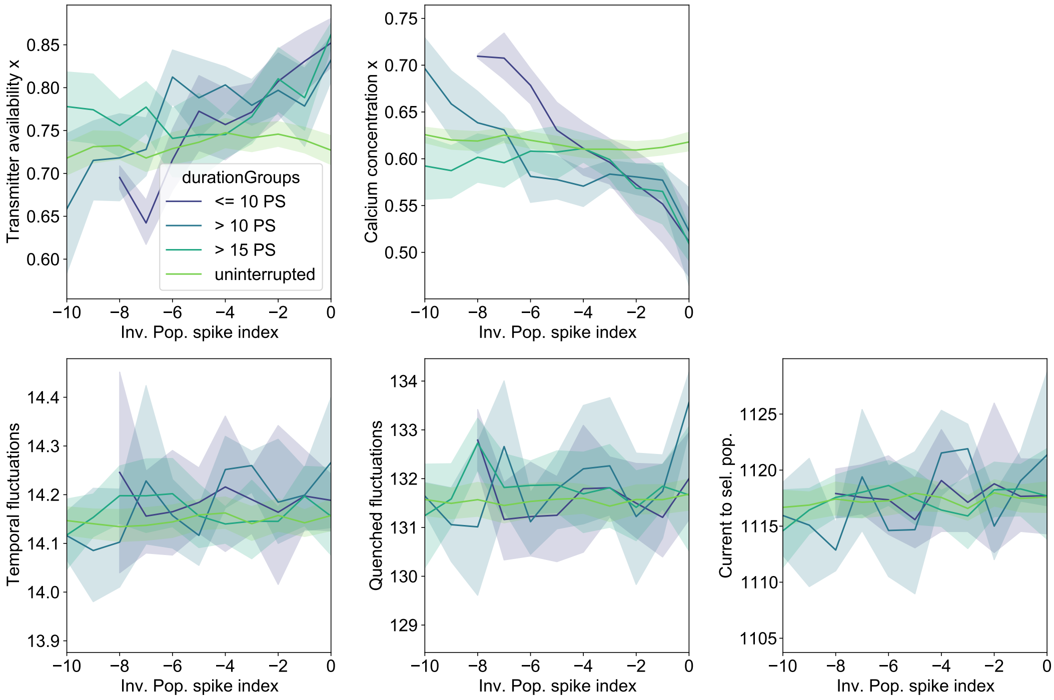

Fig O. Same as in the previous Fig N in S1 Text but showing the behavior prior to termination of the WM representation, where 0 represents the last population spike of the simulation. Again, solid lines and shadings represent the mean and standard deviation for each group.

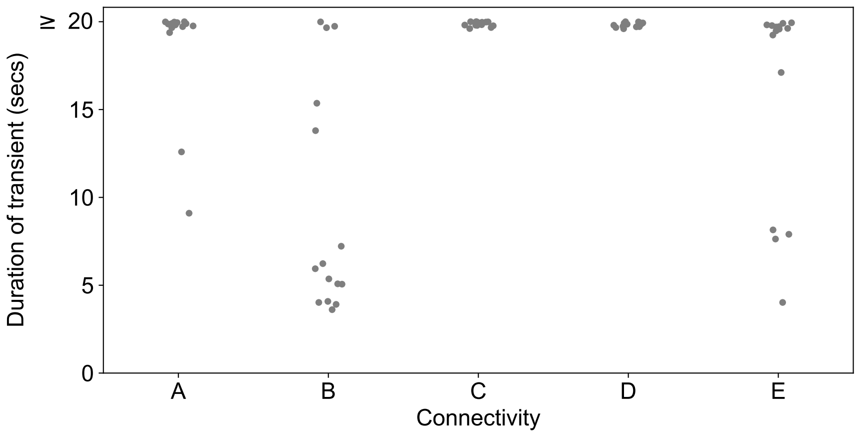

Fig P. Distribution of durations of WM representations for a working memory trial without LPA signaling ($U = 0.27$) for five different realizations of network connectivity.

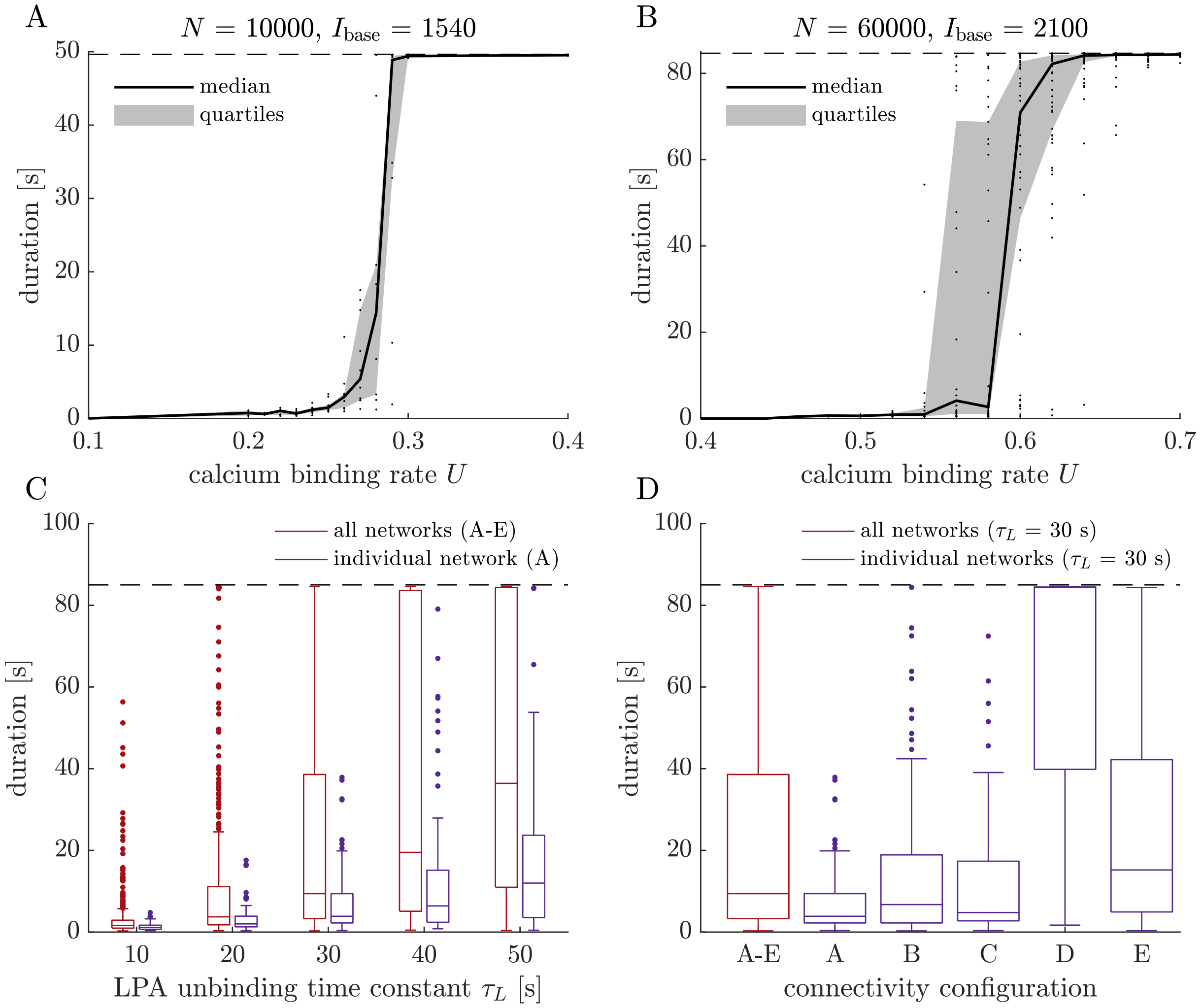

Fig Q. The duration of transient active WM is robust to changes in network size but sensitive to network wiring. (A, B) Networks with different sizes support transient WM. Here, we show transitions for populations with N=10 000 and N=60 000 total number of neurons, consistent with cortical column sizes in different animals [17]). The ratio of selective, unselective and inhibitory population sizes are the same as in Table B in S1 Text). The transient regime appears in different ranges of the baseline input current $I_{base} = \mu_E$ (in mV/sec) and the presynaptic calcium-binding rate U in the two networks. (C, D) The wiring pattern of a network influences the statistics of the transient WM durations. Here, the seed determining the random connectivity of each network realization is fixed for network configurations A-E. For each of these five networks with different wiring, 100 realizations are shown either separately for each network (purple boxplots) or together (red boxplot). Network wiring accounts for a large part of the variability of WM durations (see D), but does not affect their trend to increase with the LPA unbinding time constant τ_L (see C).

References

[1] Mongillo G, Barak O, Tsodyks M. Synaptic Theory of Working Memory. Science. 2008;319(5869):1543–1546.

[2] Mi Y, Katkov M, Tsodyks M. Synaptic Correlates of Working Memory Capacity. Neuron. 2017;93(2):323–330.

[3] Nordlie E, Gewaltig MO, Plesser HE. Towards reproducible descriptions of neuronal network models. PLoS Comput Biol. 2009;5(8):e1000456.

[4] Berridge MJ, Bootman MD, Roderick HL. Calcium signalling: dynamics, homeostasis and remodelling. Nat Rev Mol Cell Biol. 2003;4(7):517–529.

[5] Kolata S, Wu J, Light K, Schachner M, Matzel LD. Impaired working memory duration but normal learning abilities found in mice that are conditionally deficient in the close homolog of L1. J Neurosci. 2008;28(50):13505–13510.

[6] Thalman C, Horta G, Qiao L, Endle H, Tegeder I, Cheng H, et al. Synaptic phospholipids as a new target for cortical hyperexcitability and E/I balance in psychiatric disorders. Nat Mol Psychiatry. 2018;23(8):1699–1710.

[7] Tsodyks M, Pawelzik K, Markram H. Neural Networks with Dynamic Synapses. Neural Comput. 1998;10(4):821–835.

[8] Wang Y, Markram H, Goodman PH, Berger TK, Ma J, Goldman-Rakic PS. Heterogeneity in the pyramidal network of the medial prefrontal cortex. Nature Neuroscience. 2006;9:534–542.

[9] Saviane C, Silver RA. Fast vesicle reloading and a large pool sustain high bandwidth transmission at a central synapse. Nature. 2006;439:983–987.

[10] Cortes JM, Desroches M, Rodrigues S, Veltz R, Muñoz MA, Sejnowski TJ. Short-term synaptic plasticity in the deterministic Tsodyks-Markram model leads to unpredictable network dynamics. Proc Natl Acad Sci U S A. 2013;110(41):16610–16615.

[11] Zenke F, Agnes EJ, , Gerstner W. Diverse synaptic plasticity mechanisms orchestrated to form and retrieve memories in spiking neural networks. Nature Communications. 2015;6:6922.

[12] De Pitta M, Brunel N. Modulation of Synaptic Plasticity by Glutamatergic Gliotransmission: A Modeling Study. Neural Plasticity. 2016;2016:7607924.

[13] Unichenko P, Kirischuk S, Yang JW, Baumgart J, Roskoden T, Schneider P, et al. Plasticity-related gene 1 affects mouse barrel cortex function via strengthening of glutamatergic thalamocortical transmission. Cereb Cortex. 2016;26(7):3260–3272.

[14] Vogt J, Kirischuk S, Unichenko P, Schlüter L, Pelosi A, Endle H, et al. Synaptic Phospholipid Signaling Modulates Axon Outgrowth via Glutamate-dependent Ca2+-mediated Molecular Pathways. Cereb Cortex. 2017;27(1):131–145.

[15] Medeiros ES, Caldas IL, Baptista MS, Feudel U. Trapping phenomenon attenuates the consequences of tipping points for limit cycles. Scientific reports. 2017;7(1):1–11.

[16] Bart E, Bao S, Holcman D. Modeling the spontaneous activity of the auditory cortex. Journal of computational neuroscience. 2005;19(3):357–378.

[17] Markram H, Muller E, Ramaswamy S, Reimann MW, Abdellah M, Sanchez CA, et al. Reconstruction and Simulation of Neocortical Microcircuitry. Cell. 2015;163(2):456–492.
