# Supplementary material for: Modulation of working memory duration by synaptic and astrocytic mechanisms
Source: PLoS Comput Biol. 2022 Oct 3;18(10):e1010543. doi: 10.1371/journal.pcbi.1010543 (PMC9560596; doi:10.1371/journal.pcbi.1010543)
Supplement: Attachment — Submitted filename: PCOMPBIOL-D-21-02324_R0.pdf [file pcbi.1010543.s002.pdf]

PCOMPBIOL-D-21-0234
Title: Synaptic and astrocytic modulation of working memory durations
Authors: Becker, Sophia and Nold, Andreas and Tchumatchenko, Tatjana

Overview

Becker et al.'s study is an elegant attempt to understand the biophysical mechanisms underpinning working memory (WM) duration. They present original arguments on how the combination of different noise sources could have opposite effects on WM encoding, either stabilizing or destabilizing it. Furthermore, they provide analytical and computational arguments that pinpoint astrocytic regulation of basal synaptic release as a critical factor in regulating the duration of WM neuronal correlates. Their modeling work extends the network model originally introduced by Mongillo and collaborators in Science 2009 to account for different noise sources and astrocytic regulation of presynaptic release by astrocytically-synthetized lysophosphatidic acid (LPA).

While the paper bears excellent potential for novelty and introduces exciting ideas, the current manuscript appears to be detrimentally disorganized. It is often hard to understand what model between spiking and rate model one is looking at. The exposition of the results could be substantially improved and currently suffer from figures only marginally explained. The questions raised in the introduction do not match the arguments presented in the discussion. Overall the manuscript should be self-contained, while it is not. Hence I am advising for major reshaping.

Suggestions for reshaping.

Title. How about: "*Synaptic and astrocytic mechanisms of modulation of working memory duration*"?

Introduction. I would shrink the initial part L22-L24 in favor of better elaborating L35-L53. Specifically, emphasis should be put on the mechanisms of birth and death of WM neural correlates.

I would also introduce before L54 a proper paragraph on astrocytes, arguably discussing the notion that up to 80% of cortical synapses could be tripartite (e.g., Katshuri and Lichtman's works from 2015 and 2021). Then proceed with your L54-68, however, reversing the order of exposition and justifying the peculiar choice of LPA synthesis rather than more common gliotransmitter release pathways (e.g., Araque et al., Neuron 2016).

Results. I would advise rearranging your analysis into three parts. The first part is the study of noise without LPA synthesis. Second is the introduction of LPA without noise (or at fixed noise). Third is analyzing the interplay of the two.

What I found misleading in the exposition of the results is your choice to leave out any raster plot that could exemplify network behavior. Example simulations to accompany your figures like those in Figure S3 could be very helpful to get an idea at a glance of your arguments (besides providing a conceptual liaison with previous work). Figure S2 should also be re-integrated into the main text.

I am not a fan of your definition of "window of opportunity" nor "decision window" since they send to some undisclosed Maxwell's Daemon who could arguably take on the opportunity.

Discussion. Your discussion should first address the three questions you pose in your introduction and then elaborate on more speculatory arguments (e.g., energetic ones). In any case, the way your current discussion is phrased appears more of an extension of your result analysis, with individual paragraphs picking single figures. Instead, I would opt to generalize your arguments beyond explicit reference to individual figures that should be reduced to essential.

Some more specific comments.

- L26: Add a reference to Lundqvist and Miller's works showing WM mechanisms by oscillations.
- L44: Drop ref. 30.
- L53: What is metastable for you? There is no clear explanation of this state based on the bifurcation arguments that you previously provided as background.
- You should also explicitly mention the existence of a limit cycle (arguably around L46 when you mention the homoclinic bifurcation). This part could also be expanded to briefly mention the possibility of exploiting bifurcation ghosts (e.g., ref. 49), as you later mention in your analysis.
- L55: the actual 2-equation model was presented entirely by Misha in his contribution to Les Houches Monography in 2005. Ref. 21 still uses the 3-equation version. I would either replace the reference or add the one from 2005.
- L58: Drop refs. 35–37 and append Araque et al. (Neuron 2014).
- L78: Activity-silent. I would also append references to Mongillo et al. (PRL 2012) and Hansel and Mato (JN 2013).
- L84: Your model of modulation of synaptic release by astrocytic signaling leverage the TM model and the model previously introduced by De Pitta and co-workers and generalized in De Pitta (Comput. Gliosci. 2019; Ch 10) and De Pitta (Encyclopedia of Comput Neurosci. 2020). However, I am missing the explanation of why LPA synthesis depends on the postsynaptic current (the scheme in Figure 1a does not show it).
- L78-L107. This section seems more to explain your model and should be separated from the results. I would advise you to re-integrate Methods and Models before the Results and provide concise explanations in a discursive fashion about your different models (synaptic/spiking w/ astrocytic signaling + network models). The way models are presented in the end and in a lumped table-like fashion does not help understand your work.
- L140: Why infinity? What induces it? (I assume persistent activity emerges, but then clarify).
- L160–175: Address in two separate paragraphs: first the stabilizing effect of noise, then the destabilizing one. Also, include essential equations for the different noise sources (and what biophysically motivates them).
- L176: What nonlinearity? Please fully develop this crucial argument and expand your analysis.
- L205+: It should be a new paragraph (if not even a new section).
- L210-214: Unclear. Please rephrase and make an effort to provide an interpretation in terms of biophysical mechanisms.
- L230-245: This paragraph stormed me by surprise. You suddenly mention pathology and epilepsy. It seems off-topic.
- L279-286: Split. Address points 1 (decision window) and 2 (energetic benefits) in two separate paragraphs.
- L304: You never really introduced the concept of a tripartite synapse. It is barely mentioned in the introduction, but I am afraid you might be too confident to believe that everyone knows what you are talking about.

Equations and notation.

- For historical reasons, I would stick to the original notation for the facilitating variable, *u* rather than *y*. And \gamma rather than \ell. Moreover, I would adopt lower case letters for time-dependent quantities and upper case letters for constants. Thus U --> u_0 for example.
- Eq. 2-3: why not to define r(t_k) = u(t_k^+)x(t_k^-)?
- Eq. 5: It must be \DeltaU>U_b in your model to reproduce your results if I am not mistaken. On the other hand, since U must be in [0,1], the choice \DeltaU is subjected to constraints that should be stated.
- There is some inconsistency in notation: e.g., sometimes \ell and other times l (cp. Figures in the main text with Fig. S1 legends). Panels are sometimes labeled by lower case letters and upper case ones. Please be consistent in your style choices.

Figures.

- Figure 1 is somehow misleading. You show the synaptic model, but you provide population activity plots (when no neurons are shown in the scheme). I assume that you are using the rate model here? Otherwise, I cannot justify, unless by averaging, how you can show lumped synaptic variables.
- Figure 2. A: Are those population rates? B and C: What are the barely visible gray data? D: Replot as a 2D heatmap. It would help if you showed the cue on the plots in A, as you do elsewhere.
- Figure 3. Put some color panels together: B should be orange simulations + orange phase space trajectories; C should be the same for red ones. Do you need to plot in 3D?
- Figure 4. Arguably split A and B into two figures. Here again, it is not clear why you show me a synaptic scheme, but the simulations appear to be in the network. I cannot follow your reasoning and cannot easily match the bifurcation diagrams in A/B5 with the simulations in A/6.
- Figure 5. A: Why upward arrows for B and C? B and C: take out the scheme from the plots: keep your plots as clean as possible. I do not understand the yellow dots. Left panels for Calcium-binding and LPA binding are unreadable to me. You do not need to comment on your plots (e.g., “High LPA...”) since you should address the full explanation of your figures in the Results.
- Figure 6. It is a nice idea but only marginally discussed. Please elaborate in a fully-developed paragraph.
- Figure S1. A: Error bars are barely visible. B: What is the pre-synaptic stimulus? You should arrange these figures vertically, in four columns of three panels each (pre | syn vars | post).
- Figure S4. Compared with analogous plots in Figure 2, you are showing STD rather than quartiles. And the median does have little use. Please choose one style and keep it consistent throughout all your figures.
- Figures S6–8: Gray lines are barely visible. Along with Figures S9 and S15, reconsider plotting all data points by box plots or violin plots along with extrema as well.
- Figures S12--S14: what are the shades? SDs? SEMs? Please clarify. Improve contrast.
