# Supplementary material for: Modulation of working memory duration by synaptic and astrocytic mechanisms
Source: PLoS Comput Biol. 2022 Oct 3;18(10):e1010543. doi: 10.1371/journal.pcbi.1010543 (PMC9560596; doi:10.1371/journal.pcbi.1010543)
Supplement: Attachment — Submitted filename: Reply.pdf [file pcbi.1010543.s003.pdf]

Synaptic and astrocytic modulation of working memory durations

Sophia Becker, Andreas Nold, Tatjana Tchumatchenko

Thank you for the opportunity to revise our manuscript following the many helpful comments of the referees. We proceed by addressing each comment point-by-point and highlight the original comment in grey. For the sake of clarity, additions-modifications in both the manuscript and SI, including new equations and references, have been highlighted in red while new-modified figures have been enclosed in a red frame. If not stated otherwise, all page, line, equation and figure numbers refer to the updated version of the manuscript.

Reviewer 1

The paper presents a consideration of a biologically motivated slow astrocytic regulation of presynaptic release probability in a working memory model. Using firing rate and spiking network simulations, the authors show how the duration and stability of working memory representations are altered by astrocytic regulation and by noise. It is a topic of interest to the researchers in the related areas. For the readers, however, a number of points need clarifying and certain statements require further justification.

We are grateful for the many helpful comments which helped us improve the presentation of our results. We explain below how we addressed the comments of the referee and how we modified the manuscript to accommodate them.

My detailed comments are as follows: (1) Abstract could be more specific. Please emphasize in the abstract Background, Methodology/Principal Findings, and Conclusions/Significance.

Thank you for the helpful suggestion. We rewrote our abstract and now provide additional details on the background, methodology, findings and conclusions of our study. For example, we now explain our methodology in more detail:

> *'Here, we introduce a slow, transmission-dependent astrocytic regulation of the presynaptic release probability in a synaptic attractor model of WM. We compare and analyze simulations of a simple WM protocol in firing rate and spiking networks with and without astrocytic regulation, and underpin our observations with analyses of the phase space dynamics in the rate network.'*

We also state our main findings more explicitly, e.g.

> *'We find that the duration and stability of working memory representations are altered by astrocytic signaling and by noise. We show that astrocytic signaling can control the mean duration of WM representations. Moreover, if the astrocytic regulation is strong, the slow ramping of astrocytic modulatory effects at the presynapse introduces a 'window of vulnerability', during which WM representations are easily disruptable by noise before being stabilized. We identify two mechanisms through which noise from different sources in the network can either stabilize or destabilize WM representations.'*

Lastly, we elaborate in more detail on the conclusions from our work, e.g.

> *'Our findings suggest that (i) astrocytic regulation is crucial in determining the duration of WM representations in synaptic attractor models of WM, and (ii) that astrocytic signaling could facilitate different mechanisms for volitional top-down control of WM representations and their duration.'*

(2) My other more general comment is that the text needs some work. It's quite difficult to read through long sentences. Especially in sections 'Transient WM activity emerges from silent and persistent network dynamics due to noise' and 'LPA signaling modulates the distribution of transient durations at the transition from the silent to the persistent state'.

Thank you for the suggestion to enhance text clarity. We have carefully proof-read and edited the entire manuscript and shortened or split long sentences, with particular focus on the two results sections mentioned by the referee. In the editing process, we were supported by a professional language editing software and the feedback of colleagues.

(3) Why was such a number of neurons chosen? What determines the ratio of the number of neurons in selective and unselective populations? How will the behavior of the network change if inhibitory neurons are excluded?

Thank you for these helpful questions. We now discuss these numbers in the model description in section 'Spiking network: model and parameters' of the SI: *'For better consistency and comparability with previous work, the number of neurons in each population, including the ratio of selective to unselective excitatory neurons, was chosen as in the studies by Mongillo et al. [5] and Mi et al. [57]. Note that the inhibitory population is needed to balance the activity of the excitatory population – the interplay of excitatory and inhibitory activity assures that the overall network activity stays in a balanced regime and neither goes to zero nor explodes.'*

What topology of the astrocytic network and the structure of the organization of neuron-astrocytic interaction at the network level is assumed in the model?

Thank you for this insightful question. We do not consider the topology of astrocyte-to-astrocyte and astrocyte-to-neuron interactions. Instead, we assume that astrocytes influence synaptic signalling locally through LPA synthesis. We assume that the parameters of this interaction are homogeneous across the network.

We address this point with three changes to the manuscript. First, we restructured Fig. 1 to emphasize the model design. Second, we refined the extended model description in section 'Spiking network: model and parameters' of the SI to present all relevant details on the astrocyte parameters and their synaptic coupling. We now write:

> *'Note that our current PRG spiking network does not model astrocyte-to-astrocyte interactions but assumes that an astrocyte is synapse specific and regulates the activity of a single synapse. Because we assume all synapses of a sub-population to be statistically identical (except for the connection weights which can differ) we can define an average synaptic and astrocytic state.'*

Third, we now discuss the topology of astrocyte-astrocyte and neuron-astrocyte interactions in the discussion, where we compare our model to other relevant publications in the field:

> *'Several extensions to our model can be made that lie outside the scope of this study. Importantly, we currently do not include astrocyte-to-astrocyte interactions in our model. Instead, we make a 'mean-field' assumption regarding the interactions between neurons and astrocytes, i.e. we assume that the astrocytic modulation of presynaptic STP dynamics happens independently in each synapse and in a statistically identical fashion throughout the network. Recent work by Gordleeva et al. [39] addresses the effects of astrocytic coupling during astrocyte-modulated WM formation. They model astrocyte-to-astrocyte interactions as diffusive coupling of astrocytic intracellular IP3 and calcium concentrations in a lattice of partly*

overlapping astrocytes. Similar to presynaptic LPA binding in our model, their astrocytic calcium and IP3 dynamics are dependent on synaptic activity. In contrast to our study, however, they do not include short-term synaptic plasticity in their model'.

(4) How the loading of an item into WM was designed? It is necessary to clarify whether the cue stimulation differed from stimulation parameters during training.

Thank you for this question. In brief, we do not explicitly model training, but assume a spiking network that already includes a selective and an unselective population (see Fig. 1 of the revised manuscript). For the rate network, we only model the selective population. To address this comment, we have expanded the corresponding paragraphs in the Models and Methods section of the new manuscript:

- Spiking networks:

  'All neurons in the network receive an excitatory external baseline input current with constant, population-specific mean and Gaussian white noise. We model the presentation of a WM item to the network as a temporary increase ($T_{cue} = 100ms$) of the mean external input into the excitatory selective population. After the presentation of the WM cue, the mean excitatory input into the excitatory selective population is reduced back to its baseline value. The network response after the offset of the WM cue (i.e. during the 'delay period') is what we refer to as WM representation. Note that we do not explicitly simulate the learning and long-term plasticity through which a selective sub-population is formed but assume that this has taken place before the onset of our WM simulations. The reason for this setup is that in this study, we exclusively look at the effects of astrocytic modulation on short-term synaptic plasticity.' and *'The STP+LPA spiking network [...] uses the same [...] WM protocol as the STP spiking network'*.

- Rate networks:

  'As in the [...] spiking network, a WM stimulus is presented as a temporarily increase in the mean of the external baseline input for the duration of the memory cue. However, while the external input reflects the combined inputs from inhibitory and excitatory presynaptic populations, the cue presentation increases only the excitatory (not the inhibitory) part of the input.'.

Moreover, we have clarified the model description in the caption of Fig. 1: *'During the WM protocol, all neurons receive an excitatory baseline input with Gaussian white noise. When the WM cue is presented, the neurons of the excitatory selective population receive an additional external input I_{cue}.'*
Lastly, we have included the following sentence in the introduction to distinguish our model from Hebbian STP models: *'In contrast to Hebbian, associative forms of synaptic STP [**?**, **?**] we consider non-associative models, i.e. in order to store information about a WM stimulus, synaptic structures to represent the stimulus in the network already need to exist'*.

How the system will behave in multi-item WM tasks?

Thank you for this interesting question. While simulations of multi-item WM tasks are outside of the scope of this work, we introduced a new discussion paragraph in which we discuss this exciting direction for future research. Specifically, we present our predictions about the system's behaviour in multi-item WM tasks and consider them in the context of relevant publications in the field:

'Another exciting direction for future research is to apply our model in a multi-item WM setting similar to the settings explored for synaptic WM with STP [5, 57]. Due to the relatively fast decay of the STP traces, multiple WM items (each encoded by a separate neuronal subpopulation) are kept in memory by sequential firing of a single population spike from each population. We can speculate that the long timescale of astrocytic modulation could allow each memory to be active for an extended time before the next WM item has to be refreshed in memory. This intuition is supported by a preprint [41] that studied multi-item WM in a network with astrocytic modulation of STP. In their model, each astrocyte controls one synaptic ensemble that is involved in encoding a WM item [41]. In contrast to our study, they do not consider transient WM activity but only persistent and silent WM representations' (see discussion of revised manuscript).

What dynamic mode of the system will correspond to free (not cued) recall?

Thank you for this intriguing question. In our current study, we consider network dynamics in which working memory representations that are induced by a cue. To allow for this, the oscillatory (limit cycle) state has to co-exist with a silent (low-activity) fixed point, and the silent fixed point needs to be stable with respect to intrinsic noise levels. It would, however, be possible to model not-cued recall in the same model by using higher levels of noise that are sufficient to disrupt not only the limit cycle but also the low-activity fixed point. We included a paragraph addressing this point in the results of the revised manuscript:

'Note that here, we only consider regimes in which the noise on any of the three variables is not strong enough to induce transient population spikes without the prior presentation of the WM cue. Higher noise levels, which can lead to spontaneous re-appearance of transient WM representations, could be a promising model of free, un-cued recall of WM items. Since we focus on the spontaneous termination of active WM representations, we exclude the corresponding high-noise regimes for the remainder of this study.'

(5) Please include in the introduction or discussion a brief review of the related model studies about neuron-astrocytes interaction in the WM formation (DOI: 10.3389/fncel.2021.631485; https://www.biorxiv.org/content/10.1101/2021.03.25.436819v1; doi.org/10.1007/s00521-022-06936-9)

Thank you for pointing out these interesting and timely references which we now cite in our revised manuscript. We discuss this and other previous work in a separate paragraph in the discussion:

'Recent work by Gordleeva et al. [39] addresses the effects of astrocytic coupling during astrocyte-modulated WM formation. They model astrocyte-to-astrocyte interactions as diffusive coupling of astrocytic intracellular IP3 and calcium concentrations in a lattice of partly overlapping astrocytes. Similar to presynaptic LPA binding in our model, their astrocytic calcium and IP3 dynamics are dependent on synaptic activity. In contrast to our study, however, they do not include short-term synaptic plasticity in their model. Another exciting direction for future research is to apply our model in a multi-item WM setting similar to the settings explored for synaptic WM with STP [5, 57]. Due to the relatively fast decay of the STP traces, multiple WM items (each encoded by a separate neuronal subpopulation) are kept in memory by sequential firing of a single population spike from each population.

We can speculate that the long timescale of astrocytic modulation could allow each memory to be active for an extended time before the next WM item has to be refreshed in memory. This intuition is supported by a preprint [41] that studied multi-item WM in a network with astrocytic modulation of STP. In their model, each astrocyte controls one synaptic ensemble that is involved in encoding a WM item [41]. In contrast to our study, they do not consider transient WM activity but only persistent and silent WM representations.'

(6) It is worth noting that the paper format needs to be carefully edited, and some language expression methods of the paper need to be appropriately adjusted and polished.

Thank you very much for this comment. We polished our language and asked colleagues to proofread the manuscript. This helped us improve the language of our paper.

Some minor points:

- *Lines 60-61 Please provide the ref for 'This is motivated by a recently described signaling pathway that involves the astrocytic and postsynaptic component, acts on each synapse individually, and introduces a slow timescale.'*

Thank you for pointing out the need for more citations in this context. We now cite the works of Trimbuch et al [42], Unichenko et al. [43] and Thalman et al. [44] that were crucial in describing the LPA signaling pathway:

'At the core of our tripartite synapse model is a recently described signaling pathway ('LPA-mechanism') that relies on the lipid messenger molecule lysophosphatidic acid (LPA), the astrocytic enzyme autotaxin (ATX), and the postsynaptic plasticity-related gene 1 (PRG1) protein [42, 43, 44].'

L143 it's not clear what the expression means 'broadly stable transition'

Thank you for the suggestion to clarify this point better. We removed the term and rephrased the respective paragraph.

L181 the correct term is an Andronov-Hopf bifurcation.

Thank you for pointing us to this typo. To improve the readability for a broad and inter-disciplinary audience, we decided to reduce the amount of technical expressions from the dynamical systems field (including names of bifurcations) in the revised manuscript. Instead, we reworked and clarified our explanations of the observed phenomena.

190 please clarify the phrase 'saddle node of the periodic solution'

Thank you for the helpful remark. We changed the description to

'bifurcation between fixed point regime (i) and bistable region (ii)'

in results section of the revised manuscript. We now write:

'The bifurcation diagram of the the underlying noise-free STP rate network (Figure 4 A-4) shows that for a sufficiently strong cue stimulus, the transients appear around the bifurcation between fixed point regime (i) and bistable region (ii) along U (highlighted in grey). In this parameter region, the cue stimulus is strong enough to induce population spikes'.

237 abbreviation 'WT' used before its introduction

Thanks for the remark, we corrected this and now specify what the abbreviation means before we use it: *'For instance, PRG1 knockout in mice (PRG1-KO) leads to a decrease of the paired-pulse-ratio (PPR) compared to wild-type (WT) mice [47] (see Figure S2).'* (Models and Methods section in the revised manuscript)

Fig.5A axis label 'secs'

Thank you for the comment. We adjusted the units in all figures of the revised manuscript.

Fig. 5B,C missing legend for yellow color. These figures are difficult for the reader to understand. Maybe they can be presented in a different way.

Thank you for the comment. In the revised manuscript, we adjusted the presentation of the respective plots (now parts of Fig. 5 and Fig. 6). We also included the original figure in the SI after removing the synapse model sketches and unnecessary color coding in the left panels of B and C.

Reviewer 2

Becker et al.'s study is an elegant attempt to understand the biophysical mechanisms underpinning working memory (WM) duration. They present original arguments on how the combination of different noise sources could have opposite effects on WM encoding, either stabilizing or destabilizing it. Furthermore, they provide analytical and computational arguments that pinpoint astrocytic regulation of basal synaptic release as a critical factor in regulating the duration of WM neuronal correlates. Their modeling work extends the network model originally introduced by Mongillo and collaborators in Science 2009 to account for different noise sources and astrocytic regulation of presynaptic release by astrocytically-synthetized lysophosphatidic acid (LPA). While the paper bears excellent potential for novelty and introduces exciting ideas, the current manuscript appears to be detrimentally disorganized.

Thank you for appreciating the potential of our work and for the numerous thoughtful comments regarding the content and the structure of our manuscript. We have restructured our manuscript, following the referee's comments. We separately address each of the referee's comments below.

It is often hard to understand what model between spiking and rate model one is looking at.

Thank you for pointing out that the description of rate vs spiking model in the figures needs to be enhanced. In the revised manuscript, we have taken several steps to make the distinction between our rate model and spiking model analysis more clear:

- We have included separate illustrations of the rate and spiking network architecture in Fig. 1C and D, respectively.
- We have revised the text in the new Models and Methods section to make the model distinction more distinct. Specifically, we provide an overview of the four models used: *'We use two models of synaptic WM: one with and one without astrocytic modulation of STP. To allow for different methods of analysis, we implement each as a rate and a spiking network model, respectively (Figure 1 C, D). For the synaptic WM model without astrocytic modulation, we re-implemented the synaptic attractor spiking and rate model introduced in the seminal work of Mongillo et. al [5]. In the following, we will refer to them as STP spiking and STP rate network, respectively. To model the influence of*

the astrocytic LPA mechanism on WM, we integrate our STP synapse model with LPA signaling as described in the previous section (Equations 1, 2, 4, 5) in the synaptic STP spiking and rate models by Mongillo et. al [5]. We will refer to them as STP+LPA spiking and rate networks, respectively. See Figure 1 C and D for illustrations and detailed description of spiking and rate network architectures. A full model description is also provided in sections 'Spiking network: model and parameters' and 'Firing rate network: model and parameters' of the SI and in Table S1, Table S3.' In the referenced SI section, we explain the models in a discursive fashion and in reference to the revised model figure (Figure 1) and their respective model equations.

- We have restructured our figures and the corresponding results sections. In particular, the new structure of our figures avoids jumping back and forth between presentation of results from STP networks with and without LPA signaling. In the new manuscript, we begin with a comprehensive presentation of the four models we consider throughout the paper (Figure 1, models and methods) and a presentation of the two main observations that we explain in the rest of the manuscript (Figure 2). We then continue by treating the transient WM regime in the STP rate model (Figure 3) and comparing it between STP rate and spiking model (Figure 4). Afterwards, we add LPA signaling and analyze the transient WM regime in PRG rate and spiking networks, first for low (Figure 5) and then for high (Figure 6) levels of astrocytic modulation. We have reduced the mixing of different models in a single figure where possible and kept the same visual separation of rate model (upper half) and spiking model (lower half) in the three figures with rate vs. spiking model comparisons (Figure 4-6).
- We have furthermore rephrased paragraphs in the results section of the main text to make the distinction between rate and spiking model more clear. For example, we now explicitly state in the beginning of each section which models will be used in the section: *'[...] we compare the network dynamics arising during the delay period of the WM protocol in rate and spiking networks without and with astrocytic signaling (STP and STP+LPA rate and spiking networks, see Models and Methods)'* or *'To identify the underlying network mechanisms, we consider STP rate networks (see Figure 1 D) with three different sources of noise: [...]'*.
  Additionally, we explicitly mention the type of model which is concerned when stating or summarizing the main findings and conclusions in each results section, e.g. *' This observation holds for both spiking (Figure 2 B, also see Figures 4 B, 6 B) and rate networks (Figures 4 A, 6 A) '*.

The exposition of the results could be substantially improved and currently suffer from figures only marginally explained.

Thank you for the helpful suggestion to improve the presentation of our results and to add more detailed explanation to our figures. In addition to an overall extensive restructuring of the results section, we have carefully edited the text to include detailed explanations of all figures. As we described in the reply to the previous and the following comments, we have taken several steps to improve the presentation of the methods, results and figures. To briefly summarize we have consolidated all model definitions in one section to avoid jumping back and forth, we have reworked figures 1 and 2 to help motivate the main finding better.

The questions raised in the introduction do not match the arguments presented in the discussion. Overall the manuscript should be self-contained, while it is not. Hence I am advising for major reshaping. Please refer to the attached document for details.

To address the referee's comment and remove this inconsistency, we have restructured and rephrased the respective sections in our manuscript. Specifically, we have revised and refined the research questions posed in the introduction: *' In particular, we answer the following three questions: (i) What role do different sources of noise have for the WM encoding, i.e. for the stability of the active WM representation? [...] (ii) How does astrocytic modulation of presynaptic STP impact the stability and duration of active WM representations? [...] (iii) Finally, what is the specific role of the slow astrocytic timescale? [...]'*
Furthermore, we now restate these questions in the introductory paragraph of the discussion before discussing our answers to each of these questions in the three subsequent paragraphs of the discussion.

Suggestions for reshaping. Title. How about: "Synaptic and astrocytic mechanisms of modulation of working memory duration"?

Thank you for the suggestion to emphasize the mechanistic aspect of the work in the title. We changed the title to 'How synaptic and astrocytic mechanisms can modulate working memory durations'.

Introduction. I would shrink the initial part L22-L24 in favor of better elaborating L35-L53. Specifically, emphasis should be put on the mechanisms of birth and death of WM neural correlates.

Thank you for this helpful suggestion. We followed the referee's suggestion and shortened the introductory paragraph about different WM correlates as follows: *'Experimental data and computational models have provided evidence and understanding of a variety of WM correlates in the brain. These range from persistent delay activity [1, 2, 3, 4, 5, 6], sequential activations of neurons in the WM population [7, 8, 9] and oscillatory WM activity [10, 11, 12, 13] to activity-silent synaptic representations [5, 14, 15, 6].'*

As suggested, we have revised our explanation of the dynamics of the synaptic attractor models in the introduction and added details on the birth and death of active WM representations. Specifically, we have modified how we discuss the dynamics around the bifurcation point: *'The phase space and bifurcation diagram for synaptic attractor models have been characterized in detail [22, 23, 24, 25]. One important property that allows them to reconcile evidence of persistent and silent WM representations is that they can exhibit bistability: in certain parameter regimes, a 'silent' state (fixed point) of low, asynchronous network activity coexists with an 'active' state (limit cycle) of repetitive, synchronized spiking of the network [24, 25, 5, 26]. This bistability region emerges through a homoclinic bifurcation, i.e. a phase transition between the 'active' state (limit cycle) and the 'silent' state (saddle point) [24, 25] for sufficiently strong external inputs (or connection strengths within the network).'* Our revised manuscript also emphasizes the links between network dynamics and their interpretation in the context of WM and provides details on the birth and death of active WM representations: *'For sufficiently strong external inputs (or connection strengths within the network), the transition between the 'active' state (limit cycle) and the 'silent' state (saddle point) exhibits a homoclinic bifurcation [24, 25]. Then, within the bistable region, one can observe spontaneous, noise-driven transitions between the silent and the active network state if the noise is sufficiently strong to destabilize both the silent and the active state [24, 25]. Alternatively, if the silent and active states are stable with respect to a given level of noise, then an increase (or decrease) in external inputs can move the network state from a silent to an active (or from an active to a silent) representation [5]. This scenario is used to model WM by interpreting the short increase of external input as a WM stimulus that induces persistent delay activity (active network state). This delay activity can then be terminated by an external off-signal*

that switches the network back into the silent state, such as a population-specific inhibitory input [6] or a global reduction of excitatory input [5]. In certain parameter regimes, we also encounter the scenario that the silent state is stable with respect to noise while the active state is meta-stable, i.e. can be terminated through a noise-induced, spontaneous transition. In this case, we can switch the system to the active state by presenting a WM cue, but do not require an off-stimulus to terminate the WM representation – instead, it will terminate naturally and spontaneously by switching back to the silent state due to noise. So far, however, it is unclear whether and how this spontaneous termination of WM can be controlled.'
We now define the meta-stable state more clearly in the introduction of the new manuscript: *'In certain parameter regimes, we also encounter the scenario that the silent state is stable with respect to noise while the active state is meta-stable, i.e. can be terminated through a noise-induced, spontaneous transition.'*.

I would also introduce before L54 a proper paragraph on astrocytes, arguably discussing the notion that up to 80% of cortical synapses could be tripartite (e.g., Katshuri and Lichtman's works from 2015 and 2021).

This is a very good idea, thank you for this suggestion. In the introduction of the revised manuscript, we added a paragraph where we provide a short background on synapse-astrocyte interactions, cite Katshuri and Lichtman's work and introduce the concept of a tripartite synapse:

> *'Over the past decade, an increasing body of evidence has shown that astrocytes influence and interact with synaptic transmission at the presynaptic and postsynaptic side [27, 28, 29]. In these cases, we also speak of tripartite synapses [30]. Recent studies indicate that up to 80% of cortical synapses could be modulated in this way [31]. Astrocytic modulation of synaptic transmission can take place on timescales of several seconds [32, 33] to minutes [34, 35]. Since one astrocyte typically covers between 10^5 synapses in mice and 2×10^6 in humans [36], astrocytic modulation is potentially relevant for the coordination of network activity [37] and could influence high-level neural computations [38]. Here, we investigate how the long timescales of a specific signaling pathway at the tripartite synapse affects the stability and duration of meta-stable active WM representations, i.e. active WM representations that are induced by a cue stimulus but terminate naturally through noise, and explain the network mechanisms underlying the observed phenomena. We (i) develop a minimal tripartite synapse model of slow astrocytic modulation of the STP signaling pathway, and (ii) combine it with the synaptic WM model by Tsodyks [48] and Mongillo et al. [5] that is based on Tsodyks and Markram STP [18]. '*

Then proceed with your L54-68, however, reversing the order of exposition and justifying the peculiar choice of LPA synthesis rather than more common gliotransmitter release pathways (e.g., Araque et al., Neuron 2016).

Thank you for the suggestion to restructure the last paragraph of the introduction after introducing an additional paragraph on astrocytes. As suggested, we now start the summary of our work with the tripartite synapse model and continue with the application to the WM model afterwards. We also added an explanation of our choice to use the PRG1-LPA signaling pathway as the basis of our synaptic model:

> *'The PRG1 protein is expressed at the postsynaptic density of glutamatergic synapses [45] in several brain regions, including the hippocampus and the somatosensory*

cortex [42, 43, 44], implying that PRG1-related signaling could affect tripartite synapses across large areas of the brain. The LPA-mechanism has been implicated in a shift of the excitation-inhibition balance towards hyperexcitability [42], altered sensorimotor processing [43] and schizophrenia [46, 47, 44], making it an interesting candidate signaling mechanism for our study of astrocytic modulation of WM.'

Results. I would advise rearranging your analysis into three parts. The first part is the study of noise without LPA synthesis. Second is the introduction of LPA without noise (or at fixed noise). Third is analyzing the interplay of the two.

Thank you for your helpful advice to restructure our results section. We agree that building intuition about the mechanisms of working memory duration and the role of noise in the STP network first before showing the results with LPA could increase the accessibility.

We now begin our results section by first presenting our working hypothesis along which we develop the remaining results. Following the advice of this and other reviewers, we have consolidated all mathematical definitions in one materials and methods section and use Fig. 1 to define the model architecture at the synaptic level and on the network level (for both rate and spiking models). In Fig. 2 we now show that STP networks with and without LPA show different distributions of working memory durations: STP+LPA spiking networks have a bimodal distribution of durations (red distribution in Fig. 2) while STP spiking networks have a unimodal distribution (blue distribution in Fig. 2). As suggested, we then present the study of noise without LPA synthesis (section 'Noise can induce transient WM in silent and persistent network regimes'). There, we explain by which mechanisms noise can produce the stochastic durations in STP networks without LPA (blue histogram in Fig. 2 B). We also show how different sources of noise influence the onset and offset of the active WM representation in the system without LPA. Subsequently, we analyze the system with LPA synthesis and fixed noise. We split our analysis in two sections that reflect the two qualitatively different dynamical regimes that the system with fixed noise and LPA can exhibit ('Weak LPA signaling implements slow modulation of the mean transient WM duration via its influence on presynaptic calcium binding' and 'Strong astrocytic signaling introduces bimodal distribution of transient WM durations and 'window of vulnerability' for WM termination').

What I found misleading in the exposition of the results is your choice to leave out any raster plot that could exemplify network behavior. Example simulations to accompany your figures like those in Figure S3 could be very helpful to get an idea at a glance of your arguments (besides providing a conceptual liaison with previous work).

Thank you for the comment and the excellent suggestion to include raster plots of example simulations. We followed the referee's advice and now show raster plots of the network activity as well as traces of the synaptic variables from example simulations in STP spiking networks (Fig. 4 B-2, B-3) and PRG spiking networks (Fig. 5 B-2, B-3 and Fig. 6 B-2, B-3).

Figure S2 should also be re-integrated into the main text.

Thank you for the helpful suggestion. We have included Figure S2 in the main text as Figure 1C and added a analogous sketch of the rate model architecture (Figure 1D).

I am not a fan of your definition of 'window of opportunity' nor 'decision window' since they send to some undisclosed Maxwell's Daemon who could arguably take on the opportunity.

Thank you for pointing out the possible undesired connotations of the expression 'decision window' and 'window of opportunity'. To address the comment, we now refer to the phenomenon as 'window of vulnerability', e.g. in the results section: *'This leads to a limited "window of vulnerability" during which noise can disrupt an active memory and after which the memory is stabilized.'* The new name avoids unintended associations with the thought experiment of Maxwell's Deamon in thermodynamics and avoid giving the impression of agency in the noise-driven stochastic termination of transient WM representations.

Discussion. Your discussion should first address the three questions you pose in your introduction and then elaborate on more speculatory arguments (e.g., energetic ones).

Thank you for the helpful remark. We followed the referee's advice and restructured our discussion as suggested. We now begin with an introductory paragraph, where we summarize the main goal of our study and restate our three main research questions:

> *'We studied the termination of transient WM activity in a synaptic attractor model with and without astrocytic modulation of short-term plasticity (STP) [...]. Our findings shed light on three main aspects: (i) the role of noise and different noise sources in stabilizing or terminating WM activity; (ii) the impact of synaptic parameters and astrocytic modulation on the kind of WM representation that evolves and its stability; and (iii) the effect of the slow astrocytic timescale on the patterns of WM termination.'*

In the subsequent three paragraphs, we then discuss the answer to each question before addressing the remaining discussion topics:

- We first discuss our answer to the first question about the role of noise in stabilizing or terminating transient WM activity.
- Next, we answer the second question about the impact of synaptic parameters and astrocytic modulation on the termination of WM activity.
- Lastly, we address the third question about the specific effect of the long timescale at which astrocytic signaling influences the transient WM representations.

In any case, the way your current discussion is phrased appears more of an extension of your result analysis, with individual paragraphs picking single figures. Instead, I would opt to generalize your arguments beyond explicit reference to individual figures that should be reduced to essential.

Thank you for the helpful advice. We address the referee's comment as follows. Firstly, we removed explicit figure references from the text. Secondly, we restructured the discussion text – instead of discussing each figure one by one, we now follow a topic-oriented structure. We begin by addressing the three main research questions posed in the introduction and consider the findings from all question-related sections and figures in our answer. We then discuss the overall strengths and limitations of our model and compare it other models of WM with astrocytic modulation. In the last two paragraphs of the discussion, we connect our findings to related concepts of top-down volitional control and energy-efficient coding.

Some more specific comments. L26: Add a reference to Lundqvist and Miller's works showing WM mechanisms by oscillations.

Thank you for the comment. In the old manuscript, we had included the references to Lundqvist and Miller's work as part of the first paragraph of the introduction: *'This view has been extended by other types of neural WM correlates in the brain, including [...] 'bursting*

delay activity', where items are held in WM through short bursts with high firing that are coupled to global brain rhythms [10, 11, 12, 13]'. In the revised manuscript, we cite their work in the introduction as follows: *'These range from [...] oscillatory WM activity [10, 11, 12, 13] to [...].'*

L44: Drop ref. 30.

Thank you for the suggestion. We agree that the work Cortes et al. [26] focuses on the chaotic behaviours emerging from networks with synaptic short-term plasticity and therefore lies out of the scope of synaptic attractor models. We removed the reference in the revised manuscript.

L53: What is metastable for you? There is no clear explanation of this state based on the bifurcation arguments that you previously provided as background.

Thank you for suggesting this improvement. We refer to the active network state (with repeated, synchronized firing) in the bistability region as meta-stable, if it is less stable than the silent state with respect to a given noise level in the network. In this scenario, the active state can be spontaneously terminated due to a noise-induced switch to the silent state, whereas the silent state is stable with respect to the given noise level, i.e. does not experience spontaneous transitions to the active state. We have added an explanation of the meta-stable state based on our revised description of the network dynamics:

> *'In certain parameter regimes, we also encounter the scenario that the silent state is stable with respect to noise while the active state is meta-stable, i.e. can be terminated through a noise-induced, spontaneous transition. In this case, we can switch the system to the active state by presenting a WM cue, but do not require an off-stimulus to terminate the WM representation – instead, it will terminate naturally and spontaneously by switching back to the silent state due to noise. '*

You should also explicitly mention the existence of a limit cycle (arguably around L46 when you mention the homo-clinic bifurcation).

Thank you for this helpful suggestion. We now mention the existence of a limit cycle in L36-L41:

> *'One important property that allows them to reconcile evidence of persistent and silent WM representations is that they can exhibit bistability: in certain parameter regimes, a 'silent' state (fixed point) of low, asynchronous network activity coexists with an 'active' state (limit cycle) of repetitive, synchronized spiking of the network [24, 25, 5, 26]. For sufficiently strong external inputs (or connection strengths within the network), the transition between the 'active' state (limit cycle) and the 'silent' state (saddle point) exhibits a homoclinic bifurcation [24, 25].'*

This part could also be expanded to briefly mention the possibility of exploiting bifurcation ghosts (e.g., ref. 49), as you later mention in your analysis.

Thank you for the suggestion. In the current version of the manuscript, we decided to focus on a clear explanation of the most basic bifurcation concepts in the introduction and only introduce the concept of bifurcation ghosts (but in more detail and in reference to the revised introduction paragraph) in the results section.

L55: the actual 2-equation model was presented entirely by Misha in his contribution to Les Houches Monography in 2005. Ref. 21 still uses the 3-equation version. I would either replace the reference or add the one from 2005.

Thank you for pointing us to the original reference. We added the reference in the introduction of the revised manuscript: *'[...] We integrate our tripartite synapse model with the WM model by Tsodyks [48] and Mongillo et al. [5] [...]'*

L58: Drop refs. 35–37 and append Araque et al. (Neuron 2014).

We have now removed refs. 35-37 in the new paragraph on astrocytic modulation of synaptic transmission and added the proposed reference to Araque et al. [27]: *'Over the past decade, an increasing body of evidence has shown that astrocytes influence and interact with synaptic transmission at the presynaptic and postsynaptic side [27, 28, 29].'*.

L78: Activity-silent. I would also append references to Mongillo et al. (PRL 2012) and Hansel and Mato (JN 2013).

Thank you for pointing out the typo and the additional references. We corrected both in the revised Models and Methods section: *'Short-term plasticity (STP) is a key component of activity-silent WM models [5, 49, 50, 16, 14, 6].'*

L84: Your model of modulation of synaptic release by astrocytic signaling leverage the TM model and the model previously introduced by De Pitta and co-workers and generalized in De Pitta (Comput. Gliosci. 2019; Ch 10) and De Pitta (Encyclopedia of Comput Neurosci. 2020). However, I am missing the explanation of why LPA synthesis depends on the postsynaptic current (the scheme in Figure 1a does not show it).

Thank you for the comment. It is correct that our model builds on the TM model [18] and is inspired by the modeling work by De Pitta and co-workers. We have clarified the relation of our model to the work of De Pitta et al. [29] in the section where we introduce the model: *'Building on the STP framework by Tsodyks et al. [18] and inspired De Pitta et al.'s model of gliomodulation [29], we construct a synapse model (Figure 1 B) that captures the influence of this LPA-signaling mechanism on synaptic STP.'*

Regarding your second point: In the PRG1-LPA mechanism, LPA synthesis does not depend on the postsynaptic current. Instead, the postsynaptic current influences the amount of LPA binding to presynaptic receptors via its influence on the PRG1-mediated uptake of astrocytically synthesized LPA from the synaptic cleft. Specifically, in the absence of postsynaptic activity, the postsynaptic protein PRG1 balances the ongoing synthesis of LPA by astrocytes and keeps the LPA concentration in the synaptic cleft low by taking up LPA from the cleft. When the postsynaptic Ca2+ levels increase due to postsynaptic activity, PRG1 is inhibited via the influence of Ca2+ on PRG1-inhibitor CaM. As a result, the postsynaptic uptake of LPA from the synaptic cleft decreases, leading to an increased concentration of LPA and, hence, increased LPA binding to the presynaptic LPA receptors. If postsynaptic activity coincides with presynaptic activity, this effect is combined with an increase of astrocytic LPA synthesis due to glutamatergic transmission in the synapse, amplifying the increase of LPA concentration in the synaptic cleft and the resulting effects of presynaptic LPA binding on the presynaptic release probability.

To clarify the previously discussed point in the revised manuscript, we have included a more precise explanation of the postsynaptic element of the PRG1-LPA mechanism in the caption of Figure 1:

'The astrocytic and postsynaptic components modulate presynaptic calcium-binding rates by increasing the concentration of LPA in the synaptic cleft, which allows more LPA in the synaptic cleft (orange) to bind to the presynaptic LPA2 receptors. Postsynaptic calcium increases LPA concentrations in the cleft by inhibiting the postsynaptic protein PRG1 (via CaM) that usually takes up newly synthesized LPA from the cleft. Astrocytic, ATX-mediated synthesis of LPA is amplified in a transmission-dependent way, since some of the synaptically transmitted glutamate binds to astrocytic receptors that increase ATX activity.'

We have also clarified the potentially misleading formulation in the Models and Methods section of the revised manuscript: *'Both the postsynaptic protein PRG1 and the astrocytic enzyme ATX modulate the LPA concentration in the synaptic cleft, which affects binding to presynaptic LPA-receptors that in turn modulate presynaptic vesicle release probability in glutamatergic excitatory synapses [42, 43] (Figure 1 A).'* We hope that this clarifies that the postsynaptic contribution to LPA-mediated presynaptic modulation is due to its influence on the LPA concentration in the synaptic cleft (not on the astrocytic LPA synthesis).

L78-L107. This section seems more to explain your model and should be separated from the results. I would advise you to re-integrate Methods and Models before the Results and provide concise explanations in a discursive fashion about your different models (synaptic/spiking w/ astrocytic signaling + network models). The way models are presented in the end and in a lumped table-like fashion does not help understand your work.

Thank you for the suggestion. In the revised manuscript, we followed your advice and introduced a section 'Models and Methods' prior to the presentation of the results.

To clarify the presentation of our models, we subdivided the new 'Models and Methods' section into three subsections that focus on the synapse models with and without LPA signaling and the network models, respectively. The latter starts with an overview over the four different types of network models used:

'We use two models of synaptic WM: one with and one without astrocytic modulation of STP. To allow for different methods of analysis, we implement each as a rate and a spiking network model, respectively (Figure 1 C, D). For the synaptic WM model without astrocytic modulation, we re-implemented the synaptic attractor spiking and rate model introduced in the seminal work of Mongillo et. al [5]. In the following, we will refer to them as STP spiking and STP rate network, respectively. To model the influence of the astrocytic LPA mechanism on WM, we integrate our STP synapse model with LPA signaling as described in the previous section (Equations 1, 2, 4, 5) in the synaptic STP spiking and rate models by Mongillo et. al [5]. We will refer to them as STP+LPA spiking and rate networks, respectively. See Figure 1 C and D for illustrations and detailed description of spiking and rate network architectures. A full model description is also provided in sections 'Spiking network: model and parameters' and 'Firing rate network: model and parameters' of the SI and in Table S1, Table S3.'.

In the referenced SI section, we explain the four network models, i.e. STP and STP+LPA spiking model and STP and STP+LPA rate model, in a discursive fashion.

Regarding the tabular presentation of our models according to the reproducibility framework by Nordlie et al. [58], we received conflicting feedback from the different reviewers. In the revised manuscript, we found the following compromise between the reviewer opinions: We provided a more extended description of our models in the new and restructured 'Models

and Methods' section to improve the self-consistency, readability and logical flow of the main manuscript. We also kept the tabular model presentation in the interest of model reproducibility. Instead of the method section, it is now part of the SI to which we refer the reader for additional details at appropriate points in the 'Models and Methods' section.

L140: Why infinity? What induces it? (I assume persistent activity emerges, but then clarify).

Thank you for raising this clarifying question. We addressed the comment by rephrasing the respective paragraph in the revised manuscript: *'As U increases, both rate and spiking networks shift from silent WM representations (zero duration) to transient WM representations of continuously increasing duration (Figure 4 A-1, B-1). When the duration of transient representations exceeds the observation window, we refer to them as persistent WM representations.'*

L160–175: Address in two separate paragraphs: first the stabilizing effect of noise, then the destabilizing one.

Thank you for the helpful suggestion regarding the paragraph structure. We followed your advice in the results section of the new manuscript, where we explain the stabilizing and destabilizing effects of noise in two paragraphs: one for stabilizing noise and one for destabilizing noise.

Also, include essential equations for the different noise sources (and what biophysically motivates them).

Thank you for the helpful suggestion. We included the noise equations (Equation 6-8) in the discursive discussion of our network models in the SI section 'Firing rate network: model and parameters' and reference them both in the Models and Methods and in the Results section.

We also agree that we should more clearly explain the sources of noise and their biophysical motivation in the results. We have added the following paragraph in the beginning of the respective results section:

> *'To identify the underlying network mechanisms, we consider STP rate networks (see Figure 1 D) with three different sources of noise: noise in the amount of presynaptically bound calcium y (Equation 6), noise in the amount of available neurotransmitter vesicles x (Equation 7), and noise in the network activity E (Equation 8). The assumption of noise in the synaptic variables x and y is biophysically motivated by the intrinsic stochasticity of the molecular processes by which calcium binds to the presynaptic active sites and neurotransmitter vesicles are released, recycled and refilled in biological synapses. The noise in the network activity E reflects the 'network noise' that arises in biological (and spiking networks) from the stochastic spike generation process, distributed synaptic delays and other sources of randomness in the network interactions.'*

L176: What nonlinearity? Please fully develop this crucial argument and expand your analysis.

Thank you for pointing our the need for further explanation of this argument. We rephrased the respective paragraph in the results section, where we now write:

> *'The impact of stabilizing and destabilizing noise effects depends on the nonlinear dynamics of the system close to the separatrix (see Figures S5-S7). For example, the STP rate network dynamics are nonlinear around the separatrix in the sense that positive fluctuations along the dimension of the fast variable E have very large effects, while negative fluctuations along E have very small effects. This nonlinear*

behaviour (also along the y and x dimensions) are reflected by the length of the flow arrows in the phase plots (Figure S10). The nonlinear dynamics of the network activity E close to the separatrix explain why adding noise to the population activity E tends to stabilize transient WM activity (Figure S6): Negative fluctuations along E, which would prevent new population spikes by destabilizing the limit cycle in the bistability regime, have very small effect on the dynamics trajectory. In contrast, already small positive fluctuations of E in the fixed point regime can induce a cycle in the phase space (i.e. a population spike in the network activity), following the 'ghost' of the limit cycle (see Figure S10 and Refs. [55, 23]). In contrast, adding noise to the synaptic variable y tends to disrupt transient WM activity (Figure S8). This is because a noise-induced decrease of y can push the fast E-x-system to cross the separatrix towards the fixed point and cause a subsequent collapse of oscillatory activity (Figure S10). This explains why noise can have predominantly stabilizing or destabilizing effects pending on the source of the noise, i.e. whether we assume noise in x, y or E.'

L205+: It should be a new paragraph (if not even a new section).

Thank you for the helpful suggestion. To address the referee's comment, we now present the STP+LPA network results in two sections that show the effects of LPA signaling on transient WM representations for weak and strong astrocytic feedback, respectively. In each of the sections, we dedicate a separate paragraph to the discussion of the synaptic dynamics during the transient regime. In this context, we connect the insights from the separation of timescales and the resulting phase plane with the behaviour of the synaptic variables in the respective system with noise.

L210-214: Unclear. Please rephrase and make an effort to provide an interpretation in terms of biophysical mechanisms.

Thank you for pointing out the need for clarification and the benefits of a biophysical interpretation in this paragraph. As suggested, we rephrased the respective paragraph and now write:

'The synaptic dynamics of the underlying noise-free rate network (Figure 6 A-5, A-6) show that the low-duration bump can be explained by the synaptic dynamics of the slow variable ℓ. In particular, the slow ramping in combination with the long timescale of ℓ introduces a 'window of vulnerability' at short durations, where transients are more susceptible to disruption by noise, before gaining stability at longer durations. For the phase space analysis of the synaptic dynamics, we separate the fast dynamics of population activity E and depression x from the slow variables y and ℓ (see Methods) as in the previous section. The trajectories of the slow variables reveal that after the offset of the cue stimulus, y decreases and starts approaching the separatrix between limit cycle (where y is higher) and fixed point (where y is lower). In this brief regime of low y values, noise can push the trajectory over the separatrix into the fixed point region, which results in the termination of the transient WM activity. Meanwhile, ℓ slowly ramps up due to the repeated population spikes, and causes y to increase again after the initial low-y regime. This pushes the trajectory of synaptic dynamics away from the fixed point-region and stabilizes the transient representation. This stabilizing effect of ℓ is so strong that the WM representation that survive the initial low-y time window persist until the end of the observation window. We also refer to the low-y phase as 'window

of vulnerability' during which noise can disrupt a transient WM representation and after which the representation stabilized. '.

To provide a biophysical interpretation of the phase space dynamics, we explain how they relate to the dynamics of the synaptic variables (and the biological mechanisms that they represent) over time: *'This mechanism is reflected in the traces of the synaptic variables over time (Figure 6 A-2, A-3 and B-2, B-3). Calcium binding y decreases immediately after the offset of the cue, destabilizing the oscillations. Here, noise can easily disrupt the transient activity (Figure 6 A-2, B-2). Synaptic LPA ℓ increases gradually after the offset of the cue stimulus (orange traces in Figure 6 A-2, A-3 and B-2, B-3) as long as the memory is active, and pushes the release probability back y up (after 5 seconds after cue stimulus in Figure 6 A-3, B-3). This stabilizes the transient WM activity and makes it more robust against disruption by noise.'*.

L230-245: This paragraph stormed me by surprise. You suddenly mention pathology and epilepsy. It seems off-topic.

Thank for pointing out that this paragraph was not sufficiently motivated in the old manuscript and could break the logical flow of the discussion. To address the referee's comment, we removed the paragraph from the discussion. Since we think that the topic of astrocyte-related pathologies of WM could be relevant to readers with an interest in molecular signaling-related pathologies of WM, we included a rephrased version of the paragraph in the SI and cross-reference it in the discussion paragraph on possible extensions of our study: *'Another interesting direction for future research is the question how pathologies related to astrocytic signaling could impact WM (see section 'Potential impact of PRG-associated pathologies on WM' in the SI)'*.

We also rephrased the paragraph in the SI to clarify the relevance for our study. Specifically, we begin by motivating the topic of astrocyte-related pathologies of WM: *'In the context of functional and metabolic roles of astrocyte-modulated WM, it is also relevant to consider how pathologies of astrocytic signaling could negatively affect the function of WM. For example, genetic ablation of the presynaptic molecule CHL1 in mice [62] has been shown to cause reduced WM durations. CHL1 is involved in presynaptic vesicle recycling and, like PRG1 in the LPA signaling mechanisms, it directly affects synaptic transmission and short-term plasticity. For the LPA signaling mechanism that we consider in this study, the effects on WM are not yet known.'* We then continue by clarifying how such scenarios could be modelled using our framework and what observations we predict: *'However, it is known that in PRG1-KO mice, the dysfunction of the PRG1 protein prevents LPA-uptake from the cleft while the ATX-mediated synthesis of new LPA remains unaffected. As a result, the LPA concentration rises and allows a pathologically high amount of LPA to bind to presynaptic receptors. We can capture such scenarios in our model by applying different M values to model WT (low M), partial PRG-KO (intermediate M) and full PRG-KO (high M) condition. In the steady state, elevated M values lead to an increase of the effective baseline release probability U. As shown in Figure S2, the synaptic model then reproduces qualitatively the decrease of the PPR of pathological (PRG1-KO) versus healthy (WT) conditions in depressing and facilitating synapses. In a spiking network, the increase of the effective baseline release probability U leads to increased spontaneous network activity (Figure S3). This can be linked to the hyperexcitability measured in PRG1-KO mice, where it leads to seizures and impaired sensory processing [44].'*.

L279-286: Split. Address points 1 (decision window) and 2 (energetic benefits) in two separate paragraphs.

Thank you for this helpful suggestion. Since the two points respectively reflect the functional and the metabolic effects of the LPA-mediated 'window of vulnerability', we perceive them as closely related concepts and therefore prefer to describe them in a single paragraph. However, we fully appreciate the need to explain them in more depth and in a more structured way that was raised by the referee. To address this, we have rephrased the respective paragraph in the discussion and now write: *'In practice, the modulation of WM introduced by LPA signaling could have several functions. The 'window of vulnerability' that is introduced by strong LPA signaling effects on presynaptic STP could implement a filtering of WM representations. During the initial 'window of vulnerability', the WM representation is very susceptible to disruption by noise, such that WM representations with slightly higher internal noise would have a high chance to terminate during this period. Such slighty elevated noise could e.g. arise from the presence of more salient or more attended and therefore stronger competing WM representations in the same network. The WM representations that 'survive' this initial period are then stabilized for a longer time to allow for further cognitive processing. This filtering could be relevant for prioritizing and managing the limited mental capacity to hold WM items. At the same time, it could help to save energy by limiting the duration of WM representations that do not require further processing and therefore do not need to be active for a long time – especially since the slow unbinding of LPA allows them to be reactivated from their synaptic traces for a much longer time than in the absence of astrocytic modulation.'*.

L304: You never really introduced the concept of a tripartite synapse. It is barely mentioned in the introduction, but I am afraid you might be too confident to believe that everyone knows what you are talking about.

Thank you for the comment, we agree that the concept was unclear before. When following your suggestion to include a separate paragraph on astrocytic modulation of synaptic transmission in the introduction, we also included an explicit definition of tripartite synapses: *'Over the past decade, an increasing body of evidence has shown that astrocytes influence and interact with synaptic transmission at the presynaptic and postsynaptic side [27, 28, 29]. In these cases, we also speak of tripartite synapses [30].'* We hope that this will sufficiently clarify the concept for the readers.

Equations and notation. For historical reasons, I would stick to the original notation for the facilitating variable, u rather than y. And γ rather than ℓ. Moreover, I would adopt lower case letters for time-dependent quantities and upper case letters for constants. Thus $U \to u_0$ for example.

Thank you for the suggestion. We agree that the variable u would be more consistent with previous work than y. However, for the sake of consistency with our published simulation code, we would like to keep the variable name in this case.

The name for our variable ℓ is motivated by the fact that it represents the amount of presynaptically bound LPA. It chosen so that it would be as easy as possible for readers to remember its meaning and we would therefore like to keep it, even though γ might be more aligned with previous models that focus on other gliotransmission pathways.

We see the value of the convention to use upper case letters for constants. Most of our parameters, including M, ΔU, U_b (LPA) and U (STP) are chosen according to this convention. The only exceptions are the time constants τ_L, τ_F and τ_D, which (despite being constants) are typically used as lower case letters in the related neuroscience and physics literature, and the calcium binding rate $U(t)$ in the synapses with LPA. We would like to keep the upper case $U(t)$ for the latter to clarify the link to the STP model and provide a visually intuitive idea of where the LPA modulation affects the STP model. This is especially relevant since in the

limit of $M \to 0$ and $\Delta U \to 0$, the LPA model is equivalent to the STP model with constant U.

Eq. 2-3: why not to define $r(t_k) = u(t_k^+)x(t_k^-)$?

Thank you for this suggestion. We added the index k to y^- and x^- to clarify the dependency on each spike: *'Here, $t^{(k)}$ are the arrival times of presynaptic spikes; $y_k^- = y(t^{(k),-}), x_k^- = x(t^{(k),-})$ are evaluated immediately before the k-th spike arrival, and $y_k^+ = y(t^{(k),+})$ is evaluated immediately after the k-th spike arrival.'* In the revised Models and Methods section, we also introduce the idea that $y_k^- x_k^-$ can be interpreted as the synaptic release rate associated with the transmission of a presynaptic spike at time $t^{(k)}$: *'We also refer to $y_k^+ x_k^-$ as the release rate for the k-th presynaptic spike.'* However, since we do not use $y_k^- x_k^-$ very often and would like to keep the number of variables introduced in the manuscript to a minimum, we decided not to assign a separate variable name to the expression.

Eq. 5: It must be $\Delta U > U_b$ in your model to reproduce your results if I am not mistaken. On the other hand, since U must be in [0,1], the choice ΔU is subjected to constraints that should be stated.

Thank you for pointing this out, we explain the constraints on ΔU in the Models and Methods section of the revised manuscript: *'To stay consistent with the STP model, where $U(t) \in [0, 1]$, the choice of the parameter ΔU is limited to the interval $[0, 1 - U_b]$ for a given baseline calcium-binding rate $U_b \in [0, 1]$.'*

There is some inconsistency in notation: e.g., sometimes ℓ and other times l (cp. Figures in the main text with Fig. S1 legends).

Thank you for spotting this. We have streamlined our notation and now consistently refer to the amount of presynaptic LPA binding as ℓ.

Panels are sometimes labeled by lower case letters and upper case ones. Please be consistent in your style choices.

Thank you for the comment. We removed the inconsistency in our new manuscript and now label all panels with upper case letters.

Figures. Figure 1 is somehow misleading. You show the synaptic model, but you provide population activity plots (when no neurons are shown in the scheme). I assume that you are using the rate model here? Otherwise, I cannot justify, unless by averaging, how you can show lumped synaptic variables.

Thank you for raising this point. Fig. 1C in the old manuscript indeed shows simulations of the STP+LPA rate network. This was done to illustrate the type of synaptic and network activities that arise in all four networks (STP/STP+LPA rate/spiking) during our WM protocol but we agree that the setup was not clearly shown or explained. To clarify this point, we restructured Figure 1 as follows: In addition to sketches of the synaptic mechanism and model (Fig. 1 A and B), we show the architecture of the two network models (spiking network in Fig. 1 C, rate network in Fig. 1 D). In addition, we introduce Fig. 2, where we present characteristic network dynamics that all four types of networks exhibit during the WM protocol, using the representative example of an STP+LPA spiking network.

Figure 2. A: Are those population rates? B and C: What are the barely visible gray data? D: Replot as a 2D heatmap. It would help if you showed the cue on the plots in A, as you do elsewhere.

Thank you for the valuable advice on how to improve the readability of our figure. Due to the major restructuring of our results section and figures for the new manuscript, the individual panels of Figure 2 A (old manuscript) now appear in a revised design as Figure 2 A (previous panel 2 A), Figure 4 B-1 (previous panel 2 B) and Figure 5 B-1 (previous panel 2 D).
Regarding old Figure 2A: You are correct to assume that these are population rates, which we obtained from the spiking simulations by population averaging. We clarify this point in the revised manuscript both in the figure capture of new Figure 2 A and in the text describing the figure.
We also recognize that the light grey bar with which we indicate the cue presentation in the old Figure 2 A was not visible and interpretable enough. We addressed this in the new Figure 2 A by intensifying the color of the grey bar and adding a label explaining its meaning directly in the plot.
Regarding old Figure 2 B and C: The light grey dots represent the individual data points in the distribution of transient durations for each value of U (old Fig. 2 B) and τ_L (old Fig. 2 C). To address the referee's point, we have redesigned the plot in old Fig. 2 B and C as shown in new Fig. 4 A-1, B-1, new Fig. 5 A-1, B-1 and new Fig. 6 A-1, B-1. We now plot the data points in black and in larger size to make them more visible. We have also removed the median and standard deviation to increase readability and highlight the shape of the distribution for different values of U and τ_L. We explain the meaning of the dots in the figure caption and in the text, e.g. *'Figure 4 A shows the distributions of durations of active WM representations for changing calcium binding rate U in the STP rate network with noise. Each point in the scatter plot corresponds to one WM simulation (as depicted in the examples in Figure 4 B, C) in an STP rate network with U-value as specified on the x-axis.'*
Regarding old Fig. 2 D: Thank you for the suggestion. We have taken the referee's advice to illustrate the data in a 2-D instead of a 3-D plot. To keep the plot visually simple and still show the contribution of each individual simulation, we have opted for a 2-D multi-distribution plot (Fig. 4 A-1, B-1, new Fig. 5 A-1, B-1 and new Fig. 6 A-1, B-1).

Figure 3. Put some color panels together: B should be orange simulations + orange phase space trajectories; C should be the same for red ones. Do you need to plot in 3D?

Thank you for the helpful suggestions. To clarify the link between the matching simulation and phase space trajectories for stabilizing and destabilizing noise, we now added labels ('Stabilizing noise' and 'Destabilizing noise') both above the phase space and simulation plots. For further clarification, we also split panels B and C into B-1 and B-2 and C-1 and C-2, respectively. We decided against rearranging the panels since that would have made it difficult to position the bifurcation plot (panel A) in the same figure. We also wanted to keep the simulations (panel B) next to the bifurcation plot to make it more easily interpretable.
Regarding the 3D plot: We followed the referee's suggestion to plot the 2D-projection of the phase space for the remaining result figures (Fig. 4 and Fig. 6) and in the SI. Since, in Figure 3, we explain the phase phase dynamics for the first time, we decided to stick to the 3D plots here. This allows us to display a more precise picture of the dynamics in its full dimensionality and to visually distinguish it from the dynamics in related 2D STP systems with only facilitation or only depression.

Figure 4. Arguably split A and B into two figures. Here again, it is not clear why you show me a synaptic scheme, but the simulations appear to be in the network. I cannot follow your reasoning and cannot easily match the bifurcation diagrams in A/B5 with the simulations in A/6.

Thank you for these helpful suggestions and comments. In the revised manuscript, we address the referee's points as follows.

First, we followed the referee's suggestion and split the previous Figure 4 into two separate figures: new Figure 4 and 6 respectively integrate the revised versions of old Figure 4 A (STP rate model) and old Figure 4 B (STP+LPA rate model). Inspired by the referee's comment, we further introduced Figure 5, which allows us to illustrate the two qualitatively different regimes of STP+LPA networks with weak and strong LPA signaling in separate figures, respectively.
Second, we removed the synaptic schemes from both panels. They were intended to help distinguish simulations from the STP and STP+LPA network, but the referee's comment made us realize that they could instead lead to confusion about whether simulations are performed in single synapses or networks. Implementing other suggestions of this and the other referee's, we found alternatives to clarify the distinction between STP and STP+LPA network simulations (see answers to respective referee comment).
We appreciate the referee's comment regarding the links between A/B5 and A/B6 that were not made sufficiently clear in the old manuscript. We address it two-fold. First, we restructured the figure to separate the observations from the network model simulations with noise and the phase space trajectories in the underlying noise-free network model (Fig. 4 A, 5 A, 6 A). Second, we carefully rewrote the respective three results sections to include a more extensive explanation of the figures, and how the trajectories in the noise-free model can help us understand the behavior in the model with noise.

Figure 5. A: Why upward arrows for B and C? B and C: take out the scheme from the plots: keep your plots as clean as possible. I do not understand the yellow dots. Left panels for Calcium- binding and LPA binding are unreadable to me. You do not need to comment on your plots (e.g., 'High LPA...') since you should address the full explanation of your figures in the Results.

Thank you for the helpful comments. In the revised manuscript, we followed the referee's suggestions in adjusting the presentation of former Fig. 5 B, C (now part of Fig. 5 and 6). We also included the old Fig. 5 as Fig. S2, where we included the referee's additional comments. In particular, we replaced the upward arrows with up-and-downward arrows to indicate that the system can move through the transition in both directions. We removed the synapse model sketches and the unnecessary color coding, and resized the dots and figure dimensions of the left panels to improve readability. To support intuitive understanding of the middle and right panels of former Fig. 5 in the new Fig. S2, and to avoid that the reader needs to flip back and forth between Results and Supplement, we decided to keep the comments in these plots for now. However, we are of course open to revise the plots again if necessary.

Figure 6. It is a nice idea but only marginally discussed. Please elaborate in a fully-developed paragraph.

Thank you for the helpful suggestion on how to clarify the message of the figure. In the revised manuscript, we follow the referee's advice and explain the figure and its message in a dedicated paragraph of the discussion.

Figure S1. A: Error bars are barely visible. B: What is the pre-synaptic stimulus? You should arrange these figures vertically, in four columns of three panels each (pre — syn vars — post).

Thank you for the helpful comments to improve Fig. S1. In the revised manuscript, we increased the size of the error bars, included the presynaptic stimulation protocol and rearranged the figure panels as suggested by the referee.

Figure S4. Compared with analogous plots in Figure 2, you are showing STD rather than quartiles. And the median does have little use. Please choose one style and keep it consistent throughout all your figures.

Thank you for the helpful suggestions. Following suggestions by this and the other referee's, we have redesigned the distribution plots of the transient WM duration (old Fig. 2) and now consistently display them as scatter plots in the main manuscript (see Figs. 4-6). To make the distribution plots within the SI consistent, we followed the referee's advice and removed the median from the plots in Fig. S4.

Figures S6–8: Gray lines are barely visible. Along with Figures S9 and S15, reconsider plotting all data points by box plots or violin plots along with extrema as well.

Thank you for the comments. We improved the contrast and increased the line thickness of the grey lines in S6-S8. We have carefully considered and tested the suggestion to use violin plots. However, to remain consistent with the distribution plots in the main manuscript and given the high number of values on the x-axis, we have decided to keep the current visualization for now. Of course, we would gladly remake the figure again if that does not sufficiently address the referee's comment.

Figures S12–S14: what are the shades? SDs? SEMs? Please clarify. Improve contrast.

Thank you for the comments. We extended the description in the respective figure captions to clarify the meaning of the shades (standard deviation) in S12 B, S13, and S14, e.g. we now write in the caption of Fig. S12 B: *'We compare two groups: Simulations which did not terminate ('uninterrupted') within 20 seconds, and simulations which did terminate after at least 15 population spikes. Solid lines represent the mean, shades the standard deviation for each group.'*
We also added explanations for the dark and light grey-shaded bars in S12 A: *'Left: Full time course, dark grey bar indicates the stimulation with the WM cue. Center/Right: Population activity (center) and synaptic variables (right) in a section around three population spikes. Here, the light grey areas show the time interval of $200ms$ preceding each population spike.'*

Reviewer 3

The authors study the effect of lipid modulation on the time scales of short-term plasticity in a model of working memory. They use simulations in a spiking model and analysis using a firing rate model. They show how the modulation may influence WM duration, enhance WM robustness to noise (either preventing spurious induction or termination), and they discuss possible roles in top-down modulation of WM. The article is well written and work appears to be scientifically correct. The authors has made a very good effort to make the models understandable by providing the information according to Nordlie.

We thank the referee for the appreciation of our work and for the careful and thoughtful review of our manuscript. We have revised our manuscript to incorporate the referee's suggestions. Please find below our point-by-point reply.

Major points: The problems in working memory that you address and your solutions to these problems need to be provided throughout your text (abstract, introduction). Consider your points about your main findings raised in the Discussion and formulate your research problem around this.

Thank you for the helpful suggestions. We took several steps to address the referee's comment and clarify the main questions and problems in WM that are addressed by our study.

- We rephrased the abstract to clarify the open problem we address in this work: *'Short-term synaptic plasticity and modulations of the presynaptic vesicle release rate are key*

components of many working memory models. At the same time, an increasing number of studies suggests a potential role of astrocytes in modulating higher cognitive function through their influence on synaptic transmission. Which influence astrocytic signaling could have on WM representations, however, is still unclear.' We also provided a more detailed description of our methodology, findings and conclusions in the revised abstract.

- We restructured and rephrased the text in the introduction to more explicitly point out the open problems in WM research that we address. For example, we write: *' So far, however, it is unclear whether and how this spontaneous termination of WM can be controlled'*, and: *'[...] astrocytic modulation is potentially relevant for the coordination of network activity [37] and could influence high-level neural computations [38]. Here, we investigate how the long timescales of a specific signaling pathway at the tripartite synapse affects the stability and duration of meta-stable active WM representations, i.e. active WM representations that are induced by a cue stimulus but terminate naturally through noise, and explain the network mechanisms underlying the observed phenomena.'* Furthermore, in the last paragraph of the introduction, we condense the open problems presented in the previous paragraphs into three main research questions: *'In particular, we answer the following three questions: (i) What role do different sources of noise have for the WM encoding, i.e. for the stability of the active WM representation? [...] (ii) How does astrocytic modulation of presynaptic STP impact the stability and duration of active WM representations? [...] (iii) Finally, what is the specific role of the slow astrocytic timescale? [...].'*.
- We refer back to these three questions throughout the remaining manuscript. In particular, we restate the three questions in the introductory paragraph of the discussion and discuss how our findings answer each question in the subsequent three paragraphs.

More specifically see 1-3 below: 1) On line 142-142 you provide a good explicit statement. Please follow this and make similar statements for 2) and 3) below. 'In contrast, in the networks with LPA signaling, a slow and broadly stable transition from the silent to a transient regime is possible.'
2) Section 'Transient WM activity emerges from silent and persistent network dynamics due to noise'. You provide observations. Please remind the reader of what 'problem' you want to address? Please provide explicit statements as outlined above.

Thank you for your thoughtful comment. In the new manuscript, we rephrased the text of the section 'Noise can induce transient WM in silent and persistent network regimes' to emphasize the questions addressed in the section and to present the answers more clearly. In particular, we highlight the main question addressed by this section in the first sentences: *'In this section, we study by which network mechanisms noise can lead to the stochastic termination of WM representations. To identify the underlying network mechanisms, we consider STP rate networks (see Figure 1 D) with three different sources of noise: [...]'* Throughout the remaining text of the section, we return to this initial question and provide explicit answers.

3) Furthermore, in the section 'LPA signaling modulates the distribution of transient durations at the transition from the silent to the persistent state' Make more explicit statements of what your model with LPA-modulation results in, what 'problem' do you address and what effect/solution do you observe?

Thank you for the helpful remark. We have taken several steps to address the referee's point and clarify the main problem and effect/solution of including LPA signaling in our model:

- As part of an overall restructuring of the manuscript, we have divided the previous section 'LPA signaling modulates the distribution of transient durations at the transition from the silent to the persistent state' into three parts. In particular, we present the influence of synaptic parameters on the STP model and the STP+LPA model in separate sections: 'Effects of STP parameters on the mean transient WM duration', 'Weak LPA signaling implements slow modulation of the mean transient WM duration via its influence on presynaptic calcium binding' and 'Strong astrocytic signaling introduces bimodal distribution of transient WM durations and 'window of vulnerability' for WM termination'. This allows us to discuss the effects of including LPA more rigorously.
- We have also rephrased our presentation of STP and PRG results in each of the sections. In particular, we now include a short introductory paragraph that states how the current section helps to answer the lead questions from the hypothesis section. For example, we now write: *'[...] noise alone does not explain why STP networks with and without LPA signaling show qualitatively different distributions of transient durations (Figure 2 B). In the following sections, we will shed light on the mechanisms underlying this phenomenon. The influence of LPA signaling on STP dynamics is mediated by its influence on the calcium binding rate U (Equations 4, 5). Therefore, we begin by investigating how the calcium binding rate U (and other STP parameters) influence the distribution of transient WM durations in STP rate and spiking networks.'*
- Throughout the text, we restated the main results and effects of LPA signaling on the model behavior in short and clear sentences.

Induction and maintenance or WM studied has been extensivley, including tolerance to noise or distractors. However, termination of activity has been addressed to a lesser extent. In this regard, you mention volitional control of WM which I think is an important point. There are two sides of the coin, providing stability to perturbations but at the same time allowing a possibility to end working memory. With regard to ending the activity, even if animal experiments of WM has shown results for delay periods even up 60 seconds, typically time scales of just a few seconds are used both for delay periods and inter-trial intervals, see e.g. this study on humans: Teki Sundeep, Griffiths Timothy D. Working memory for time intervals in auditory rhythmic sequences. Frontiers in Psychology 5, 2014, DOI=10.3389/fpsyg.2014.01329 This issue of short time scales also includes continuous versions of the task (cDNMS) used in rodent experiments where working memory has to be continiously updated, e.g. Young BJ, Otto T, Fox GD, Eichenbaum H. Memory representation within the parahippocampal region. J Neurosci. 1997;17(13):5183-5195. doi:10.1523/JNEUROSCI.17-13-05183.1997. Here is finally my question: Your model adds a slow time scale (seconds-minute). Is there any risk of getting 'stuck' in a too stable or hard-to-terminate state? Does this slow time-scale prevent you from following faster-changing working memory protocols?

Thank you for these interesting questions. We have included our answer as part of the discussion: *'Whilst the LPA unbinding timescale has a long-term stabilizing effect on synaptic WM, it does not cause the WM dynamics to get stuck. Even in the case of strong LPA signaling, the LPA-stabilized persistent WM representations can be terminated by a single, weak off-stimulus, just like in the case of regular persistent WM representations. Since the transmission-dependent ramping of LPA is also slow, there is also no limitation to follow faster WM protocols. However, in such protocols, the slow effects of LPA signaling would probably be only marginally observable since they start showing effect only several seconds after the cue offset.'*

Minor points: Figure 1A. Complement the signalling network figure 1A to indicate enhancing (+) or suppressive (-) effects on targets (LPA2r, ATX, PRG1, "active zone") via messengers (LPA, Ca2+, Glu) and indicate effect on targets. Specifically separating effects on messenger concentration or time constant of some process. Also illustrate your minimal model implementing this signalling (eq 4,5).

Thank you for the helpful suggestion. We included an illustration of our minimal model (Fig. 1B) in addition to the sketch of the synaptic mechanism. Given the already high complexity of the biological sketch in Fig. 1A, we indicated the enhancing/suppressive nature of the variable interactions in this sketch of the minimal model instead. Our sketch is inspired by an illustration of the STP mechanism in Mongillo et al. [5] that we found to be very clear. In our sketch, we depict for each of the synaptic variables x, y and ℓ which parameters and variables govern their enhancement (arrow from $1-x$ to x, from $1-y$ to y and $1-\ell$ to ℓ) and their suppression (arrows in opposite directions, respectively).

line 103-104 'the LPA-mediated effects in the presynapse occur on longer timescales of several seconds to a minute [45].' Please explain how you arrived at this time scale for LHA. As far as I understand the article [45] discusses Ca2+ transients on that time scale.

Thank you for your comment, we agree that this is a very important point. As you correctly noted, Berridge et al. [51] discuss Ca2+ transients that decay over long timescales, which includes the Ca2+ transients that are triggered by LPA binding to the presynaptic LPA2 receptors. In particular, they write: *'The dynamics of Ins(1,4,5)P3 production can be very different depending on the receptor type being activated6. Bradykinin and neurokinin A receptors give large rapid Ca2+ transients, whereas lysophosphatidic acid (LPA), thrombin and histamine receptors give smaller responses that develop slowly but persist for much longer'* [51]. For the duration of these LPA2-R induced intracellular Ca2+ transients, the higher availability of Ca2+ in the presynaptic compartment naturally increases the probability of Ca2+ binding to the presynaptic active zones. Since the presynaptic release of neurotransmitters is mediated via calcium binding to the presynaptic active sites, it is biologically plausible to assume that the LPA-induced increase of the presynaptic release probability persists on similar timescales as the LPA-induced intracellular Ca2+ transients that cause increased presynaptic Ca2+ binding to the active sites. In our model, this is represented as an ℓ-dependent increase of the Ca2+ binding probability U that (since ℓ and U are linearly related) persists on longer timescales τ_L. To address the referee's comment, we included the section 'Biological evidence for slow LPA timescales' in the SI, where we provide the above explanation of the relation between slow LPA-induced transients and the timescale of LPA-mediated effects on short-term synaptic plasticity.

Regarding reference to Sreenivasan, K.K., D'Esposito, M. The what, where and how of delay activity. Nat Rev Neurosci 20, 466–481 (2019). https://doi.org/10.1038/s41583-019-0176-7 Since you cite this article, it would be suitable to also cite the modeling work discussed: Pittà, M. D., De Pittà, M., Ben-Jacob, E. & Berry, H. Astrocytic theory of working memory. BMC Neurosci. 15 (Suppl. 1), P206 (2014). Please discuss any similarities or differences between this model and your model.

Thank you for pointing us to this important reference. We cite it in the discussion, where we also set it in relation to our model: *'Both mechanisms constitute candidate pathways for the volitional control of WM [12, 59, 60], e.g. from the beta-band activity of deep layers to superficial layers [59]. A previously proposed type of volitional WM control through astrocytic modulation of STP suggests that astrocytes could switch the WM regime from silent into persistent mode [61]. The type of volitional control we propose here is different: it affects*

WM more subtly by modulating the duration and stability of active representations. Both types address equally important aspects of volitional WM control, and we can speculate that neuronal networks in different brain regions would either (i) implement volitional WM control of one or the other type, depending on their neuronal and synaptic architecture; or (ii) switch between them depending on the level of surrounding global brain activity.'

Fig S1. Your figure shows ISIs of 50, 100 and 200 ms. You provide a reference to ref 44. This artile uses the ISI of 500 ms. Please explain the data behind your figure.

Thank you for spotting this. In the old manuscript, we accidentally cited the article by Vogt et al. from 2016 [47] instead of the correct article from 2017 [63] for the facilitating synapse case. We corrected this in the revised manuscript (caption of Fig. S3). The data for the depressing synapses was correctly cited, it is taken from Fig. 6 in Unichenko et al. [43].

Ref 41, 44 Both these references report work on hererozygous knock-out animals. This means that only one gene was deleted and the other gene was left functional. Naively speaking, this would render the protein content at 50%, so it would not mean the protein is down to 0%. Does that affect your model an any way?

Thank you for the relevant question. Both a full and partial PRG1-KO condition can be modelled using our framework. We rephrased the respective paragraph of the discussion to incorporate the distinction between full and partial PRG1-KO condition suggested by the referee: *'[...] However, it is known that in PRG1-KO mice, the dysfunction of the PRG1 protein prevents LPA-uptake from the cleft while the ATX-mediated synthesis of new LPA remains unaffected. As a result, the LPA concentration rises and allows a pathologically high amount of LPA to bind to presynaptic receptors. We can capture such scenarios in our model by applying different M values to model WT (low M), partial PRG-KO (intermediate M) and full PRG-KO (high M) condition.'*

References

[1] Fuster JM, Alexander GE. Neuron Activity Related to Short-Term Memory. Science. 1971;173(3997):652–654.

[2] Funahashi S, Bruce CJ, Goldman-Rakic PS. Mnemonic Coding of Visual Space in the Monkey's Dorsolateral Prefrontal Cortex. J Neurophysiol. 1989;61(2):331– 349.

[3] Christophel TB, Christiaan Klink P, Spitzer B, Roelfsema PR, Haynes JD. The Distributed Nature of Working Memory. Trends Cogn Sci. 2017;21(2):111–124.

[4] Murray JD, Jaramillo J, Wang XJ. Working Memory and Decision-Making in a Frontoparietal Circuit Model. J Neurosci. 2017;37(50):12167–12186.

[5] Mongillo G, Barak O, Tsodyks M. Synaptic Theory of Working Memory. Science. 2008;319(5869):1543–1546.

[6] Barbosa J, Stein H, Martinez RL, Galan-Gadea A, Li S, Dalmau J, et al. Interplay between persistent activity and activity-silent dynamics in the prefrontal cortex underlies serial biases in working memory. Nat Neurosci. 2020;23:1016–1024.

[7] Harvey CD, Coen P, Tank DW. Choice-specific sequences in parietal cortex during a virtual-navigation decision task. Nature. 2012;484(7392):62–68.

[8] Schmitt LI, Wimmer RD, Nakajima M, Happ M, Mofakham S, Halassa MM. Thalamic amplification of cortical connectivity sustains attentional control. Nature. 2017;545(7653):219–223.

[9] Druckmann S, Chklovskii DB. Neuronal circuits underlying persistent representations despite time varying activity. Curr Biol. 2012;22:2095–2103.

[10] Lisman JE, Jensen O. The theta-gamma neural code. Neuron. 2013;77(6):1002–1016.

[11] Lundqvist M, Rose J, Herman P, Brincat SL, Buschman TJ, Miller EK. Gamma and Beta Bursts Underlie Working Memory. Neuron. 2016;90(1):152–164.

[12] Lundqvist M, Herman P, Warden MR, Brincat SL, Miller EK. Gamma and beta bursts during working memory readout suggest roles in its volitional control. Nat Commun. 2018;9(1):1–12.

[13] Bastos AM, Loonis R, Kornblith S, Lundqvist M, Miller EK. Laminar recordings in frontal cortex suggest distinct layers for maintenance and control of working memory. Proc Natl Acad Sci U S A. 2018;115(5):1117–1122.

[14] Masse NY, Yang GR, Francis Song H, Wang XJ, Freedman DJ. Circuit mechanisms for the maintenance and manipulation of information in working memory. Nat Neurosci. 2019;22:1159–1167.

[15] Stokes MG. 'Activity-silent' working memory in prefrontal cortex: A dynamic coding framework. Trends Cogn Sci. 2015;19(7):394–405.

[16] Trübutschek D, Marti S, Ueberschär H, Dehaene S. Probing the limits of activity-silent non-conscious working memory. Proc Natl Acad Sci U S A. 2019;116(28):14358–14367.

[17] Lundqvist M, Herman P, Miller EK. Working Memory: Delay Activity, Yes! Persistent Activity? Maybe Not. J Neurosci. 2018;38(32):7013–7019.

[18] Tsodyks M, Pawelzik K, Markram H. Neural Networks with Dynamic Synapses. Neural Comput. 1998;10(4):821–835.

[19] Lundqvist M, Herman P, Lansner A. Theta and gamma power increases and alpha/beta power decreases with memory load in an attractor network model. Journal of cognitive neuroscience. 2011;23(10):3008–3020.

[20] Trübutschek D, Marti S, Ojeda A, King JR, Mi Y, Tsodyks M, et al. A theory of working memory without consciousness or sustained activity. Elife. 2017;6:e23871.

[21] Zucker RS, Regehr WG. Short-Term Synaptic Plasticity. Annu Rev Physiol. 2002;64(1):355–405.

[22] Tabak J, Senn W, O'Donovan MJ, Rinzel J. Modeling of spontaneous activity in developing spinal cord using activity-dependent depression in an excitatory network. Journal of Neuroscience. 2000;20(8):3041–3056.

[23] Bart E, Bao S, Holcman D. Modeling the spontaneous activity of the auditory cortex. Journal of computational neuroscience. 2005;19(3):357–378.

[24] Holcman D, Tsodyks M. The emergence of up and down states in cortical networks. PLoS computational biology. 2006;2(3):e23.

[25] Barak O, Tsodyks M. Persistent Activity in Neural Networks with Dynamic Synapses. PLoS Comput Biol. 2007;3(2):e35.

[26] Cortes JM, Desroches M, Rodrigues S, Veltz R, Muñoz MA, Sejnowski TJ. Short-term synaptic plasticity in the deterministic Tsodyks-Markram model leads to unpredictable network dynamics. Proc Natl Acad Sci U S A. 2013;110(41):16610–16615.

[27] Araque A, Carmignoto G, Haydon PG, Oliet SHR, Robitaille R, Volterra A. Gliotransmitters Travel in Time and Space. Neuron. 2014;81:728–739.

[28] Covelo A, Araque A. Lateral regulation of synaptic transmission by astrocytes. Neuroscience. 2016;323:62=66.

[29] De Pitta M, Brunel N, Volterra A. Astrocytes: Orchestrating synaptic plasticity? Neuroscience. 2016;323:46–61.

[30] Araque A, Rita VP, Sanzgiri P, Haydon PG. Tripartite synapses: glia, the unacknowledged partner. Trends in Neurosciences. 1999;22:208–215.

[31] Wei D, Lee K, Li H, Lu R, Bae JA, Liu Z, et al. AxonEM Dataset: 3D Axon Instance Segmentation of Brain Cortical Regions. In: de Bruijne M, Cattin PC, Cotin S, Padoy N, Speidel S, Zheng Y, et al., editors. Medical Image Computing and Computer Assisted Intervention – MICCAI 2021. Springer International Publishing; 2021. p. 175–185.

[32] Jourdain P, Bergersen LH, Bhaukaurally K, Bezzi P, Santello M, Domercq M, et al. Glutamate exocytosis from astrocytes controls synaptic strength. Nat Neurosci. 2007;10:331–339.

[33] Perea G, Yang A, Boyden ES, Sur M. Optogenetic astrocyte activation modulates response selectivity of visual cortex neurons in vivo. Nature Communications. 2014;5.

[34] Perea G, Araque A. Astrocytes Potentiate Transmitter Release at Single Hippocampal Synapses. Science. 2007;317:1083–1086.

[35] Navarrete M, Perea G, de Sevilla DF, Gómez-Gonzalo M, Núñez A, Martín ED, et al. Astrocytes Mediate In Vivo Cholinergic-Induced Synaptic Plasticity. PLOS Biology. 2012;10:e1001259.

[36] Allen NJ, Eroglu C. Cell Biology of Astrocyte-Synapse Interactions. Neuron. 2017;96(3):697–708.

[37] Durkee CA, Araque A. Diversity and Specificity of Astrocyte–neuron Communication. Neuroscience. 2019;396:73–78.

[38] Santello M, Toni N, Volterra A. Astrocyte function from information processing to cognition and cognitive impairment. Nat Neurosci. 2019;22:154–166.

[39] Gordleeva SY, Tsybina YA, Krivonosov MI, Ivanchenko MV, Zaikin AA, Kazantsev VB, et al. Modeling Working Memory in a Spiking Neuron Network Accompanied by Astrocytes. Frontiers in Cellular Neuroscience. 2021;15.

[40] Tsybina Y, Kastalskiy I, Krivonosov M, Zaikin A, Kazantsev V, Gorban A, et al.. Astrocytes mediate analogous memory in a multi-layer neuron-astrocytic network. arXiv; 2021.

[41] Maurizio De Pittá NB. Multiple forms of working memory emerge from synapse-astrocyte interactions. bioRxiv; 2021.

[42] Trimbuch T, Beed P, Vogt J, Schuchmann S, Maier N, Kintscher M, et al. Synaptic PRG-1 Modulates Excitatory Transmission via Lipid Phosphate-Mediated Signaling. Cell. 2009;138(6):1222–1235.

[43] Unichenko P, Kirischuk S, Yang JW, Baumgart J, Roskoden T, Schneider P, et al. Plasticity-related gene 1 affects mouse barrel cortex function via strengthening of glutamatergic thalamocortical transmission. Cereb Cortex. 2016;26(7):3260–3272.

[44] Thalman C, Horta G, Qiao L, Endle H, Tegeder I, Cheng H, et al. Synaptic phospholipids as a new target for cortical hyperexcitability and E/I balance in psychiatric disorders. Nat Mol Psychiatry. 2018;23(8):1699–1710.

[45] Bräuer AU, Savaskan NE, Kühn H, Prehn S, Ninnemann O, Nitsch R. A new phospholipid phosphatase, PRG-1, is involved in axon growth and regenerative sprouting. Nat Neurosci. 2003;6(6):572–578.

[46] Yizhar O, Fenno LE, Prigge M, Schneider F, Davidson TJ, Ogshea DJ, et al. Neocortical excitation/inhibition balance in information processing and social dysfunction. Nature. 2011;477(7363):171–178.

[47] Vogt J, Yang JW, Mobascher A, Cheng J, Li Y, Liu X, et al. Molecular cause and functional impact of altered synaptic lipid signaling due to a prg-1 gene SNP. EMBO Mol Med. 2016;8(1):25–38.

[48] Tsodyks M. Activity-dependent transmission in neocortical synapses. In: Chow C, Gutkin B, Hansel D, Meunier C, Dalibard J, editors. Methods and Models in Neurophysics. vol. 80 of Les Houches. Elsevier; 2005. p. 245–247, 249–265.

[49] Mongillo G, Hansel D, van Vreeswijk C. Bistability and Spatiotemporal Irregularity in Neuronal Networks with Nonlinear Synaptic Transmission. Phys Rev Lett. 2012 Apr;108:158101.

[50] Hansel D, Mato G. Short-Term Plasticity Explains Irregular Persistent Activity in Working Memory Tasks. Journal of Neuroscience. 2013:133–149.

[51] Berridge MJ, Bootman MD, Roderick HL. Calcium signalling: dynamics, homeostasis and remodelling. Nat Rev Mol Cell Biol. 2003;4(7):517–529.

[52] Keune WJ, Hausmann J, Bolier R, Tolenaars D, Kremer A, Heidebrecht T, et al. Steroid binding to Autotaxin links bile salts and lysophosphatidic acid signalling. Nat Commun. 2016;7(1):11248.

[53] Tokumitsu H, Hatano N, Tsuchiya M, Yurimoto S, Fujimoto T, Ohara N, et al. Identification and characterization of PRG-1 as a neuronal calmodulin-binding protein. Biochem J. 2010;431(1):81–91.

[54] Brunel N. Dynamics of Sparsely Connected Networks of Excitatory and Inhibitory Spiking Neurons. J Comput Neurosci. 2000;8:183–208.

[55] Medeiros ES, Caldas IL, Baptista MS, Feudel U. Trapping phenomenon attenuates the consequences of tipping points for limit cycles. Scientific reports. 2017;7(1):1–11.

[56] Loebel A, Tsodyks M. Computation by ensemble synchronization in recurrent networks with synaptic depression. Journal of computational neuroscience. 2002;13(2):111–124.

[57] Mi Y, Katkov M, Tsodyks M. Synaptic Correlates of Working Memory Capacity. Neuron. 2017;93(2):323–330.

[58] Nordlie E, Gewaltig MO, Plesser HE. Towards reproducible descriptions of neuronal network models. PLoS Comput Biol. 2009;5(8):e1000456.

[59] Miller EK, Lundqvist M, Bastos AM. Working Memory 2.0. Neuron. 2018;100(2):463–475.

[60] Sreenivasan KK, D'Esposito M. The what, where and how of delay activity. Nature Reviews Neuroscience. 2019;20(8):466–481.

[61] Pittà MD, Ben-Jacob E, Berry H. Astrocytic theory of working memory. BMC Neuroscience. 2014;15:P206.

[62] Kolata S, Wu J, Light K, Schachner M, Matzel LD. Impaired working memory duration but normal learning abilities found in mice that are conditionally deficient in the close homolog of L1. J Neurosci. 2008;28(50):13505–13510.

[63] Vogt J, Kirischuk S, Unichenko P, Schlüter L, Pelosi A, Endle H, et al. Synaptic Phospholipid Signaling Modulates Axon Outgrowth via Glutamate-dependent Ca2+-mediated Molecular Pathways. Cereb Cortex. 2017;27(1):131–145.
