# Supplementary material for: Modulation of working memory duration by synaptic and astrocytic mechanisms
Source: PLoS Comput Biol. 2022 Oct 3;18(10):e1010543. doi: 10.1371/journal.pcbi.1010543 (PMC9560596; doi:10.1371/journal.pcbi.1010543)
Supplement: Attachment — Submitted filename: PCOMPBIOL-D-21-02324R1.Becker.pdf [file pcbi.1010543.s004.pdf]

PCOMPBIOL-D-21-02324 R1
Title: How synaptic and astrocytic mechanisms can modulate working memory durations
Authors: S. Becker, A. Nold, and T. Tchumatchenko

General considerations
I appreciate the substantial effort put by the authors in editing their manuscript. I found it remarkably improved in clarity and quality in its new version. There are still some issues that I believe require minor-to-moderate editing. I am detailing my suggestions for editing below, referring to the manuscript with tracked changes provided in the supplementary. I may anticipate these authors' frustration with yet another round of reviews. However, I want to reassure them that it is the last one.

Suggestion for editing

I am not enthusiastic about the new title. I advise replacing it with something more concise and, possibly more general, like "*Modulation of working memory duration by synaptic and astrocytic mechanisms*."

A further aspect that is unclear from your model setup is how you connect synapses with astrocytes and vice versa. If my understanding is correct, you consider each synapse to be independently stimulated and be modulated by an astrocyte, or all synapses to stimulate a "lumped" astrocyte and be modulated thereby. In the case of the first scenario, I believe you do not consider any connection between astrocytes. Your modeling assumptions should be clarified in the Model description (with a couple of sentences). I would also complement the Discussion section with an additional paragraph commenting on the possible role of synapse-to-astrocyte and vice versa connections (generally called heterotypic connections) in your network model.

Eqs. 1–3; and L115. Simplify your notation for indexed time instants, moving index "k" to subscript, i.e. $t^{(k)} \rightarrow t_k$; $t^{(k),-}$ or $t^{(k),+} \rightarrow t_k^-$ and t_k^+, respectively. Introduce $r(t_k) = y_k x_k$ for your release. On L117 you define this latter product as a "rate" which is not correct. It is just instantaneous release of synaptic resources.

The line immediately after L152. Please, as is the case for the TM model, include here a reference to De Pitta's model for gliomodulation. Specifically, along with your current reference [31], you should include De Pitta, Comput. Gliosci. Ch 10, 2019. This will not detract from the originality of your work. Instead, you show how that model, initially introduced by De Pitta, Berry, and co-workers to study glutamatergic gliotransmission, could be used to effectively model LPA-mediated modulations of synaptic release, which, it could be argued, are conceptually different from glutamatergic glial modulation. Accordingly, modify your first line in this paragraph, by

"*We integrate the LPA mechanism into STP following the original approach introduced by De Pitta and co-workers [31,XX]. That is, we consider a variable ℓ ...*"

L177: transmission-dependent → activity-dependent
L189: ...coexist with a stable...
L192: Mistyped newline.

L204: Modify section title as: "Noise can turn transient WM into activity-silent or persistent firing regimes.

L205—L206: Drop. Start from “To identify...”
L207—L208: use numbers to improve readibility: ... with three different sources of noise: 1. noise in ther amount of ...; 2. noise4 in the amount of ...; and 3. noise in the network...

L200—L221: Move ordinals before items: ... the phase space: (i) in the region of ...., and (ii) in the bistability region.

L227: “...fixed pint regime (1).” I don’t understand that “(1).” I suppose it is a typo, in that case remove it.

L258: Split the sentence: “(Figure S6). That is, negative...”

L299: “un-cued”: maybe “spontaneous?”

L294—L296: Move ordinals before items and correct grammar: “...shows that for a sufficiently strong cue, the transients appear in the neighbour of the bifurcation, between (i) the fixed point, and (ii) bistable regions along *U* (highlighted in grey).”

L392—L398: There is something broken in the logical flow of this part. It does not flow right. Specifically,, on L394 you talk about “spiking networks,” but it is not clear what spiking networks. I assume “*our* spiking network *models*” maybe?

L456: In their model → In that network.

L471: .... could switch the WM regime from activity-silent to activity-persistent [NEWREF]. Instead of [70] use the more recent work by the same author(s) available on bioRxiv: see: https://www.biorxiv.org/content/10.1101/2021.03.25.436819v1

Figure editing
The main issues which require moderate editing, are with the present figures.

Figure 1: In panel B, the \ell is almost indistinguishable from “1” (one). Please use the same notation as in the text, i.e. \ell or \mathcal{l} for the LPA-variable.

Figure 2B: Please, vertically align all panels, so that the X axes are on the same level. Additionally, make sure that all panels (plots) are of the same size. Improve labels: Use standard abbreviations: i.e. ‘s’ or ‘sec’ for seconds rather than ‘secs.’ In Panel A: I don’t see the reason to repeat the Y-axis for all plots when no ticks are present: I would drop them, and have the Y-axis shared among the three panels .

Figure 3: Panels C1 and C2: use the same view for these 3D plots, and draw trajectories and limits cycles, by thicker lines. Adjust axis labels in the X and Y axes, so that they follow the angles of the respective axes: this will minimize crowding. Do label all axes – the current choice of using “Calcium binding, y” for two figures, is not great.

Figure 4: Panel B.1: add an histogram as in Panel A.1. Panels A.2, A.3, B.2, and B.3: Improve visibility of the input including the cue.

Figure 5: Panels A.1 and B.1: Add histograms for distributions as in Panel A.1 from Figure 4. Improve visibility of the cue.

Figure 6: Panel B.1: Add histogram of the distribution as in Panel A.1 from Figure 4. Add legends for colors in the top plot (e.g. Panel A.2) as is the case for precedingfigures. Improve visibility of the cue.
