# Supplementary material for: Modulation of working memory duration by synaptic and astrocytic mechanisms
Source: PLoS Comput Biol. 2022 Oct 3;18(10):e1010543. doi: 10.1371/journal.pcbi.1010543 (PMC9560596; doi:10.1371/journal.pcbi.1010543)
Supplement: Attachment — Submitted filename: Reply_round2.pdf [file pcbi.1010543.s005.pdf]

Synaptic and astrocytic modulation of working memory durations

Sophia Becker, Andreas Nold, Tatjana Tchumatchenko

Dear editors, dear reviewers,

We are grateful to the reviewers for their positive feedback, their appreciation of our work and especially for their valuable, constructive comments during this and the previous revision which have further improved our manuscript.

We marked the comments made by the reviewers in gray, followed by our point-by-point responses with an indication of the corresponding changes in the manuscript in italics.

Reviewer 1 supports the publication of our article and had no further comments.
Reviewer 2 was also satisfied with our previous revision and had some minor/moderate stylistic and clarifying suggestions.
Reviewer 3 was also satisfied with the content and presentation quality of our manuscript and only suggested to add additional points to the discussion.

Reviewer 1

Dear authors, Thank you for addressing all of my comments.

Thank you for the appreciation of our work and your helpful comments in the last review round.

Reviewer 2

I appreciate the substantial effort put by the authors in editing their manuscript. I found it remarkably improved in clarity and quality in its new version. There are still some issues that I believe require minor-to-moderate editing. I am detailing my suggestions for editing below, referring to the manuscript with tracked changes provided in the supplementary. I may anticipate these authors' frustration with yet another round of reviews. However, I want to reassure them that it is the last one.

Thank you for the positive comments and for the suggestions to edit the manuscript. Please see below for our point-by-point responses.

I am not enthusiastic about the new title. I advise replacing it with something more concise and, possibly more general, like 'Modulation of working memory duration by synaptic and astrocytic mechanisms.'

Thank you for the great suggestion, we adopted the proposed title in our revised manuscript.

A further aspect that is unclear from your model setup is how you connect synapses with astrocytes and vice versa. If my understanding is correct, you consider each synapse to be independently stimulated and be modulated by an astrocyte, or all synapses to stimulate a "lumped" astrocyte and be modulated thereby. In the case of the first scenario, I believe you do not consider any connection between astrocytes. Your modeling assumptions should be clarified in the Model description (with a couple of sentences).

Thank you for the helpful comment. In the revised manuscript, we address the points raised by the referee in two ways. First, we included the following additional explanation in the Methods section of the main manuscript:

> *'Note that we consider each synapse to be interacting with an independent astrocytic compartment that does not communicate with astrocytes or astrocytic compartments surrounding other synapses.'*

Second, we extended the explanation given in the SI section, which now reads:

> *'Note that our current PRG spiking network does not model astrocyte-to-astrocyte interactions but assumes that an astrocyte is synapse-specific, i.e. regulates the activity of a single synapse. Vice versa, each synapse receives individual presynaptic activity and interacts with an independent astrocyte or astrocytic compartment. Communication between astrocytes or astrocytic compartments surrounding different synapses is currently not considered in the model. Because we assume all synapses of a sub-population to be statistically identical (except for the connection weights, which can differ) we can define an average synaptic and astrocytic state.'*

I would also complement the Discussion section with an additional paragraph commenting on the possible role of synapse-to-astrocyte and vice versa connections (generally called heterotypic connections) in your network model.

Thank you for the suggestion. In the current manuscript, we discuss the possible impact of the connections between synapses and astrocytes that are established by our mechanism in paragraph 6 and 7 of the discussion. There, we elaborate on the potential roles of this type of synapse-astrocyte communication on top-down volitional WM control and other cognitive processes. In the revised manuscript, we also extended paragraph 3 of the discussion:

> *'Note that the LPA-mediated signaling pathway modelled in this work is only one of a multitude of neuron-astrocyte and synapse-astrocyte communication pathways [29, 50]. To which degree other signaling pathways between synapses and astrocytes have similar or different effects on the network dynamics during WM remains an exciting topic for future research.'*

Eqs. 1–3; and L115. Simplify your notation for indexed time instants, moving index 'k' to subscript, i.e. $t^{(k)} \to t_k$; $t^{(k),-}$ or $t^{(k),+} \to t_k^-$ and t_k^+, respectively. Introduce $r(t_k) = y_k x_k$ for your release. On L117 you define this latter product as a "rate" which is not correct. It is just instantaneous release of synaptic resources.

Thank you for the helpful suggestion. We adopted the subscript notation and the definition of $r(t_k) = y_k x_k$ as instantaneous release of synaptic resources throughout the text and equations in the main manuscript and the supplementary information.

The line immediately after L152. Please, as is the case for the TM model, include here a reference to De Pitta's model for gliomodulation. Specifically, along with your current reference [31], you should include De Pitta, Comput. Gliosci. Ch 10, 2019. This will not detract from the originality of your work. Instead, you show how that model, initially introduced by De Pitta, Berry, and co-workers to study glutamatergic gliotransmission, could be used to effectively model LPA-mediated modulations of synaptic release, which, it could be argued, are conceptually different from glutamatergic glial modulation. Accordingly, modify your first line in this paragraph, by "We integrate the LPA mechanism into STP following the original approach introduced by De Pitta and co-workers [31,XX]. That is, we consider a variable ℓ ..."

Thank you for the comment. Following the referee's suggestion, we now point out the work by De Pitta et al. [31, 50] at the suggested line in the Methods of the main text. We also made sure to add the second reference ([50]) in the other places where we cite De Pitta et al.'s model, e.g. in the beginning of the Methods in the main text:

> *'Building on the STP framework by Tsodyks et al. [18] and inspired by De Pitta et al.'s model of gliomodulation [31, 50], we construct a synapse model (Fig. 1 B) that captures the influence of this astrocytic signaling mechanism on synaptic STP.'*

L177: transmission-dependent→ activity-dependent

Thank you for the suggestion, we adopted our wording throughout the text.

L189: ...coexist with a stable...

Fixed, thanks for spotting the typo.

L192: Mistyped newline.

Thanks for pointing it out, we fixed the typo in the revised manuscript.

L204: Modify section title as: "Noise can turn transient WM into activity-silent or persistent firing regimes."

Thank you for the suggestion. Guided by the referee's suggestion, we rephrased our section title as follows: *'Noise can turn activity-silent and persistent firing regimes into transient WM activity'*. This is to avoid potential misunderstandings about the fact that the transient regime can only be found in networks with noise, while silent and persistent regimes are present both in the networks with and without noise.

L205—L206: Drop. Start from "To identify..."

Thanks for the comment. We followed the referee's suggestion and dropped the first sentence. To avoid ambiguities about the kind of network mechanisms we study, we also rephrased the subsequent sentence, which now reads: *'To identify the network mechanisms by which noise can lead to the stochastic termination of WM representations, we consider STP rate networks (see Fig. 1 D) with three different sources of noise: ...'*

L207—L208: use numbers to improve readibility: ... with three different sources of noise: 1. noise in the amount of ...; 2. noise in the amount of ...; and 3. noise in the network...

Done, thanks for the suggestion.

L200—L221: Move ordinals before items: ... the phase space: (i) in the region of ...., and (ii) in the bistability region.

Good idea, done.

L227: "...fixed pint regime (1)." I don't understand that "(1)." I suppose it is a typo, in that case remove it.

Thanks for the comment, we removed it as suggested.

L258: Split the sentence: "(Figure S6). That is, negative..."

Done, thanks for the suggestion.

L299: 'un-cued': maybe 'spontaneous?'

Thanks for the comment. In the revised manuscript, we adopted the suggested expression: *'free (i.e. spontaneous, not cued) recall of WM items'*.

L294—L296: Move ordinals before items and correct grammar: '...shows that for a sufficiently strong cue, the transients appear in the neighbour of the bifurcation, between (i) the fixed point, and (ii) bistable regions along U (highlighted in grey).'

Done, thanks for the comment.

L392—L398: There is something broken in the logical flow of this part. It does not flow right. Specifically, on L394 you talk about 'spiking networks,' but it is not clear what spiking networks. I assume 'our spiking network models' maybe?

Thank you for the helpful comment. We adjusted the respective lines as follows:

> *Our analysis of the different noise components suggests that in STP firing rate networks, adding noise to the population activity E tends to stabilize transient cycles (see Figure S6), whilst noise in the synaptic variable y tends to have a disruptive effect (see Figures S8). In our STP spiking networks, we identified connectivity noise (also: 'frozen noise' [62]) as an important source of variability that influences the termination of population activity during WM delays (Figure S16 and Figure S17 C, D).*

L456: In their model $\rightarrow$ In that network.

Done, thank you for the comment.

L471: .... could switch the WM regime from activity-silent to activity-persistent [NEWREF]. Instead of [70] use the more recent work by the same author(s) available on bioRxiv: see: https://www.biorxiv.org/content/10.1101/2021.03.25.436819v1

Thank you for the comment, we corrected the typo and now cite the correct reference to [68].

The main issues which require moderate editing, are with the present figures. Figure 1: In panel B, the ℓ is almost indistinguishable from '1' (one). Please use the same notation as in the text, i.e. ℓ or $\updownarrow$ for the LPA-variable.

Thank you for the comment, we corrected the notation of the variable ℓ in the revised Figure 1. We also made sure to use consistent notation of ℓ in the remaining text.

Figure 2B: Please, vertically align all panels, so that the X axes are on the same level. Additionally, make sure that all panels (plots) are of the same size. Improve labels: Use standard abbreviations: i.e. 's' or 'sec' for seconds rather than 'secs.' In Panel A: I don't see the reason to repeat the Y-axis for all plots when no ticks are present: I would drop them, and have the Y-axis shared among the three panels.

Thank you for the comments. In the revised Figure 2, we have aligned the panels and adjusted their size such that the outer boundaries of panel 2B match the outer boundaries of panel A (upper boundary: box around middle panel in A). We have also changed the abbreviation of 'secs' that was suggested by a different referee in the first round of revisions to 'sec'. Regarding the repetition of the y-axis in panel A: We outlined the y-axis multiple times to visually emphasize the fact that the simulations in each panel arise from individual network simulations with different network parameters. We would therefore like to keep it as it is for now.

Figure 3: Panels C1 and C2: use the same view for these 3D plots, and draw trajectories and limits cycles, by thicker lines. Adjust axis labels in the X and Y axes, so that they follow the angles of the respective axes: this will minimize crowding. Do label all axes – the current choice of using 'Calcium binding, y' for two figures, is not great.

Thank you for the suggestions. We adjusted the line thickness and axis labels in Figure 3C as suggested and added axes labels for the y variable such that every plot is self-consistent. Regarding the perspective of the 3D plots: The view angle of each plot has been optimized to allow the most unambiguous and clear perspective on the trajectories plotted in each. To ensure the best possible readability of both panels, we would therefore prefer keeping the current view angles.

Figure 4: Panel B.1: add an histogram as in Panel A.1. Panels A.2, A.3, B.2, and B.3: Improve visibility of the input including the cue.

Thank you for this comment. The histograms in the inset of panels A.1 of Figures 4, 5 and 6 represent results for 100 rate model simulations, as compared to 15-30 simulations which are shown in the scatterplot. The reason for this is that in the scatterplot, results for different values of $U_b/\ \tau_L$ are shown, and performing 100 computations for each value would multiply the computational costs accordingly. In panels B.1, we show results of spiking network simulations, which are computationally much more expensive than rate model simulations. Therefore, we did not perform 100 simulations for the spiking network scenarios in Figures 4 and 6. Showing histograms based on only 15 values (see Figs 4 and 6) would lead to a high degree of variability and low accuracy, and a comparability with the rate model histogram would not be given. In order to address the issue of comparability between the rate and the spiking network simulations, we have therefore plotted both rate and spiking network results as stripplots, i.e. we have included a small random jitter in the x-direction. This reduces the overlay of points, therefore giving a better impression of the distribution at each value of $U_b/\ \tau_L$.

Concerning the visibility of the cue, we have increased the size of the cue, included a y-label, and included an arrow to the position of the cue for all corresponding plots of Figures 4-6.

Figure 5: Panels A.1 and B.1: Add histograms for distributions as in Panel A.1 from Figure 4. Improve visibility of the cue.

See above.

Figure 6: Panel B.1: Add histogram of the distribution as in Panel A.1 from Figure 4. Add legends for colors in the top plot (e.g. Panel A.2) as is the case for preceding figures. Improve visibility of the cue.

Thank you for this comment concerning the legends for colors, this must have slipped through. We have added the legends in panel A.2 accordingly. Concerning histogram and cue: See above.

Reviewer 3

I think the authors have provided strong responses to my questions and I find the manuscript has been greatly improved. I only have some remaining points for authors to think about in a larger picture and decide how they want to interpret their results and put these into an experimental context.

Thank you for your positive comments and for your suggestions and questions regarding the greater context of our work. We address each point and the resulting changes in the revised manuscript below.

Regarding my question in the previous round on whether the long time scale integration could result in the network becoming 'stuck' in the persistent state, I am satisfied with the answers from the authors regarding the persistent state but I have a follow-up question. If we take the example I brought up with the cDNMS task where the animal is presented with a long sequence of items, could your long time scales lead to a slow accumulation of your quantity l? See for instance your new text in Discussion, line 417 'We can think of LPA as a slow integrator of past synaptic activity.' Suppose the cDNMS task presented to the animal changes, lets say to a task where dynamics stemming from high l would not be beneficial, would the slow kinetics not prevent a rapid and flexible adaptation to the new requirements and instead leaving the animal behaviourally 'stuck' in the high-l-state? Any biochemical pathways leading to the (rapid) breakdown of LPA would open up for a decrease of τ_L and resolve this issue I presume.

Thank you for raising this interesting point. A multi-item or multi-task WM experiment like the one you describe could be translated into the context of our model as a sequential activation of multiple, potentially overlapping 'selective' excitatory populations, each of them representing the engram encoding one task or item (similar to the setting used in Mi et al. [67] but with LPA signaling in E-E synapses). When there is no overlap between the selective populations of two sequentially presented tasks or stimuli, a high LPA binding value (ℓ) after termination of WM activity in the first selective population will not affect the WM representation in the other population, in which the LPA binding is still close to zero. This is because (i) high LPA binding in a subpopulation does not affect the network activity except if that specific subpopulation is presented with a cue, (ii) WM activity in one subpopulation only leads to LPA ramping in that subpopulation but not in the rest of the network, and (iii) the ramping of LPA is bounded by its equilibrium value, i.e. even when a stimulus is presented for very long or repeatedly, the amount of accumulated LPA will never exceed that equilibrium value. The bounding by an equilibrium value, results from our model assumption that there is a limited number of LPA binding slots. Hence, as more LPA is bound, the probability of a further binding event decreases due a decreasing number of free slots, while the probability of an unbinding event happening increases simultaneously. When we consider the scenario that the selective populations of two sequentially presented tasks/stimuli are overlapping, the high residual LPA binding due to the first task/stimulus could indeed affect the WM dynamics of the second task/stimulus. In particular, for stimuli that have a large overlap with recently presented stimuli, the WM dynamics start close to the equilibrium value of LPA (ℓ), which would slightly bias the distribution of WM durations towards longer durations. However, this is not necessarily a sign that the brain would get behaviourally 'stuck' but could imply an experimentally testable hypothesis of perceptual 'priming', in the sense that previously remembered or attended stimuli might be more easily kept in WM.

To address the referee's comment, we integrated a short discussion of the topic in the Discussion of the revised manuscript:

> *'Whilst the LPA unbinding timescale has a long-term stabilizing effect on synaptic WM, there are three reasons why it does not cause the WM dynamics to get stuck. First, even in the case of strong LPA signaling, the LPA-stabilized persistent WM representations can be terminated by a single, weak off-stimulus, just like in the case of regular persistent WM representations. Second, since the activity-dependent ramping of LPA is similarly slow as the decay of LPA-mediated effects,*

there is no limitation to follow faster WM protocols. For a fast WM protocol, the slow effects of LPA signaling would probably be only marginally observable since they start showing effect only several seconds after the cue offset. Third, even very long or repeated presentation of a WM stimulus does not affect the WM activity of subsequently presented, very different WM stimuli (i.e. with no overlap between encoding populations). This is because the amount of presynaptically bound LPA only ramps up within the stimulus-encoding population, and, due to the limitation of LPA binding slots, only increases up to its bounded equilibrium value. As a result, it does not affect the activity in the remaining network after termination of WM activity. It is possible, however, that LPA ramping helps stabilize WM activity of very similar, subsequently presented stimuli or tasks (i.e. with a significant overlap of representations). The verification of this hypothesis and the detailed analysis of network behaviour in a multi-item WM setting with LPA signaling remains to be tested in future work.'

Moreover, we clarified the point that the ramping of LPA is bounded by its equilibrium value:

'... the amount of presynaptically bound LPA ramps up slowly during the course of the WM activity **until its equilibrium value is reached or the activity terminates**. *We can think of LPA as a slow integrator of* **recent** *synaptic activity which, in this case, saves information about the currently active WM representation.'*

You study how noise can affect and potentially control the activity in the metastable regime. I subscribe to the notion of the brain as an information processing system, and in this regard it is unclear why deepely located structures supporting WM would be transmitting or producing pure noise. Whereas the properties of the noise and your implementation of it in the model is clear, it is thus unclear to me how we should understand this 'noise' from a biological perspective. You provide examples of how calcium and synaptic transmission appears to be guided by fluctuations and this seems fine. However, when it comes to neuronal spike fluctuations, are these really to be seen as 'noise'? Neuronal firing contains a lot of correlations by virtue of neurons in a connected network. Moreover, what differentiates this activity you call noise from activity in your background activity or your cue activity?

Thank you the question. We would like to clarify that we take a 'descriptive' perspective on noise, i.e. we observe the magnitude of fluctuations in the input current to neurons (that can arise from the interplay of different stochastic biological processes in real brain networks) and label the magnitude of these fluctuations (standard deviation) as 'noise'. The mean of the input current is interpreted as the 'signal'. In the context of our paper, 'noise' or the 'strength of noise' is therefore not associated with the mean of network activity but instead with the magnitude of fluctuations around that mean. Following this logic, we interpret the mean input current arising from spontaneous (Poisson-type) spiking activity in the network as a baseline activity signal. Biologically, this baseline activity could represent network activity unrelated to the current WM task. The WM signal or cue is implemented as a change in the mean input current that is large compared to this baseline activity signal. Hence, our findings about 'noise' (i.e. fluctuations about the signal/mean input current) that can impact the metastable activity during the WM task appeal to the concept that cognitive mechanisms like attention or learning can change how robustly (i.e. with how much standard deviation) a certain input signal is represented and transmitted across brain regions. While the scope of our work did not allow us to further investigate the role of spiking correlations due to specific network connectivity, we acknowledge that these are indeed relevant for information transmission in biological networks and an interesting topic for future work.

In the discussion of the revised manuscript, we clarified our interpretation of noise as the standard deviation of a baseline/cue signal rather than a signal in its own right:

> *'We show that noise helps generate transient WM activity at the transition between the silent and the persistent WM regime and can explain the stochasticity of transient WM durations around a mean (Fig. 2 B blue histogram). Importantly, 'noise' in the context of our work refers to the fluctuations of network activity around its mean but does not represent a signal in its own right.'*

We also included the role of spike correlations as a topic for future research:

> *'A detailed analysis of different circuit motives and the impact of resulting spiking correlations on the WM representations lies out of the scope of this work but remains an important direction for follow-up research.'*

Line 317 'longer transient WM representations therefore come with a higher variability of the durations' Your model thus predicts that when you go from neglibile to low to medium LPA signalling, you would observe increasing variability of the length of the transients. Behaviourally this seems to indicate a rather spurious behaviour emerging, which seems an unfavourable functional mechanism. Please put your functional prediction into a behavioural context.

Thank you for the relevant remark. As you correctly noted, our model predicts that, for the case of weak astrocytic signaling (Figure 5), there could be different tasks or brain structures that allow for different precisions of WM duration – and hence more or less spurious behavioural regimes. In contrast, for the case of strong astrocytic signaling, we observe a switch-like behaviour between very short and very long WM durations (filtering) without an increase in variability. While we show which synaptic/astrocytic and network mechanisms would be responsible for these different WM precisions and the filtering, respectively, our work does not allow to draw any conclusions on which behavioural regime is used by animals and/or humans during specific tasks or in different brain regions. We included a short discussion of this topic in the revised manuscript:

> *'In practice, the modulation of WM introduced by LPA signaling could have several functions. First, since the variability of WM durations is dependent on LPA parameters in the weak astrocytic signaling case (Fig. 5), our work predicts that the available 'precision' of WM durations could differ across tasks or brain structures – and hence allow for more or less spurious behavioural regimes. In contrast, for the case of strong astrocytic signaling, we observe a switch-like behaviour between very short and very long WM durations (filtering) without an increase in variability. While we show which synaptic/astrocytic and network mechanisms would be responsible for the spuriousness of WM, our work by itself does not allow to draw conclusions on which behavioural regime would be used by animals or humans during specific tasks or in specific brain regions. '*

Regarding the 'window of vulnerability', line 359-360. Firstly, are you predicting that the influence of distractors would be more influential shortly after presentation of the cue and instead distractors would become less potent after some amount of time? Are you aware of any experiments suporting this prediction? Secondly, maybe we can interpret this feature differently? I find your new text in the Discussion intriguing, lines 481-483 discussing how more salient or more attended stimuli might be stronger when competing with other WM representations. I find this intriguing as it opens up for competitive processeses following e.g. ambigious stimuli where perceptual rivalry leads to one 'winning' representation. So, instead of a 'window of vulnerability' this could be viewed as a 'window of opportunity' to resolve ambigious activity. Mechanisms that could modulate a competitive process including multiple factors such as which pattern appeared first or which is more salient, would be important.

Thank you for the exciting remarks and suggestions. Regarding your first point about the susceptibility to distractors: Our results indeed predict that, with strong astrocytic modulation, WM representations might be more susceptible to distractors during early than late stages of the maintenance period. We are currently not aware of experimental studies explicitly testing the impact of distractors when presented at different time points during the delay period, such that the experimental verification or falsification of this prediction remains the topic of future research. We have extended our discussion section with the following to address the referee's comment:

> *'During this initial 'window of vulnerability', the WM representation is very susceptible to disruption by noise, such that WM representations with slightly higher internal noise would have a high chance to terminate during this period. Such slighty elevated noise could e.g. arise from the presence of distractors or more salient/attended and therefore stronger competing WM representations in the same network. We are currently not aware of experimental studies explicitly testing the impact of distractors that are presented at different time points during the delay period, such that the experimental verification or falsification of this prediction remains the topic of future research.'*

We fully agree with your second point that the 'window of vulnerability' can also be interpreted as a 'window of opportunity' to resolve ambiguous activity. We included this interpretation in the discussion of the revised manuscript:

> *'The filtering implemented by this mechanisms could be relevant for prioritizing and managing the limited mental capacity to hold WM items. In particular, it opens up the possibility of competitive processes between ambiguous stimuli, where multiple factors such as the order of presentation or the salience of a WM item could be important for which WM representation will be the 'winning' one that stays active. In that sense, the 'window of vulnerability' could also be interpreted as a 'window of opportunity' to resolve ambiguous activity.'*

Regarding your new comment on multiple items, I am not convinced it holds. You do use the careful wording 'by a separate neuronal population', line 452-453, and 'separate' is the key word here. If representations of different items overlap, activation of the items may result in the two representations not staying separate but becoming merged, see for instance Warden and Miller, Cerebral Cortex, 2007. As much as I understand you wish to provide a positive response to the question of the reviewer, this might be a slippery slope.

Thank you for the helpful comment. As you correctly pointed out, we did not discuss the multi-item WM scenario with overlapping representations in the previous manuscript. Following your

suggestion, we included it in the discussion of the revised manuscript and clearly distinguish our predictions for the cases of overlapping vs. non-overlapping parallel WM items:

> *'Another exciting direction for future research is to apply our model in a multi-item WM setting similar to the settings explored for synaptic WM with STP [5, 67]. Due to the relatively fast decay of the STP traces, multiple WM items (each encoded by a separate neuronal subpopulation) are kept in memory by sequential firing of a single population spike from each population. We can speculate that, for the case of non-overlapping parallel WM items, the long timescale of astrocytic modulation could allow each memory to be active for an extended time before the next WM item has to be refreshed in memory. This intuition is supported by a preprint [68] that studied multi-item WM in a network with astrocytic modulation of STP. [...] For the case of partially overlapping, simultaneously active WM representations, the effect of the slow astrocytic modulation is more complex and subject of future research. In particular, we can hypothesize that astrocytic signaling could also lead to fusion of overlapping WM representations, depending on the synaptic and astrocytic parameters.'*

Minor: Figure 2A. On the y-axis, You use the unit Hz for 'firing rate'. I more often see Hz used for firing frequency (time or spatially averaged activity) and s^{-1} for rate (instantaneous firing frequency).

Thank you for the comment. We adopted the notation of firing rate in Hz from previous work in the field, e.g. Murray et al. [4] or Mi et al. [67]. Since we would like to stay consistent with these directly related articles, we would therefore prefer to keep the current Hz notation.

Figure 7. 'Incoming current ...' what kind of 'current' is this? Maybe 'activities'?

Thanks for the comment, we changed it to 'activity'.

References

[1] Fuster JM, Alexander GE. Neuron Activity Related to Short-Term Memory. Science. 1971;173(3997):652–654.

[2] Funahashi S, Bruce CJ, Goldman-Rakic PS. Mnemonic Coding of Visual Space in the Monkey's Dorsolateral Prefrontal Cortex. J Neurophysiol. 1989;61(2):331– 349.

[3] Christophel TB, Christiaan Klink P, Spitzer B, Roelfsema PR, Haynes JD. The Distributed Nature of Working Memory. Trends Cogn Sci. 2017;21(2):111–124.

[4] Murray JD, Jaramillo J, Wang XJ. Working Memory and Decision-Making in a Frontoparietal Circuit Model. J Neurosci. 2017;37(50):12167–12186.

[5] Mongillo G, Barak O, Tsodyks M. Synaptic Theory of Working Memory. Science. 2008;319(5869):1543–1546.

[6] Barbosa J, Stein H, Martinez RL, Galan-Gadea A, Li S, Dalmau J, et al. Interplay between persistent activity and activity-silent dynamics in the prefrontal cortex underlies serial biases in working memory. Nat Neurosci. 2020;23:1016–1024.

[7] Harvey CD, Coen P, Tank DW. Choice-specific sequences in parietal cortex during a virtual-navigation decision task. Nature. 2012;484(7392):62–68.

[8] Schmitt LI, Wimmer RD, Nakajima M, Happ M, Mofakham S, Halassa MM. Thalamic amplification of cortical connectivity sustains attentional control. Nature. 2017;545(7653):219–223.

[9] Druckmann S, Chklovskii DB. Neuronal circuits underlying persistent representations despite time varying activity. Curr Biol. 2012;22:2095–2103.

[10] Lisman JE, Jensen O. The theta-gamma neural code. Neuron. 2013;77(6):1002–1016.

[11] Lundqvist M, Rose J, Herman P, Brincat SL, Buschman TJ, Miller EK. Gamma and Beta Bursts Underlie Working Memory. Neuron. 2016;90(1):152–164.

[12] Lundqvist M, Herman P, Warden MR, Brincat SL, Miller EK. Gamma and beta bursts during working memory readout suggest roles in its volitional control. Nat Commun. 2018;9(1):1–12.

[13] Bastos AM, Loonis R, Kornblith S, Lundqvist M, Miller EK. Laminar recordings in frontal cortex suggest distinct layers for maintenance and control of working memory. Proc Natl Acad Sci U S A. 2018;115(5):1117–1122.

[14] Masse NY, Yang GR, Francis Song H, Wang XJ, Freedman DJ. Circuit mechanisms for the maintenance and manipulation of information in working memory. Nat Neurosci. 2019;22:1159–1167.

[15] Stokes MG. 'Activity-silent' working memory in prefrontal cortex: A dynamic coding framework. Trends Cogn Sci. 2015;19(7):394–405.

[16] Trübutschek D, Marti S, Ueberschär H, Dehaene S. Probing the limits of activity-silent non-conscious working memory. Proc Natl Acad Sci U S A. 2019;116(28):14358–14367.

[17] Lundqvist M, Herman P, Miller EK. Working Memory: Delay Activity, Yes! Persistent Activity? Maybe Not. J Neurosci. 2018;38(32):7013–7019.

[18] Tsodyks M, Pawelzik K, Markram H. Neural Networks with Dynamic Synapses. Neural Comput. 1998;10(4):821–835.

[19] Lundqvist M, Herman P, Lansner A. Theta and gamma power increases and alpha/beta power decreases with memory load in an attractor network model. Journal of cognitive neuroscience. 2011;23(10):3008–3020.

[20] Trübutschek D, Marti S, Ojeda A, King JR, Mi Y, Tsodyks M, et al. A theory of working memory without consciousness or sustained activity. Elife. 2017;6:e23871.

[21] Zucker RS, Regehr WG. Short-Term Synaptic Plasticity. Annu Rev Physiol. 2002;64(1):355–405.

[22] Fiebig F, Lansner A. A spiking working memory model based on hebbian short-term potentiation. J Neurosci. 2017;37(1):83–96.

[23] Manohar SG, Zokaei N, Fallon SJ, Vogels TP, Husain M. Neural mechanisms of attending to items in working memory. Neuroscience & Biobehavioral Reviews. 2019;101:1–12.

[24] Tabak J, Senn W, O'Donovan MJ, Rinzel J. Modeling of spontaneous activity in developing spinal cord using activity-dependent depression in an excitatory network. Journal of Neuroscience. 2000;20(8):3041–3056.

[25] Bart E, Bao S, Holcman D. Modeling the spontaneous activity of the auditory cortex. Journal of computational neuroscience. 2005;19(3):357–378.

[26] Holcman D, Tsodyks M. The emergence of up and down states in cortical networks. PLoS computational biology. 2006;2(3):e23.

[27] Barak O, Tsodyks M. Persistent Activity in Neural Networks with Dynamic Synapses. PLoS Comput Biol. 2007;3(2):e35.

[28] Cortes JM, Desroches M, Rodrigues S, Veltz R, Muñoz MA, Sejnowski TJ. Short-term synaptic plasticity in the deterministic Tsodyks-Markram model leads to unpredictable network dynamics. Proc Natl Acad Sci U S A. 2013;110(41):16610–16615.

[29] Araque A, Carmignoto G, Haydon PG, Oliet SHR, Robitaille R, Volterra A. Gliotransmitters Travel in Time and Space. Neuron. 2014;81:728–739.

[30] Covelo A, Araque A. Lateral regulation of synaptic transmission by astrocytes. Neuroscience. 2016;323:62=66.

[31] De Pitta M, Brunel N, Volterra A. Astrocytes: Orchestrating synaptic plasticity? Neuroscience. 2016;323:46–61.

[32] Araque A, Rita VP, Sanzgiri P, Haydon PG. Tripartite synapses: glia, the unacknowledged partner. Trends in Neurosciences. 1999;22:208–215.

[33] Wei D, Lee K, Li H, Lu R, Bae JA, Liu Z, et al. AxonEM Dataset: 3D Axon Instance Segmentation of Brain Cortical Regions. In: de Bruijne M, Cattin PC, Cotin S, Padoy N, Speidel S, Zheng Y, et al., editors. Medical Image Computing and Computer Assisted Intervention – MICCAI 2021. Springer International Publishing; 2021. p. 175–185.

[34] Jourdain P, Bergersen LH, Bhaukaurally K, Bezzi P, Santello M, Domercq M, et al. Glutamate exocytosis from astrocytes controls synaptic strength. Nat Neurosci. 2007;10:331–339.

[35] Perea G, Yang A, Boyden ES, Sur M. Optogenetic astrocyte activation modulates response selectivity of visual cortex neurons in vivo. Nature Communications. 2014;5.

[36] Perea G, Araque A. Astrocytes Potentiate Transmitter Release at Single Hippocampal Synapses. Science. 2007;317:1083–1086.

[37] Navarrete M, Perea G, de Sevilla DF, Gómez-Gonzalo M, Núñez A, Martín ED, et al. Astrocytes Mediate In Vivo Cholinergic-Induced Synaptic Plasticity. PLOS Biology. 2012;10:e1001259.

[38] Allen NJ, Eroglu C. Cell Biology of Astrocyte-Synapse Interactions. Neuron. 2017;96(3):697–708.

[39] Durkee CA, Araque A. Diversity and Specificity of Astrocyte–neuron Communication. Neuroscience. 2019;396:73–78.

[40] Santello M, Toni N, Volterra A. Astrocyte function from information processing to cognition and cognitive impairment. Nat Neurosci. 2019;22:154–166.

[41] Tsodyks M. Activity-dependent transmission in neocortical synapses. In: Chow C, Gutkin B, Hansel D, Meunier C, Dalibard J, editors. Methods and Models in Neurophysics. vol. 80 of Les Houches. Elsevier; 2005. p. 245–247, 249–265.

[42] Trimbuch T, Beed P, Vogt J, Schuchmann S, Maier N, Kintscher M, et al. Synaptic PRG-1 Modulates Excitatory Transmission via Lipid Phosphate-Mediated Signaling. Cell. 2009;138(6):1222–1235.

[43] Unichenko P, Kirischuk S, Yang JW, Baumgart J, Roskoden T, Schneider P, et al. Plasticity-related gene 1 affects mouse barrel cortex function via strengthening of glutamatergic thalamocortical transmission. Cereb Cortex. 2016;26(7):3260–3272.

[44] Thalman C, Horta G, Qiao L, Endle H, Tegeder I, Cheng H, et al. Synaptic phospholipids as a new target for cortical hyperexcitability and E/I balance in psychiatric disorders. Nat Mol Psychiatry. 2018;23(8):1699–1710.

[45] Bräuer AU, Savaskan NE, Kühn H, Prehn S, Ninnemann O, Nitsch R. A new phospholipid phosphatase, PRG-1, is involved in axon growth and regenerative sprouting. Nat Neurosci. 2003;6(6):572–578.

[46] Yizhar O, Fenno LE, Prigge M, Schneider F, Davidson TJ, Ogshea DJ, et al. Neocortical excitation/inhibition balance in information processing and social dysfunction. Nature. 2011;477(7363):171–178.

[47] Vogt J, Yang JW, Mobascher A, Cheng J, Li Y, Liu X, et al. Molecular cause and functional impact of altered synaptic lipid signaling due to a prg-1 gene SNP. EMBO Mol Med. 2016;8(1):25–38.

[48] Mongillo G, Hansel D, van Vreeswijk C. Bistability and Spatiotemporal Irregularity in Neuronal Networks with Nonlinear Synaptic Transmission. Phys Rev Lett. 2012 Apr;108:158101. Available from: `https://link.aps.org/doi/10.1103/PhysRevLett.108.158101`.

[49] Hansel D, Mato G. Short-Term Plasticity Explains Irregular Persistent Activity in Working Memory Tasks. Journal of Neuroscience. 2013:133–149.

[50] De Pittà M, Berry H, editors. Computational Glioscience. Springer; 2019.

[51] Berridge MJ, Bootman MD, Roderick HL. Calcium signalling: dynamics, homeostasis and remodelling. Nat Rev Mol Cell Biol. 2003;4(7):517–529.

[52] Keune WJ, Hausmann J, Bolier R, Tolenaars D, Kremer A, Heidebrecht T, et al. Steroid binding to Autotaxin links bile salts and lysophosphatidic acid signalling. Nat Commun. 2016;7(1):11248.

[53] Tokumitsu H, Hatano N, Tsuchiya M, Yurimoto S, Fujimoto T, Ohara N, et al. Identification and characterization of PRG-1 as a neuronal calmodulin-binding protein. Biochem J. 2010;431(1):81–91.

[54] Brunel N. Dynamics of Sparsely Connected Networks of Excitatory and Inhibitory Spiking Neurons. J Comput Neurosci. 2000;8:183–208.

[55] Medeiros ES, Caldas IL, Baptista MS, Feudel U. Trapping phenomenon attenuates the consequences of tipping points for limit cycles. Scientific reports. 2017;7(1):1–11.

[56] Loebel A, Tsodyks M. Computation by ensemble synchronization in recurrent networks with synaptic depression. Journal of computational neuroscience. 2002;13(2):111–124.

[57] Watanabe K, Funahashi S. Neural mechanisms of dual-task interference and cognitive capacity limitation in the prefrontal cortex. Nat Neurosci. 2014;17(4):601–611.

[58] LaRocque JJ, Lewis-Peacock JA, Postle BR. Multiple neural states of representation in short-term memory? It's a matter of attention. Front Hum Neurosci. 2014;8:5.

[59] Fischer C, Endle H, Schumann L, Wilken-Schmitz A, Kaiser J, Gerber S, et al. Prevention of age-associated neuronal hyperexcitability with improved learning and attention upon knockout or antagonism of LPAR2. Cellular and Molecular Life Sciences. 2021;78:1029–1050.

[60] Wei Y, Koulakov AA. Long-term memory stabilized by noise-induced rehearsal. Journal of Neuroscience. 2014;34:15804–15815.

[61] Freeman WJ, Chang HJ, Burke BC, Rose PA, Badler J. Taming chaos: Stabilization of aperiodic attractors by noise. IEEE Transactions on Circuits and Systems I: Fundamental Theory and Applications. 1997;44:989–996.

[62] Seeholzer A, Deger M, Gerstner W. Stability of working memory in continuous attractor networks under the control of short-term plasticity. PLoS Comput Biol. 2019;15(4):e1006928.

[63] Mastrogiuseppe F, Ostojic S. Linking Connectivity, Dynamics, and Computations in Low-Rank Recurrent Neural Networks. Neuron. 2018;99(3):609–623.e29.

[64] Schuessler F, Dubreuil A, Mastrogiuseppe F, Ostojic S, Barak O. Dynamics of random recurrent networks with correlated low-rank structure. Phys Rev Res. 2020;2(1):13111.

[65] Hu Y, Brunton SL, Cain N, Mihalas S, Kutz JN, Shea-Brown E. Feedback through graph motifs relates structure and function in complex networks. Phys Rev E. 2018;98(6):1–25.

[66] Gordleeva SY, Tsybina YA, Krivonosov MI, Ivanchenko MV, Zaikin AA, Kazantsev VB, et al. Modeling Working Memory in a Spiking Neuron Network Accompanied by Astrocytes. Frontiers in Cellular Neuroscience. 2021;15.

[67] Mi Y, Katkov M, Tsodyks M. Synaptic Correlates of Working Memory Capacity. Neuron. 2017;93(2):323–330.

[68] Pittá MD, Brunel N. Multiple forms of working memory emerge from synapse-astrocyte interactions. bioRxiv; 2021. Available from: https://doi.org/10.1101/2021.03.25.436819.

[69] Miller EK, Lundqvist M, Bastos AM. Working Memory 2.0. Neuron. 2018;100(2):463–475.

[70] Sreenivasan KK, D'Esposito M. The what, where and how of delay activity. Nature Reviews Neuroscience. 2019;20(8):466–481.

[71] Vogt J, Kirischuk S, Unichenko P, Schlüter L, Pelosi A, Endle H, et al. Synaptic Phospholipid Signaling Modulates Axon Outgrowth via Glutamate-dependent Ca2+-mediated Molecular Pathways. Cereb Cortex. 2017;27(1):131–145.

[72] Markram H, Muller E, Ramaswamy S, Reimann MW, Abdellah M, Sanchez CA, et al. Reconstruction and Simulation of Neocortical Microcircuitry. Cell. 2015;163(2):456–492.

[73] Nordlie E, Gewaltig MO, Plesser HE. Towards reproducible descriptions of neuronal network models. PLoS Comput Biol. 2009;5(8):e1000456.

[74] Wang Y, Markram H, Goodman PH, Berger TK, Ma J, Goldman-Rakic PS. Heterogeneity in the pyramidal network of the medial prefrontal cortex. Nature Neuroscience. 2006;9:534–542.

[75] Saviane C, Silver RA. Fast vesicle reloading and a large pool sustain high bandwidth transmission at a central synapse. Nature. 2006;439:983–987.

[76] Zenke F, Agnes EJ, , Gerstner W. Diverse synaptic plasticity mechanisms orchestrated to form and retrieve memories in spiking neural networks. Nature Communications. 2015;6:6922.

[77] De Pitta M, Brunel N. Modulation of Synaptic Plasticity by Glutamatergic Gliotransmission: A Modeling Study. Neural Plasticity. 2016;2016:7607924.

[78] Kolata S, Wu J, Light K, Schachner M, Matzel LD. Impaired working memory duration but normal learning abilities found in mice that are conditionally deficient in the close homolog of L1. J Neurosci. 2008;28(50):13505–13510.
